# Beyond Percolation: Graphene‐Enabled Network Reinforcement Enhances Thermal Transport in Paraffin Phase‐Change Composites

**DOI:** 10.1002/advs.75791

**Published:** 2026-05-26

**Authors:** Thomas Hoke, Jackson Hoke, Shucheng Guo, Brittany Chan, Xi Chen

**Affiliations:** ^1^ Department of Electrical and Computer Engineering University of California Riverside California USA; ^2^ Department of Computer Science and Engineering University of California Riverside California USA

**Keywords:** expanded graphite, finite‐volume modeling, graphene nanoplatelets, paraffin, phase change materials, thermal conductivity, X‐ray microCT

## Abstract

Paraffin phase‐change materials store thermal energy through latent heat, making them attractive for thermal management and energy storage applications. However, their low thermal conductivity (κ) limits heat transfer rates and restricts rapid charging–discharging cycles. Achieving substantial κ enhancements at low filler loadings, without degrading phase‐change enthalpy, remains a critical challenge. Here we show that expanded‐graphite (EG) worms and graphene nanoplatelets (GNPs) act synergistically in paraffin composites at low loadings (≤5wt%). At 5wt% filler, 1:1 EG–GNP hybrids raise κ from 0.25 to $2.7\;W\;m^{-1}\;K^{-1}$, outperforming single‐filler composites while preserving the melting window and latent heat. Microscopy suggests that GNPs suppress EG breakup during processing and reinforce surviving worms, and micro‐computed tomography (microCT) reveals a percolating EG backbone that spans the composite. To connect microstructure to thermal transport, a microCT‐informed, resolution‐aware 3D modeling framework was developed. Within this framework, a graphene‐enabled network‐reinforcement mechanism is proposed: graphene nanoplatelet reinforcement of the EG network effectively enhances intraworm connectivity and redistributes heat flux at local constrictions. This mechanism surpasses predictions based solely on percolation geometry or filler fraction and establishes a quantitative design principle for high‐performance hybrid phase‐change composites.

## Graphical abstract

Expanded‐graphite/graphene‐nanoplatelet hybrids deliver a near‐order‐of‐magnitude thermal‐conductivity enhancement in paraffin phase‐change materials. A microCT‐informed 3D modeling framework resolves the percolating EG backbone and captures sub‐voxel GNP enrichment, quantitatively linking microstructure to heat flow and revealing a graphene‐enabled reinforcement mechanism that strengthens transport through the spanning network.

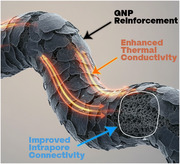

## 1 Introduction

Rising thermal loads are creating a rapidly growing need for thermal management in systems where temperature spikes degrade performance, efficiency, or lifetime [1]. This need is especially clear in applications that must passively buffer heat under tight power and packaging constraints, such as battery packs and power modules during fast transients, photovoltaic platforms exposed to intense solar loading, and building envelopes subject to large diurnal swings [2, 3, 4, 5, 6]. Phase‐change materials (PCMs) address this challenge by absorbing heat as latent energy during melting and releasing it during solidification, enabling large thermal buffering in a compact form factor [7, 8, 9]. Paraffin‐based PCMs are particularly attractive because they combine high latent‐heat density with chemical stability, broad availability, and low cost, but their low thermal conductivity (κ) can slow heat redistribution and leave latent heat underutilized, motivating composite strategies that increase effective κ without sacrificing latent‐heat capacity or practical processability [10, 11, 12, 13].

A common strategy to increase the thermal conductivity of paraffin PCMs is to introduce high‐κ fillers that create more efficient heat‐flow pathways [14]. Broadly, composite designs span two limiting regimes. In one, micrometer‐ and nanometer‐scale additives act primarily as dispersed conductors that raise the effective matrix conductivity, but gains are often modest at low to moderate filler fraction (ϕ) because heat must repeatedly cross poorly coupled filler–matrix contacts and traverse disconnected clusters, with imperfect dispersion further limiting performance [15, 16, 17, 18]. In the other, thermal conductivity increases sharply once a percolated architecture forms a continuous backbone that enables long‐range connectivity through the PCM, as in graphite and expanded‐graphite (EG) paraffin composites and in PCM infiltrated into porous foams and aerogels [19, 20, 21, 22, 23, 24]. However, single‐filler approaches often face a persistent tradeoff: the loadings needed for large κ gains can dilute latent heat and hinder processing, motivating hybrid architectures that combine a percolating scaffold with a finer secondary phase intended to bridge gaps and reduce transport bottlenecks [25, 26, 27, 28].

EG–graphene nanoplatelet (GNP) hybrids represent a promising strategy for enhancing thermal transport because they combine complementary length scales, with EG worms providing extended pathways and GNPs providing a dense population of smaller conductive elements. Although prior studies report that adding GNPs can improve κ relative to EG‐only baselines, the microscopic transport mechanism responsible for this synergy remains unresolved, largely because κ(ϕ) trends and two‐dimensional imaging cannot identify which structural pathways carry heat [29, 30, 31, 32]. Discriminating among candidate mechanisms therefore requires resolving the three‐dimensional (3D) architecture and modeling heat flow directly through it, for example using microCT or focused ion beam‐scanning electron microscopy (FIB‐SEM) coupled to image‐based transport simulation. Yet most prior image‐based thermal‐transport modeling has focused on systems where the load‐bearing phase is tomographically resolvable, an assumption that breaks down for hybrids in which the secondary filler is nanoscale and influential pathways lie below the voxel size, necessitating resolution‐aware representations that can unify both length scales within a single transport model [33, 34, 35, 36, 37, 38].

**FIGURE 1:**
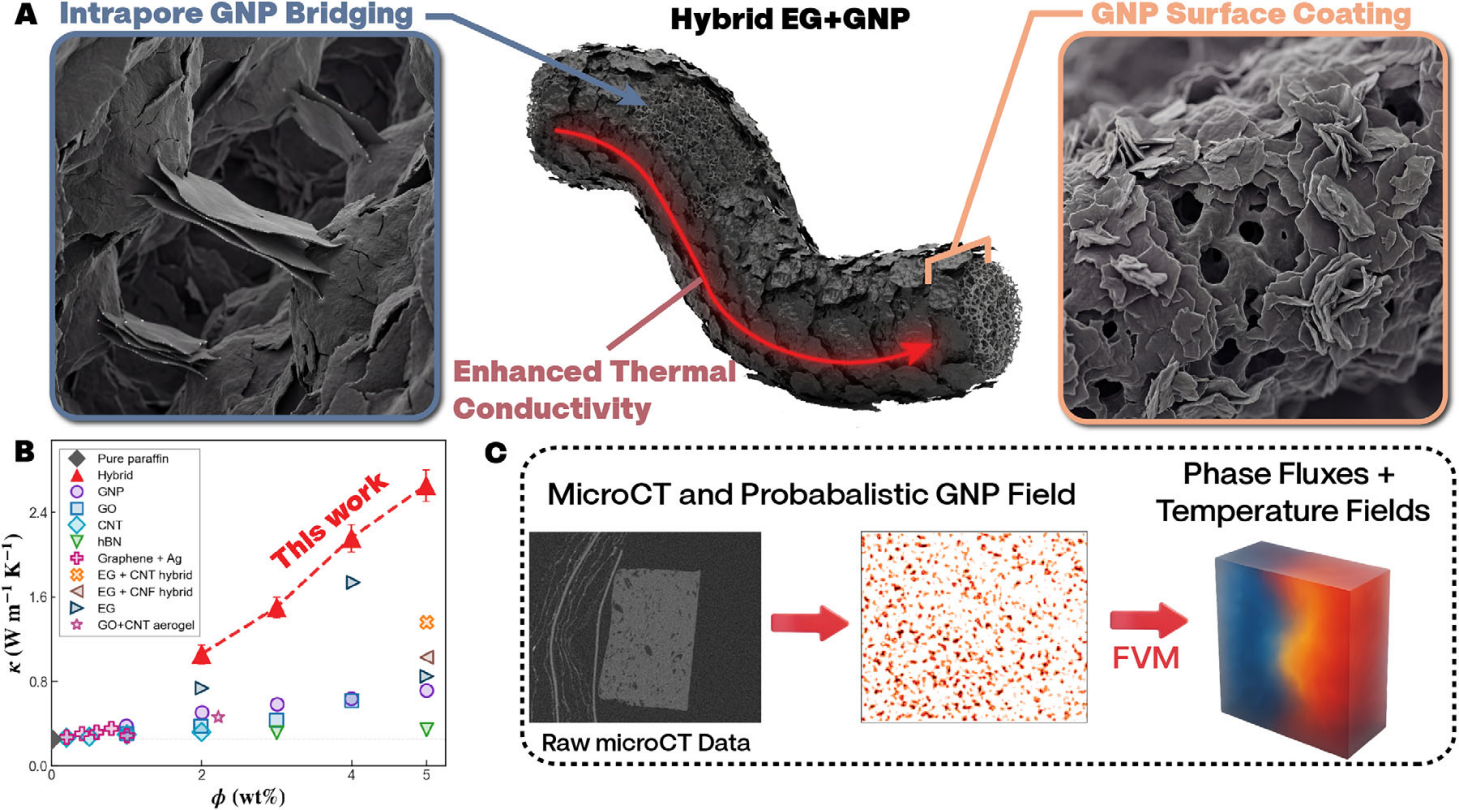
Thermal transport and synergistic hybrid‐filler mechanisms in paraffin/EG+GNP composites and a microCT‐informed modeling workflow. (A) Artistic renderings illustrating intrapore GNP bridging and GNP surface coating that enhance conduction through the EG backbone and restructure heat‐flow pathways in the hybrid architecture. (B) Room temperature thermal conductivity (κ) as a function of total filler loading (ϕ, wt%) for hybrids, shown alongside literature results for comparison [26, 39, 40, 41, 42, 43, 44]. (C) Three‐dimensional microstructure‐informed transport framework that couples microCT‐derived architectures with a resolution‐aware soft GNP enrichment field and finite‐volume modeling (FVM) to compute an effective thermal conductivity ($\kappa_{\mathrm{eff}}$); phase‐resolved temperature and heat‐flux fields enable quantitative attribution of the measured enhancement to specific transport pathways.

Here, we investigate paraffin‐based phase‐change composites filled with EG and GNPs, two graphitic additives that span complementary length scales. We find that EG–GNP hybrids deliver large thermal‐conductivity gains at low total loading. At 5 wt%, hybrids raise κ from ≈0.25±0.01 to $\approx 2.7\pm 0.2\;W\;m^{-1}\;K^{-1}$, exceeding EG‐only and GNP‐only baselines while preserving the melting window and latent‐heat capacity. This performance cannot be explained by filler volume fraction or percolation geometry alone. To identify the controlling transport features, we develop a novel, resolution‐aware 3D microstructure‐to‐transport framework that unifies both length scales within a single model. The approach combines microCT reconstructions that explicitly resolve the spanning EG backbone with a continuous, sub‐voxel soft‐GNP enrichment field P(r), an intensity‐derived voxel‐wise measure of local GNP enrichment where individual platelets cannot be explicitly resolved, constrained to the nominal loading and calibrated against GNP‐only measurements. The resulting framework reproduces the measured hybrid trends and suggests a previously unrecognized transport mechanism whereby GNPs reinforce the EG worms, improving intraworm connectivity, mitigating transport bottlenecks, and enhancing load sharing, as illustrated in Figure 1. More broadly, this work establishes a general framework for identifying and engineering synergistic transport pathways in hybrid composite materials, providing design principles for thermal management systems and multifunctional energy materials.

## 2 Results

### 2.1 Phase and Microstructure Characterization

**FIGURE 2:**
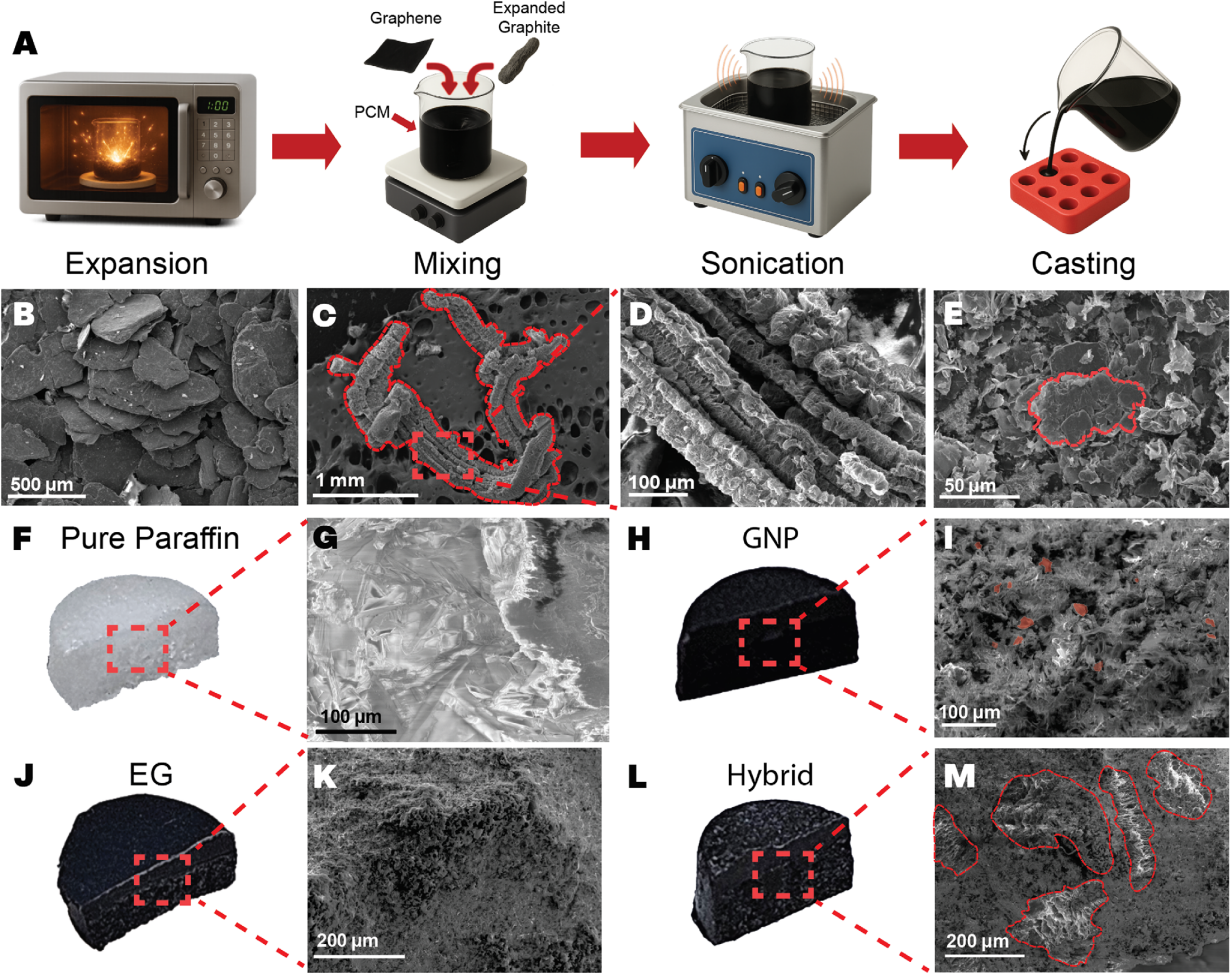
Fabrication and fracture‐surface microstructures of EG–GNP composites (A) Fabrication workflow schematic. (B) SEM of the expandable‐graphite precursor. (C) Expanded‐graphite (EG) worm (red outline). (D) Porous lamellar interior of an EG worm (higher magnification). (E) Graphene nanoplatelets (GNPs); representative flake/agglomerate outlined in red. (F–M) Representative pellets and corresponding fracture surfaces (all composites (H–M) at 4 wt% total filler). (F,G) Paraffin control: (F) pellet; (G) fracture surface. (H,I) GNP (4 wt%): (H) pellet; (I) fracture‐surface SEM with GNP‐rich regions highlighted. (J,K) EG (4 wt%): (J) pellet; (K) fracture‐surface SEM showing the EG network. (L,M) Hybrid EG+GNP (4 wt% total): (L) pellet; (M) fracture‐surface SEM with EG worms outlined in red.

Paraffin composites were fabricated by melt mixing and bath sonication followed by casting into pellets (Figure 2A). We use the labels EGx and GNPx for single‐filler samples and Hx for 1:1 EG+GNP hybrids, where x is the total filler wt% (Table 1). Samples were characterized using X‐ray diffraction (XRD), Raman spectroscopy, and Fourier‐transform infrared (FTIR) spectroscopy. Representative spectra for paraffin, EG, GNP, and the composites are provided in Figure S1. These measurements confirm the expected crystalline phase of the paraffin, the graphitic character of the carbon fillers, and the absence of new chemical species after processing. Composite architecture was then examined across length scales from local morphology to mesoscale network connectivity.

**TABLE 1:** Composite sample nomenclature and respective compositions.

Weight%	Graphene nanoplatelets	EG	Hybrid (EG:GNP 1:1)
1 wt%	GNP1	EG1	—
2 wt%	GNP2	EG2	H2 (EG1 + GNP1)
3 wt%	GNP3	EG3	H3 (EG1.5 + GNP1.5)
4 wt%	GNP4	EG4	H4 (EG2 + GNP2)
5 wt%	GNP5	EG5	H5 (EG2.5 + GNP2.5)

Scanning electron microscopy (SEM) images of the fillers can be seen in Figure 2B–E, and fracture surfaces of each sample type can be seen in Figure 2G,I,K,M. In Figure 2G, the pure paraffin fracture surface is mostly smooth and amorphous, with no obvious voids or second phase. Occasional shallow facets suggest small crystalline domains, but no organized texture is resolved. By contrast, the GNP composite (Figure 2I) exhibits a rough, corrugated morphology. Local steps and pits are prevalent, with exposed platelets highlighted in red. The expanded‐graphite composite (Figure 2K) shows a rough, pitted surface with undercut ledges, consistent with a porous expanded‐graphite network intersecting the fracture surface. However, a continuous worm cross‐section is not directly resolved, and the existence of a large contiguous EG network cannot be confirmed from this image alone. Finally, the hybrid composite (Figure 2M) clearly shows several intact, porous EG worms homogeneously dispersed across the field. The surrounding fracture surface is less openly porous than in the EG‐only composite. Small platelet‐like features are only ambiguously resolved at this magnification, so the presence of exposed GNP flakes cannot be confirmed from secondary‐electron contrast alone. Notably, the central worm is partially covered by matrix. The covered portion closely resembles the rough, pitted texture seen in the EG‐only fracture (Figure 2K). This similarity indicates that the EG‐only specimen requires an independent check of worm continuity. The matrix can cover a worm cross‐section and make it resemble the EG fracture appearance. Thus, the SEM image of the EG‐only sample alone does not establish whether a contiguous EG network survived processing.

**FIGURE 3:**
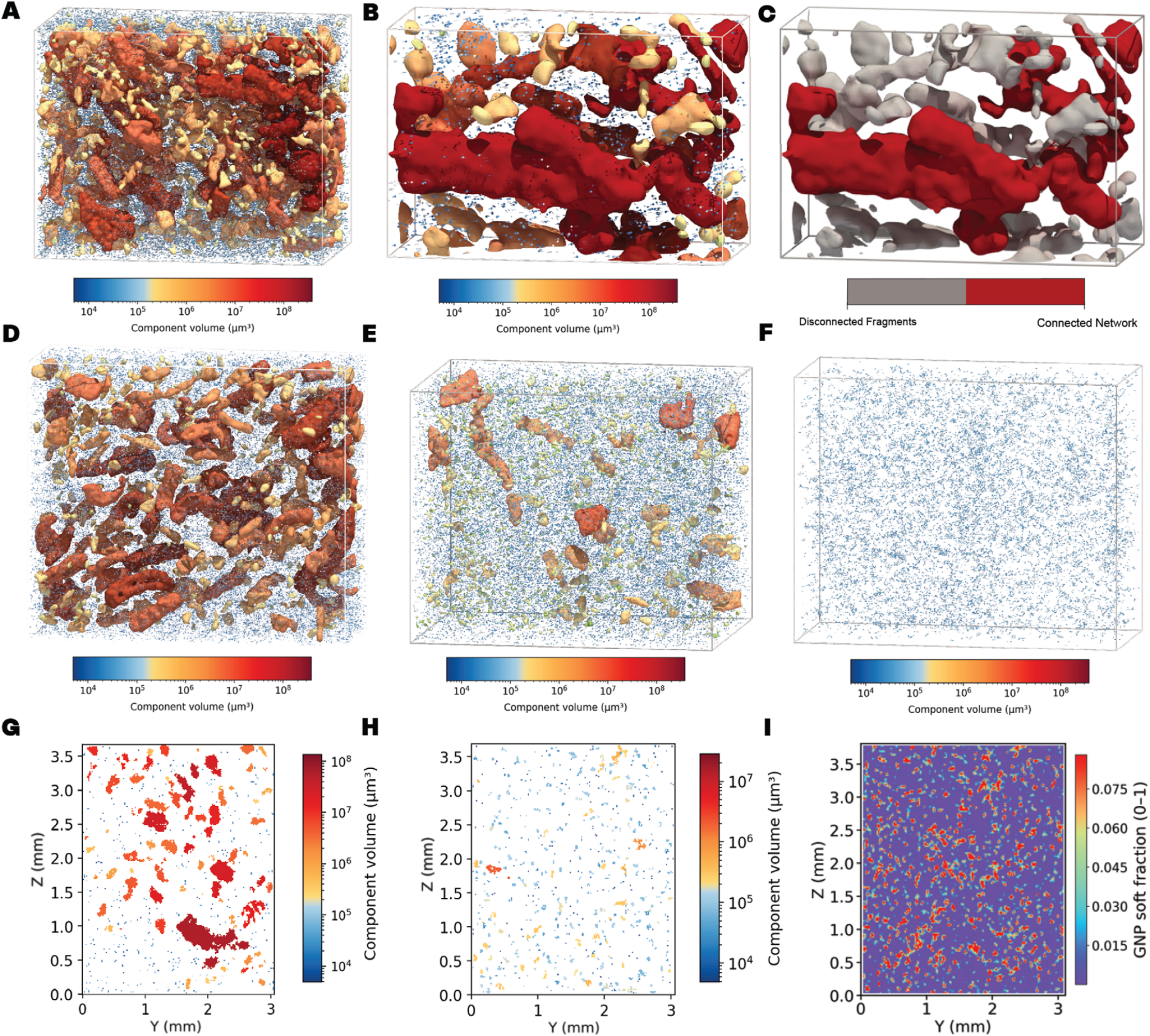
MicroCT 3D microstructure reconstructions and 2D slices along the YZ plane. EG worms are rendered as surface meshes colored by the total volume of each component (log scale, $10^{4}$–$10^{8}$ μm 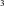). GNP‐containing voxels are rendered as subsampled disk markers for visibility. Segmented solids were smoothed, contoured, and ray traced for visualization. (A–C) Hybrid H5 at 5 wt% total loading. (A) Full RVE colored by worm component volume. (B) Zoom of the top‐right region of (A). (C) Extracted percolating EG network with non‐percolating fragments shown in gray. (D–F) 4 wt% total loading. (D) Hybrid H4. (E) EG‐only EG4. (F) GNP‐only GNP4. (G,H) Mid‐plane sagittal (Y–Z) slices extracted from (D) and (E), respectively, showing connected‐component volume maps of the segmented solids. (I) Mid‐plane sagittal (Y–Z) slice of the soft GNP field P(r) generated from GNP4 in (F), where color indicates the local GNP soft fraction on a 0–1 scale.

MicroCT was used to quantify bulk 3D architecture for each composition using representative volume elements (RVEs). For each sample, a single RVE was defined as the largest axis‐aligned rectangular prism inscribable within the cylindrical sample bulk, spanning the full sample height along the applied thermal‐gradient direction and excluding only the curved edge regions. Accordingly, the analyzed domain occupied most of the sample volume and differed from the full sample only at the circular perimeter. These RVEs were segmented into the EG phase, pore, and GNP‐containing phases to compare connectivity and dispersion across samples while avoiding edge and reconstruction artifacts (Figure S2). Figure 3 summarizes representative microCT‐based visualizations and slice views of the reconstructed microstructures, where each segmented structure is colored by the total volume of its parent connected component. The 3D reconstruction of H5 (Figure 3A–C) shows a high worm survival density and a large dominant connected component, forming a strongly connected network of elongated EG worms that spans the RVE, with GNP enrichment distributed throughout the surrounding matrix (Figure 3A). Voxels identified as GNP‐containing are rendered as subsampled disk glyphs for visual clarity, with the flat disk geometry chosen to evoke the platelet morphology of the GNP. The worm phase is spatially non‐uniform, consisting of a dominant connected backbone coexisting with isolated worm fragments and matrix‐rich gaps, as seen in the zoomed view in Figure 3B, confirming that a robust EG network survives the mixing and sonication steps when GNP is present. In Figure 3C, isolating the percolating component and coloring the reconstructed EG phase to distinguish the connected network from disconnected fragments shows that long‐range connectivity is carried primarily by the largest worms, whereas non‐percolating remnants tend to be smaller and more fragmented.

Relative to H4 at 4 wt% (Figure 3D), H5 shows a higher apparent worm survival density and a larger dominant connected component. Compared with the hybrids, EG4 (Figure 3E) shows no comparable spanning worm network. Instead, the low‐intensity phase consists predominantly of smaller inclusions and short fragments, suggesting that most worms were exfoliated into fine flakes during processing. The mid‐plane slices at 4 wt% mirror this contrast. Each intersection is colored by its parent 3D connected‐component volume on a log scale in $\mu m^{3}$. The slices therefore give a cross‐sectional readout of network dominance. H4 (Figure 3G) shows repeated warm‐colored intersections from the largest components that approach $10^{8}$ $\mu m^{3}$. This indicates that a few connected structures contribute disproportionately to the section. In EG4 (Figure 3H), warm colors are rare and the scale tops out near $10^{7}$ $\mu m^{3}$. Most intersections appear as cool‐colored speckles. This is consistent with connectivity being partitioned among many small disconnected objects.

Because worms and pores have similar attenuation, they cannot be cleanly separated by intensity alone. In hybrid samples, the elongated geometry and network connectivity allow us to distinguish worms from pores. However, in the EG‐only samples, the larger low‐intensity objects are largely compact and approximately spherical, making an automated separation between worm remnants and pores unreliable. To enable a conservative volume‐fraction comparison, we therefore treat all connected low‐intensity components in the EG‐only samples as a combined EG+pores phase, and compare this to the EG mask in the hybrid samples. A comparison of their volume fractions is reported in Table S1.

In hybrid composites, a key challenge in using 3D imaging as the basis for thermal‐transport simulation is that the imaging resolution is often coarser than the relevant filler length scales. Here, GNP platelets are sub‐voxel at roughly 17 μ m microCT resolution, so individual platelets cannot be explicitly resolved, and solid‐phase voxels generally encode mixtures of paraffin and randomly oriented GNPs. In this regime, hard‐threshold segmentation is ill‐posed and can bias both the inferred GNP fraction and its spatial placement. We therefore introduce an intensity‐derived soft‐GNP enrichment field P(r) based on voxel‐wise Gaussian‐mixture responsibilities, which provides a resolution‐aware description of local GNP enrichment that can be propagated into the subsequent transport model. On non‐pore, non‐EG voxels we define

(1) $$P\left(r\right)=\frac{R_{\mathrm{GNP}}\left(r\right)}{R_{\mathrm{mat}}\left(r\right)+R_{\mathrm{GNP}}\left(r\right)},\;0\leq P\left(r\right)\leq 1,$$

where $R_{\mathrm{GNP}}\left(r\right)$ and $R_{\mathrm{mat}}\left(r\right)$ are the voxel‐wise responsibilities for the GNP and paraffin‐matrix components, respectively.

Here, P(r) quantifies the local degree of GNP enrichment within the solid phase relative to paraffin; more details on its construction can be found in the Supporting Information. The resulting 3D rendering and orthogonal soft‐GNP enrichment map in Figure 3I show that high‐posterior voxels occur as small, spatially coherent clusters that decorate the matrix. Across all three viewing planes, these clusters are distributed homogeneously throughout the RVE, with no evidence of large‐scale gradients or a continuous GNP network. Consistent with the low overall GNP loading and sub‐voxel mixing, the soft GNP field values remain well below unity, indicating modest local enrichment rather than pure GNP domains. Together with the intensity separation and quantization analyses in the Supporting Information (Figures S3– S5), which show that matrix and GNP populations are well resolved, these maps demonstrate that the soft GNP field provides a physically meaningful, statistically homogeneous description of GNP dispersion.

### 2.2 Filler Evolution Characterization

**FIGURE 4:**
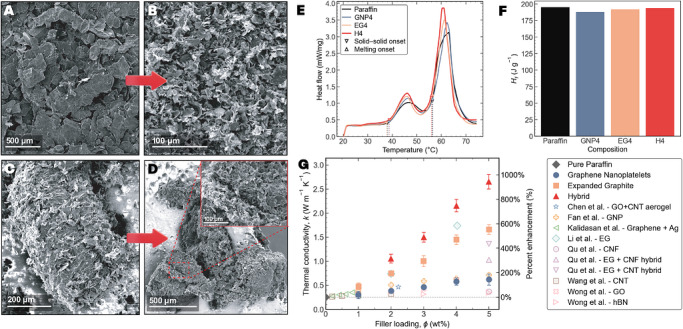
Microstructural evolution of EG and EG–GNP hybrid fillers during dispersion and thermal characterization of paraffin composites (A) EG after mechanical mixing. (B) EG after sonication for 60 min. (C) EG+GNP hybrid after mechanical mixing. (D) EG+GNP hybrid after sonication for 60 min. The dashed box in (D) indicates the inset region, which highlights platelet coverage on the worm surface. (E) Differential scanning calorimetry (DSC) heating curves for neat paraffin and the 4 wt% composites GNP4, EG4, and the hybrid H4, with solid–solid transition and melting onsets indicated. (F) Latent heat of fusion $H_{f}$ extracted from the DSC curves for each 4 wt% composition. (G) Room temperature thermal conductivity (κ) as a function of total filler loading ϕ (wt%), with percent enhancement relative to neat paraffin on the right axis. Literature values provided for comparison: Chen et al. [39] ‐ graphene oxide (GO) + carbon nanotube (CNT) aerogel (2:1); Fan et al. [40] ‐ GNPs; Kalidasan et al. [41] ‐ graphene‐Ag hybrid; Li et al. [42] ‐ EG; Qu et al. [26] – carbon nanofiber (CNF), EG+CNF hybrid, and EG+CNT hybrid in paraffin/high‐density polyethylene (HDPE); Wang et al. [43] ‐ multi‐walled carbon nanotubes (MWCNTs); and Wong et al. [44] ‐ GO and hexagonal boron nitride (hBN).

To establish how the mixing and sonication treatment modifies EG integrity and hybrid architecture, we performed SEM on fillers isolated after each step (Figure 4A–D). Performing SEM directly on fillers embedded in paraffin is not feasible because, upon solidification, the matrix obscures the filler surfaces and masks the fine morphological features of interest. We therefore reproduced the procedure used for composite processing in ethanol at room temperature and isolated the fillers after each step for imaging. Ethanol is modestly less viscous than the molten paraffin used during composite processing, but both liquids have comparably low viscosities under the relevant conditions. The observed trends are therefore interpreted qualitatively, and conservatively, as representative of the paraffin‐processing response [45, 46, 47]. Figure 4A–D reveals a clear processing‐dependent divergence between EG‐only and hybrid fillers. In the EG‐only case, the mechanically mixed material retains large worm fragments (Figure 4A), whereas sonication drives pronounced exfoliation into smaller, thin flakes resembling GNP‐like platelets (Figure 4B), consistent with established liquid‐phase ultrasonic exfoliation of graphite/EG into graphene nanoplatelets [48, 49, 50]. By contrast, the hybrid fillers preserve a coherent worm backbone after both mixing and sonication (Figure 4C,D), and the sonicated hybrid shows apparent platelet coverage on portions of the surviving worm surfaces (Figure 4D). The processing‐scale SEM results suggest that GNPs may help reinforce the EG backbone against mixing‐ and sonication‐driven breakup and may contribute local surface coverage during processing, consistent with the larger contiguous EG‐like phase and spanning EG network resolved by microCT in the hybrid composites.

### 2.3 Melting Point and Latent Heat

Differential scanning calorimetry (DSC) heating curves for neat paraffin and the 4 wt% composites (GNP4, EG4, and H4) are shown in Figure 4E. In every case, the DSC response exhibits two distinct transitions, namely a lower‐temperature solid–solid transition or pre‐melting transition and a higher‐temperature melting peak. Across all compositions, the first peak occurs at 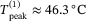 with an onset near 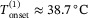. The main melting peak lies in a narrow range of $T_{\text{peak}}^{\left(2\right)}=61.2$–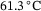 with an onset of $T_{\text{onset}}^{\left(2\right)}=55.9$–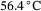, indicating that the addition of GNP, EG, or hybrid filler at these low loadings does not measurably shift the melting behavior relative to neat paraffin.

The corresponding latent heats of fusion, $H_{f}$, extracted from the area under the main melting peak on heating, are plotted in Figure 4F. Within experimental scatter, $H_{f}$ shows no significant decrease across filler type or loading, demonstrating that the thermal energy storage capacity of the PCM is largely preserved at these filler fractions. The specific heat capacities ($C_{p}$) obtained by the $C_{p}$‐ratio method for pure paraffin and EG2 are shown in Figure S6. Both compositions display similar baseline heat capacities away from the transition and a pronounced peak in $C_{p}$ across the melting interval, with only modest reductions in the apparent peak height upon filler addition. Together, these measurements confirm that the fillers leave both the phase‐change temperature window and the latent heat of fusion essentially unchanged while introducing only minor modifications to the sensible heat capacity.

### 2.4 Thermal Conductivity Results

The κ of neat paraffin and the GNP, EG, and hybrid composites, benchmarked against representative paraffin‐based systems using comparable processing parameters and loading fractions from the literature [26, 39, 40, 41, 42, 43, 44], is summarized in Figure 4G. Key parameters for each literature dataset are compiled in Table 2. Neat paraffin exhibits $\kappa \approx 0.25\pm 0.01\;W\;m^{-1}\;K^{-1}$. Across all composite samples, κ increases approximately linearly with loading ϕ (wt%), but the three series differ strongly in slope. The GNP composites show modest gains and reach a maximum enhancement slightly above 150% at ϕ=5 wt%. The EG composites increase much more steeply, with the 5 wt% EG sample approaching a 600% enhancement relative to neat paraffin. The hybrids increase most strongly across the full range and achieve nearly a 1000% enhancement ($\approx 2.7\pm 0.2\;W\;m^{-1}\;K^{-1}$) at 5 wt% total filler. At this loading, the hybrid gain is roughly twice that of the EG‐only composite and more than five times that of the GNP‐only composite. To assess the stability of the hybrid transport network under repeated phase transitions, the H2 sample was subjected to two successive melting–resolidification cycles and remeasured after each cycle. The thermal conductivity remained consistent within experimental uncertainty after both cycles (Figure S7), indicating that the effective heat‐conducting hybrid structure was retained over the range tested.

**TABLE 2:** Summary of literature parameters for composites plotted in Figure 4G.

Study	Filler type	Loading	Processing method	Matrix	κ (W $m^{-1}$ $K^{-1}$)
[26]	CNF, EG+CNF hybrid, EG+CNT hybrid	5 wt.%	Melt blending + repress molding (HDPE shape stabilization)	Paraffin‐HDPE	0.37–1.36
[39]	GO+CNT aerogel (2:1)	2.2 wt.%	Preformed hybrid aerogel scaffold + vacuum impregnation	Paraffin wax	0.46
[40]	GNP	1–5 wt.%	Melt mixing + sonication	Paraffin wax	0.26–0.70
[41]	Graphene‐Ag hybrid	0.2–1.0 wt.%	Hot‐plate melting + bath sonication	Organic paraffin RT50	0.2–0.33
[42]	EG	2, 4 wt.%	Low‐speed mixing + hot‐plate infiltration + compression molding	Paraffin wax	0.74–1.74
[43]	MWCNTs	0.2–2.0 wt.%	Ball milling + melt mixing/sonication	Paraffin wax	0.24–0.32
[44]	GO, hBN	1–5 wt.% (GO), 1–5 wt.% (hBN)	Solution blending + sonication	Paraffin wax	0.24–0.84 (GO), 0.27–0.35 (hBN)
**This work**	**EG+GNP hybrid (1:1)**	**2–5 wt.%**	**Melt mixing, bath sonication, casting**	**Paraffin wax**	**1.1–2.7**

With the literature data superimposed in Figure 4G, the single‐filler series aligns with established trends. The GNP dataset closely matches the low‐loading behavior reported by Fan et al. and is consistent with related paraffin–GNP/CNT studies [40, 43]. The EG‐only series tracks or modestly exceeds the conductivities reported for EG foams by Li et al. [42]. At comparable low loadings, the hybrids outperform similar paraffin composites reported to date while avoiding processing‐intensive, highly architected filler networks, supporting a more scalable route to large κ enhancement [39, 41, 44]. Many reported systems reach comparable κ gains only at substantially higher filler fractions, where reduced paraffin volume is expected to penalize latent‐heat storage and architectural complexity increases materials and processing cost [51, 52, 53]. In this context, achieving large enhancements at ϕ≤5 wt% helps reduce the trade‐offs among κ, latent‐heat capacity, and filler loading. However, κ(ϕ) alone does not reveal which phases carry the added heat or how the fillers interact, motivating our combination of the microCT reconstructions in Figure 3 with an image‐based finite‐volume model (FVM) to solve the steady‐state heat equation and resolve the underlying transport mechanisms.

### 2.5 3D Microstructure Based Modeling Framework

**FIGURE 5:**
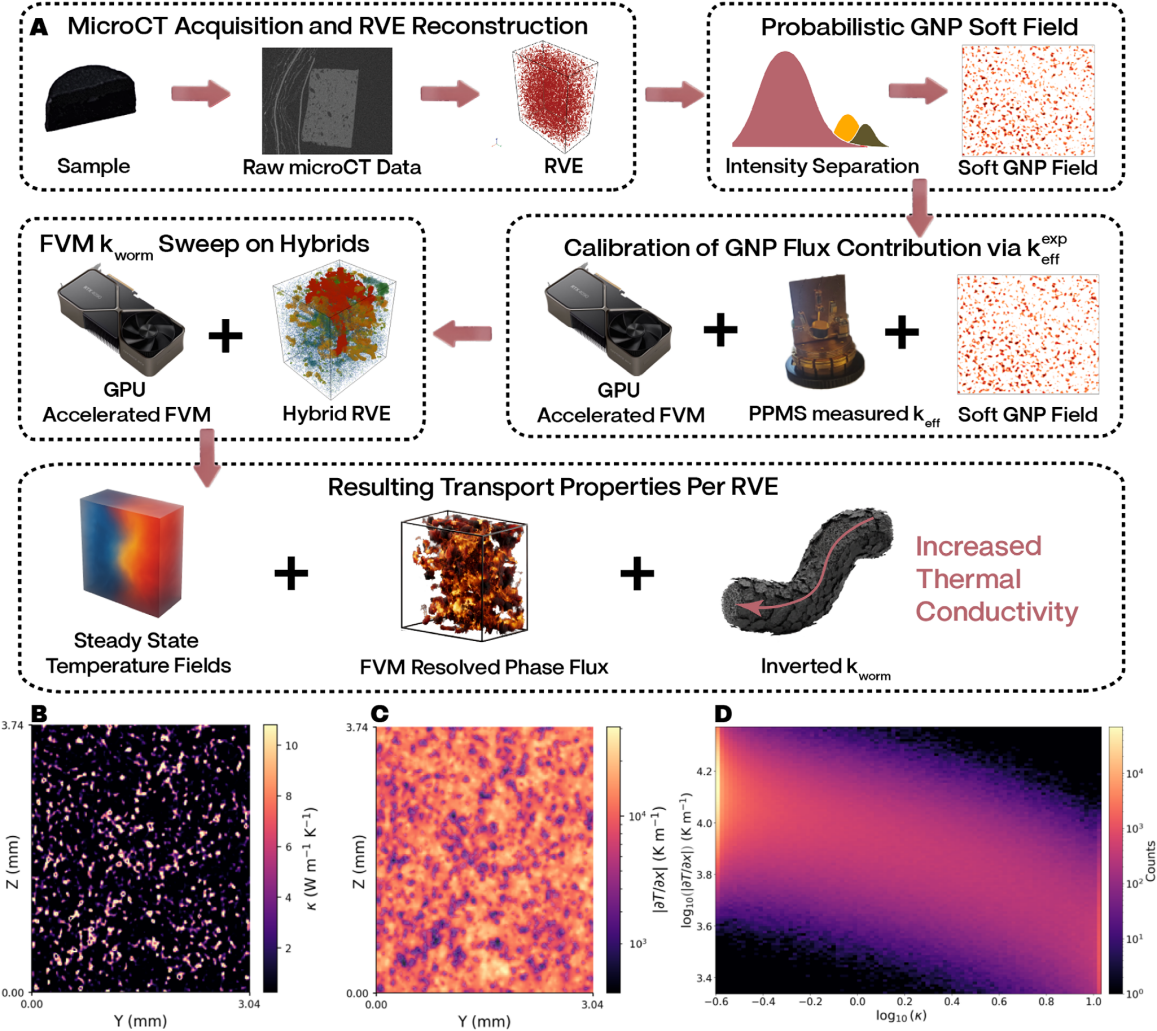
Image‐based modeling workflow and GNP calibration thermal transport diagnostics. (A) Schematic of the modeling procedure. MicroCT RVEs are converted to phase fields for the EG phase and pores, with sub‐voxel GNP enrichment represented by a soft enrichment field. These fields are mapped to a voxel‐wise conductivity tensor, calibrated on GNP‐only RVEs against measured κ, and applied to hybrids to infer $\kappa_{\mathrm{worm}}$. Outputs are steady‐state temperature and phase‐resolved flux fields. (B)–(D) Representative YZ slices of the GNP4 RVE showing (B) the voxel‐wise in‐plane conductivity κ obtained from P(r) using the fitted calibration parameters, (C) the converged temperature‐gradient magnitude |∂T/∂x|, and (D) a voxel‐wise 2D histogram of |∂T/∂x| versus κ.

To interpret the measured κ of the hybrid composites, we developed a 3D image‐based transport framework that directly integrates microCT‐derived microstructures. A schematic of this workflow is shown in Figure 5A. The reconstructed EG network is combined with the soft‐GNP enrichment field P(r)∈[0,1] defined above (Equation 1) to represent matrix‐phase platelet dispersion without hard segmentation. Importantly, the model does not explicitly resolve a distinct GNP shell at the EG surface; rather, P(r) captures sub‐voxel GNP enrichment only in the surrounding matrix. These microstructural fields define the voxel‐wise conductivity map κ(r) used as input to our finite‐volume calculations on the native grid.

We generated a voxel‐level conductivity map κ(r) from these fields and implemented a graphics processing unit (GPU)‐accelerated FVM to compute the effective thermal conductivity ($\kappa_{\mathrm{eff}}$) on the native grid. Solving the steady‐state heat equation, ∇·κ(r)∇T=0, directly on the microCT‐derived architecture eliminates the need for idealized geometric assumptions. To replicate experimental conditions, we imposed Dirichlet boundary conditions along the applied thermal‐gradient direction, with transverse periodic constraints (Figure S8). The solver was rigorously benchmarked against closed‐form reference problems and the Cu/diamond system of Chang et al. [54], recovering their reported κ to within 0.2%, highlighted in Figures S17 and S18.

Finally, we established a two‐step calibration protocol to anchor model parameters to experiment. We first isolated the constitutive response of the paraffin+GNP matrix using the GNP‐only series, where P(r) smoothly modulates local transport. These parameters were then held fixed for hybrids. Agreement with measured κ was achieved by inverting a single effective worm‐phase conductivity, $\kappa_{\mathrm{worm}}$, for the reconstructed EG network. The inferred $\kappa_{\mathrm{worm}}$ should be interpreted as an effective worm‐phase parameter that subsumes both transport through the EG ligament framework and worm–matrix interfacial effects. Together, the loading‐dependent $\kappa_{\mathrm{worm}}$ inversion results, ex situ processing‐scale SEM observations, and phase‐resolved flux maps that identify critical transport pathways support the proposed mechanism of dual‐filler synergy. Full definitions, numerical implementation details, and validation benchmarks are provided in the Supporting Information.

#### 2.5.1 GNP Calibration

Calibration of the soft‐GNP mixture model was performed using the 2 and 4 wt% GNP composites. The calibration adjusts a small set of matrix‐response parameters that map the microCT‐derived soft‐GNP field into voxel‐level conductivity tensors κ(r). Here, $\kappa_{∥,\mathrm{nom}}$ sets the nominal in‐plane conductivity scale of the GNP‐rich response. The through‐plane base scale is set by $\kappa_{⊥,\mathrm{base}}$, and γ controls how strongly the platelet‐normal channel is throttled at elevated local GNP loading. The spatial mean of the bounded local connectivity weight w(r) is prescribed by $w_{\mathrm{target}}$. The parameter β controls the steepness of the sigmoid mapping from the soft field to w(r), setting how abruptly regions transition from matrix‐like to GNP‐like response. The per‐sample scale factor $s_{\mathrm{soft}}$ maps the soft field into an effective local loading. For each trial parameter set, voxel‐level κ tensors were constructed from the microCT‐derived soft‐GNP fields, and the FVM was run on the corresponding RVEs to obtain simulated effective conductivities, $\kappa_{\mathrm{eff},x}^{\mathrm{sim}}$, where the subscript x denotes the effective conductivity along the imposed temperature‐gradient direction. An unweighted least‐squares misfit was then evaluated against the measured conductivities, $\kappa_{\mathrm{eff}}^{\exp}$, at these two loadings. A stochastic search over a physically constrained parameter space was used to minimize this misfit. See Supporting Information for the constitutive definitions and details of the search strategy.

Table 3 summarizes the best‐fit parameter set obtained from the joint calibration to the 2 and 4 wt% GNP‐only composites. The best‐fit parameter set matches the measured conductivities of both GNP‐only composites within the experimental uncertainty. The 2 wt% case gives $\kappa_{\mathrm{eff},x}^{\mathrm{sim}}=0.3851\;W\;m^{-1}\;K^{-1}$ versus $\kappa_{\mathrm{eff}}^{\exp}=0.385\;W\;m^{-1}\;K^{-1}$, and the 4 wt% case gives $\kappa_{\mathrm{eff},x}^{\mathrm{sim}}=0.5795\;W\;m^{-1}\;K^{-1}$ versus $0.580\;W\;m^{-1}\;K^{-1}$. In particular, the fitted nominal in‐plane κ of the GNP‐rich phase is $\kappa_{∥,\mathrm{nom}}\approx 193\;W\;m^{-1}\;K^{-1}$. The through‐plane base κ is $\kappa_{⊥,\mathrm{base}}\approx 4.7\;W\;m^{-1}\;K^{-1}$. As an additional consistency check, we used a coated‐platelet effective‐medium theory model to verify that the calibrated $\kappa_{∥,\mathrm{nom}}$ is compatible with plausible graphene–paraffin interfacial thermal resistances (Figure S19) [55, 56, 57]. The crowding parameter γ≈6.9 indicates strong throttling of the through‐plane channel at elevated local GNP loading. The mean connectivity weight inferred from the soft‐GNP fields increases from $w_{\mathrm{target}}=0.26$ at 2 wt% to $w_{\mathrm{target}}=0.50$ at 4 wt%, consistent with more extensive GNP connectivity at higher loading, while the per‐sample soft‐field scales $s_{\mathrm{soft}}$ remain close to unity.

**TABLE 3:** Best‐fit parameters from the joint calibration of the image‐based finite‐volume model to the 2 and 4 wt% GNP composites. $\kappa_{∥,\mathrm{nom}}$, $\kappa_{⊥,\mathrm{base}}$, γ, and β are shared across both compositions; $w_{\mathrm{target}}$ and $s_{\mathrm{soft}}$ are per‐sample parameters that map the soft GNP field to an effective local GNP loading. The final columns compare the fitted model prediction $\kappa_{\mathrm{eff},x}^{\mathrm{sim}}$ with the measured value $\kappa_{\mathrm{eff}}^{\exp}$.

Sample	$\kappa_{∥,\mathrm{nom}}$	$\kappa_{⊥,\mathrm{base}}$	γ	β	$w_{\mathrm{target}}$	$s_{\mathrm{soft}}$	$\kappa_{\mathrm{eff},x}^{\mathrm{sim}}$	$\kappa_{\mathrm{eff}}^{\exp}$
	(W $m^{-1}$ $K^{-1}$)	(W $m^{-1}$ $K^{-1}$)					(W $m^{-1}$ $K^{-1}$)	(W $m^{-1}$ $K^{-1}$)
2 wt%	192.7	4.75	6.93	2.0	0.26	1.20	0.3851	0.3850
4 wt%	192.7	4.75	6.93	2.0	0.50	1.40	0.5795	0.5800

Figure 5B–D reports representative voxel‐wise transport fields for the GNP4 RVE obtained with the fitted calibration parameters. The voxel‐wise κ(r) field in Figure 5B exhibits a continuous spectrum of conductivities consistent with its construction from the soft field P(r). Most voxels remain near the matrix baseline ($∼\;0.25\;W\;m^{-1}\;K^{-1}$), while GNP‐enriched regions populate a sparse upper tail reaching $∼\;10\;W\;m^{-1}\;K^{-1}$, highlighting strong spatial heterogeneity in the local conductivity field. These upper‐tail magnitudes are in line with prior experimental graphene–paraffin composite κ measurements [58], providing a benchmark for the inferred local κ values. In the corresponding steady conduction solution shown in Figure 5C, the temperature‐gradient field shows the expected high‐contrast behavior. High‐κ regions tend to be nearly isothermal, while the temperature drop and larger |∂T/∂x| concentrate in the more resistive matrix constrictions between them, consistent with field‐concentration effects in the “perfect conductor inclusion” limit [59]. This response is quantified in Figure 5D, where the voxel‐wise joint distribution shows an overall anti‐correlation between κ and |∂T/∂x|, supporting the interpretation that GNP‐rich regions primarily act to locally short‐circuit temperature drops, while the dominant gradient‐bearing bottlenecks reside in the lower‐κ matrix.

#### 2.5.2 $\kappa_{worm}$ Extraction

**FIGURE 6:**
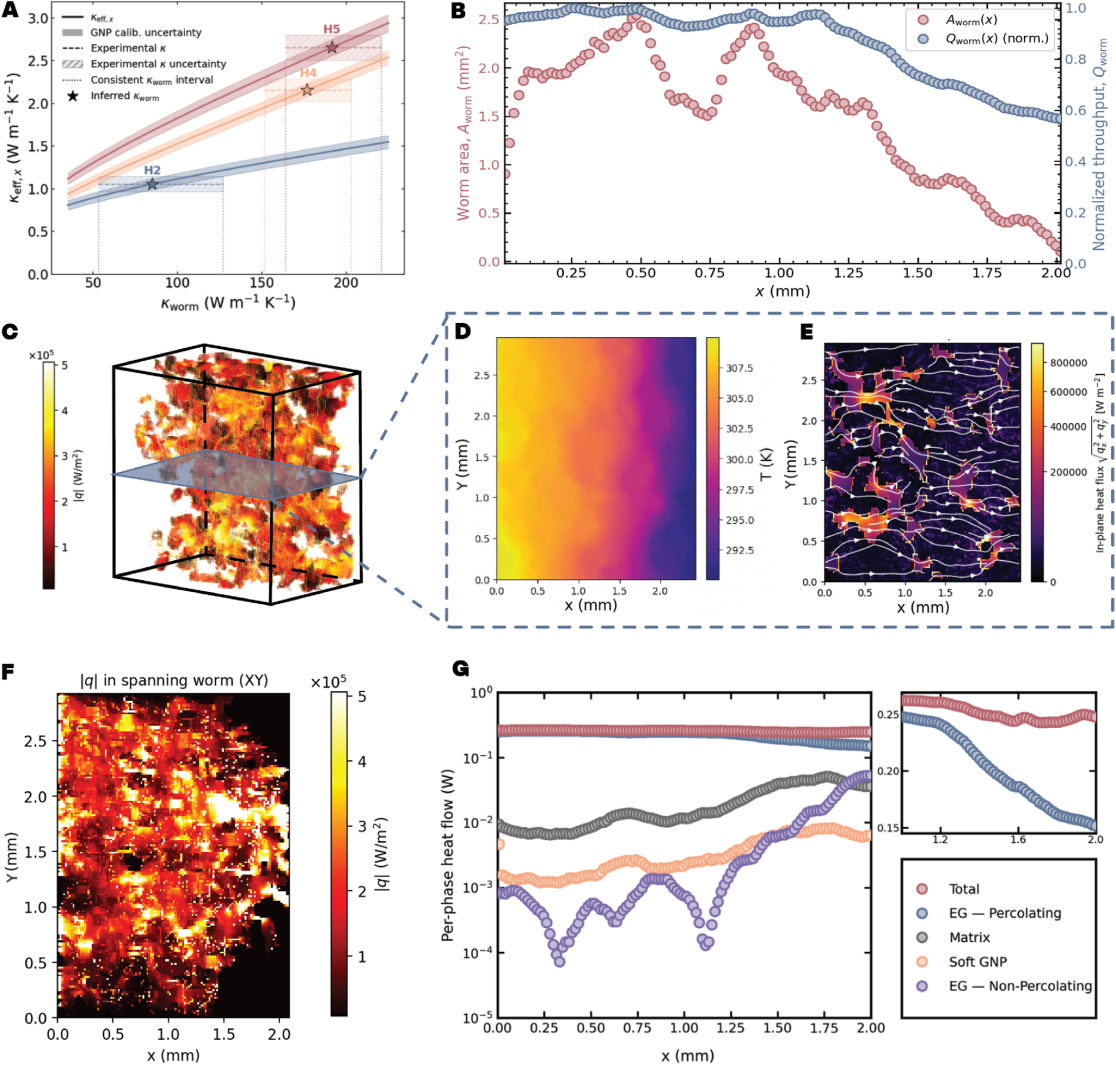
Image‐based finite‐volume modeling of heat transport in hybrid EG–GNP composites. (A) $\kappa_{\mathrm{eff},x}^{\mathrm{sim}}\left(\kappa_{\mathrm{worm}}\right)$ for hybrid RVEs H2, H4, and H5. Solid shading spans the GNP calibration envelope; hatched bands mark experimental $\kappa_{\mathrm{eff},x}$ uncertainty, and their overlap defines the consistent $\kappa_{\mathrm{worm}}$ interval (dotted verticals, stars). (B) Cross‐sectional area of the spanning EG worm network (solid red) and normalized slice throughput $Q_{\mathrm{worm}}$ (dashed blue) versus position x. (C) 3D view of the percolating EG worm network in H5, colored by steady‐state heat‐flux magnitude |q|; the shaded plane indicates the slice used in (D)–(F). (D)–(F) XY slice of the H5 RVE showing (D) the temperature field, (E) in‐plane heat‐flux magnitude with streamlines seeded from voxels in the top 2% of $|q_{xy}|$, and (F) projection of the spanning EG worm network colored by $|q_{xy}|$. (G) Per‐slice decomposition of the plane‐integrated heat flow $Q_{\mathrm{phase}}\left(x\right)$ into individual phase contributions and their sum.

The GNP calibration fixes the paraffin–GNP response, leaving the worm‐phase conductivity $\kappa_{\mathrm{worm}}$ as the sole adjustable parameter in the hybrid RVEs. For each hybrid RVE, $\kappa_{\mathrm{worm}}$ was treated as the only adjustable parameter after fixing the paraffin–GNP response. The FVM was then run repeatedly over a range of trial $\kappa_{\mathrm{worm}}$ values (20–225 W $m^{-1}$ $K^{-1}$), and the corresponding simulated bulk conductivity along x, $\kappa_{\mathrm{eff},x}^{\mathrm{sim}}$, was recorded. Plotting these outputs against the trial $\kappa_{\mathrm{worm}}$ values gives the curves in Figure 6A. For all three hybrids, $\kappa_{\text{eff},x}$ increases approximately linearly with $\kappa_{\text{worm}}$. Because the GNP calibration is applied in orientation‐averaged form, any sub‐voxel changes to platelet orientation, dispersion, or connectivity introduced by the presence of EG are not separately identifiable and are instead absorbed into the inferred $\kappa_{\text{worm}}$. To quantify the sensitivity of the inversion to the GNP calibration, the joint fit was repeated at ±1σ perturbations of each GNP‐only target conductivity, and the resulting admissible parameter sets were propagated through the $\kappa_{\text{worm}}$ sweep (details in Supporting Information). The shading in Figure 6A shows the resulting envelope, vertical bands indicate the experimentally measured $\kappa_{\text{eff},x}$ and its uncertainty, and the corresponding intersections define $\kappa_{\text{worm}}$ intervals of 53.2–127.0 W $m^{-1}$ $K^{-1}$ for H2, 151.0–203.0 W $m^{-1}$ $K^{-1}$ for H4, and 164.0–220.9 W $m^{-1}$ $K^{-1}$ for H5. A pore‐toggle sensitivity analysis further showed that replacing pore voxels with paraffin matrix changed the modeled $\kappa_{\mathrm{eff}}$ by less than 0.4% for all three hybrid samples, indicating that differences in pore structure do not materially affect the observed hybrid conductivity enhancement (Figure S20).

As loading increases, the experimentally consistent $\kappa_{\mathrm{worm}}$ window shifts systematically upward. This shift tracks the microCT‐derived growth in contiguous worm volume fraction ($\phi_{\mathrm{EG}}=0.107$, 0.137, and 0.167 for H2, H4, and H5) and is consistent with the strong wt%‐dependent increase in hybrid $\kappa_{\mathrm{eff}}$ relative to the EG‐only and GNP‐only series. The simulated response $\kappa_{\mathrm{eff},x}^{\mathrm{sim}}\left(\kappa_{\mathrm{worm}}\right)$ already embeds the microCT‐resolved worm morphology, including continuity, tortuosity, and cross‐sectional variability. RVEs with more contiguous networks should therefore yield higher $\kappa_{\mathrm{eff},x}\left(\kappa_{\mathrm{worm}}\right)$ curves at identical $\kappa_{\mathrm{worm}}$. However, this connectivity boost does not collapse the hybrid data onto a single, composition‐independent $\kappa_{\mathrm{worm}}$. An explicit fixed‐$\kappa_{\mathrm{worm}}$ cross‐test further shows that no single composition‐independent $\kappa_{\mathrm{worm}}$ reproduces the full H2/H4/H5 trend when applied across all three hybrid RVEs (Figure S21). Matching the higher $\kappa_{\mathrm{eff}}^{\exp}$ at larger loadings still requires a larger effective $\kappa_{\mathrm{worm}}$. The inferred upward shift in $\kappa_{\mathrm{worm}}$ therefore reflects a residual, synergy‐driven enhancement of the intraworm effective κ beyond what increased $\phi_{\mathrm{EG}}$ and connectivity alone can supply. This behavior is consistent with sub‐voxel improvements within the worm phase, such as reinforcement, densification, or improved internal contacts.

#### 2.5.3 Thermal Transport Analysis

Interpreted with the microstructure data, the extracted $\kappa_{\mathrm{worm}}$ trends are consistent with a dual‐filler synergy in which GNPs stabilize and reinforce a spanning EG backbone. Ex situ SEM of fillers processed in ethanol (Figure 4A–D) suggests that, when GNPs are present, EG worms are better preserved during mixing and sonication and can develop dense platelet coverage on surviving worms, whereas EG‐only dispersions are largely broken into thinner platelets. MicroCT RVEs likewise show a larger contiguous EG phase and a more robust spanning backbone in H4 and H5 than in the EG‐only composite at comparable nominal loadings (Figure 3). The sharp increase in $\kappa_{\mathrm{worm}}$ from H2 to H4, followed by only a modest increase from H4 to H5 despite added hybrid volume, further suggests that GNP‐enabled reinforcement within the effective worm phase becomes sufficiently developed across critical transport pathways between H2 and H4, whereas at the highest loading additional GNP mainly thickens or locally patches already reinforced regions, leading to smaller incremental gains. The loading‐dependent increase in $\kappa_{\mathrm{worm}}$ is therefore consistent with GNP‐enabled reinforcement within the effective worm phase, potentially through surface coverage, intrapore infiltration or bridging, and improved internal EG contacts, beyond what $\phi_{\mathrm{EG}}$ and percolation alone predict. The fitted $\kappa_{\mathrm{worm}}$ magnitudes are also consistent with reported conductivities for paraffin‐filled EG worms. Py et al. report intraworm effective conductivities up to $∼70\;W\;m^{-1}\;K^{-1}$, depending on EG density and orientation [60], and Li et al. estimate an effective porous‐EG conductivity $\kappa_{p,\mathrm{num}}=132\;\phi_{\mathrm{EG}}+0.32$, where $\phi_{\mathrm{EG}}$ is the EG volume fraction within the EG worm, while emphasizing sensitivity to EG connectivity [42]. These comparisons support a plausible $\kappa_{\mathrm{worm}}$ window near 50 to $100\;W\;m^{-1}\;K^{-1}$ for non‐reinforced worms and attribute the systematic upward shift with loading to GNP‐enabled reinforcement of internal contacts within the spanning network.

This composition‐dependent increase in $\kappa_{\mathrm{worm}}$ helps rationalize the steep rise in hybrid $\kappa_{\mathrm{eff}}$ from H2 to H4. The modest additional increase in H5 despite added EG suggests diminishing returns at the highest loading. Table S2 gives reconstructed EG contents of 10.7%, 13.7%, and 16.7% for H2, H4, and H5, and in all three cases the EG worm network percolates across the RVE. The 3D reconstructions further show that going from H4 to H5 increases worm survival density, but much of the added material appears as disconnected islands separated by matrix‐rich gaps, while the percolating backbone is dominated by the largest worms (Figure 3A–C). The increase in volume fraction from H2 to H4 matches the increase from H4 to H5, yet the $\kappa_{\mathrm{eff}}$ payoff is much smaller in H5, suggesting the added EG does not proportionally reinforce the transport‐limiting segments of the spanning backbone. We therefore extracted the cross‐sectional area of the spanning EG phase and the corresponding heat throughput along the imposed gradient. Figure 6B plots the worm cross‐sectional area $A_{\mathrm{worm}}$ as a function of x as a solid red curve and the normalized slice throughput $Q_{\mathrm{worm}}$ as a dashed blue curve across the H5 RVE. Both profiles show a broad interior region with high area and throughput, punctuated by constrictions where the worm cross‐section narrows and the slice‐wise throughput drops, before both decay toward zero near the specimen edges where the spanning network terminates.

In H5, sharp reductions in worm cross‐sectional area between x≈0.5–0.75 mm produce only weak changes in $Q_{\mathrm{worm}}$, indicating a throughput‐insensitive regime where the backbone remains effectively overbuilt and the dominant series resistance lies elsewhere. By contrast, once the network narrows past x≈1.2 mm, $Q_{\mathrm{worm}}$ collapses in step with area, identifying bottleneck‐controlled regions that directly throttle the macroscopic response. This behavior explains the diminishing returns with increasing filler fraction. Once a robust percolating backbone is established, additional EG volume primarily thickens non‐limiting segments rather than relieving the critical constrictions that set $\kappa_{\mathrm{eff}}$.

To visualize transport pathways in H5, Figure 6C–E maps the converged FVM heat‐flux solution onto the reconstructed microstructure. Figure 6C shows the percolating EG backbone colored by the local flux magnitude |q|, and Figures 6D–E show a representative XY slice with the temperature field and the corresponding in‐plane flux map with streamlines. The flux‐resolved FVM fields suggest why EG and GNP hybrids outperform either filler alone. In the H5 RVE, the reconstructed EG phase forms a single, highly ramified spanning backbone with strong three‐dimensional branching and cross‐sectional variability. This structure provides a continuous mesoscale pathway for heat transport. Coloring by the local flux magnitude |q| reveals pronounced heterogeneity. Narrow filamentary segments carry high throughput heat flow while a larger volume of worm material carries relatively little. This pattern implies that the macroscopic enhancement is controlled by a limited set of dominant paths rather than by the full worm volume. In turn, network geometry governs intraworm flux localization through local cross section, tortuosity, branching, and necking. The accompanying XY temperature slice shows an almost linear gradient with only minor local perturbations and no sharp voxel scale jumps. This behavior is consistent with a numerically stable steady state solution and with network controlled flux localization.

In Figure 6E, the in‐plane flux magnitude $|q_{xy}|=\sqrt{q_{x}^{2}+q_{y}^{2}}$ is evaluated on a representative XY slice and overlaid with streamlines traced from the smoothed in‐plane field $q_{xy}=\left(q_{x},q_{y}\right)$, with seeds taken from the top ∼2% of $|q_{xy}|$. High $|q_{xy}|$ forms bright islands that coincide with EG worm cross sections, and the color gradients within each island indicate strongly nonuniform intraworm flux that concentrates into preferential subchannels within the backbone, with occasional bright rims consistent with local injection from the surrounding matrix at slice intersections. Since the slice samples only a cross section of the three‐dimensional percolating backbone, the 2D streamlines trace the projected in‐plane flux field $q_{xy}$ on the slice and highlight preferential high‐throughput corridors. Local deflections near GNP‐rich regions indicate that matrix conductivity variations can steer the in‐plane routing where the EG backbone is weak or absent in the section. This view supports an architecture in which a spanning EG backbone carries the dominant current while the GNP‐modified matrix provides in‐plane redistribution and potential bypass under imperfect connectivity.

Figure 6F provides a complementary top‐down view of the same bottleneck physics by collapsing the spanning EG network onto the XY plane and coloring each worm voxel by its local flux magnitude. Rather than a uniform conducting network, the projection resolves into sparse bright filamentary corridors embedded within broader, lower‐flux worm regions, making strong intraworm heterogeneity explicit. They coincide with the transport‐limiting segments identified slice‐wise in Figure 6B, where $Q_{\mathrm{worm}}\left(x\right)$ drops sharply once $A_{\mathrm{worm}}\left(x\right)$ falls below a critical cross section. By contrast, thicker worm regions show diffuse, lower‐intensity flux consistent with a throughput‐insensitive regime in which added EG area thickens non‐limiting segments without proportionate gains in carried current. Figure 6B,F shows that a small number of bottlenecked backbone segments, often near the RVE edges, carry a disproportionate fraction of the worm heat current. Thus, the macroscopic response is governed both by the narrowest constrictions and by how effectively the GNP‐enriched matrix can feed the backbone.

Phase‐level load sharing along the imposed thermal gradient in the x direction is quantified by the cross‐sectional heat flow carried by each phase in Figure 6G. The total cross‐sectional heat flow $Q_{\mathrm{total}}\left(x\right)$ remains nearly constant across the RVE, consistent with steady‐state heat‐flow continuity, although the phase partitioning varies strongly with x. Across the RVE, the percolating EG backbone carries most of the heat load, with cross‐sectional contribution $Q_{\mathrm{EG},\mathrm{perc}}\left(x\right)$, while the matrix carries a smaller but non‐negligible share that increases where the backbone becomes transport‐limiting. As highlighted by the inset in Figure 6G, $Q_{\mathrm{EG},\mathrm{perc}}\left(x\right)$ declines downstream for x≳1.2mm while $Q_{\mathrm{total}}\left(x\right)$ remains nearly unchanged. In the same region, the non‐percolating EG cross‐sectional heat flow increases near the cold side. Although these segments are not connected to the main percolation network and contribute little over much of the RVE, they can still provide local high‐conductivity pathways over short transfer lengths. The matrix and soft‐GNP contributions also rise where the backbone locally constricts, consistent with redistribution into surrounding phases when transport through the percolating EG backbone becomes locally throttled. Figure S22 shows the same phase‐decomposed trend, with the EG network providing the dominant contribution while the full hybrid response is not captured by a purely additive combination of the isolated phase contributions. These results support an additional synergy mechanism in which backbone‐dominated transport is enhanced by cooperative load sharing and flux redistribution among the surrounding phases near local bottlenecks.

## 3 Conclusion

We combined systematic experiments with 3D image‐based thermal transport modeling to reveal how EG worms and GNPs enhance heat conduction in paraffin‐based PCM composites. Hybrid composites reach $\kappa \approx 2.7\pm 0.2\;W\;m^{-1}\;K^{-1}$ at 5wt% total filler, corresponding to a near 1000% enhancement over neat paraffin and consistently exceeding single‐filler baselines. This performance ranks among the highest reported for PCM composites at ϕ≤5wt% while preserving the melting range and latent heat capacity.

To identify the origin of this enhancement, we developed a microCT‐informed FVM framework that resolves the percolating EG backbone explicitly and represents sub‐voxel GNP platelets through a continuous enrichment field. Anchored to the single‐filler response, the model isolates an effective worm‐phase conductivity from the hybrid microstructures, with $\kappa_{\mathrm{worm}}$ increasing from $53-127\;W\;m^{-1}\;K^{-1}$ at 2wt% to $164-221\;W\;m^{-1}\;K^{-1}$ at 5wt%. Because the reconstructed worm geometry, network constrictions, and coupling to the surrounding matrix are already encoded in the FVM, this rise cannot be attributed to connectivity alone and instead indicates a loading‐dependent, GNP‐enabled reinforcement within the effective worm phase. Prior studies have emphasized EG–matrix bridging. Here, the $\kappa_{\mathrm{worm}}$ trends indicate an additional, backbone‐centered contribution to the synergy, consistent with reinforcement of the spanning EG network through possible surface coating, intrapore infiltration or bridging, improved internal EG contacts, and flux redistribution near local bottlenecks. Phase‐resolved flux analysis further indicates redistribution into surrounding phases at geometric bottlenecks.

This framework readily extends to other composite systems and architectures when the microstructure can be represented as segmented 3D domains or smooth indicator fields. New fillers can be incorporated as additional fields with their own constitutive laws, and GPU acceleration enables rapid sweeps that connect processing‐driven microstructure changes to phase‐wise pathways and emergent conductivities. By resolving how multiple fillers interact to carry heat in hybrid composites, this work supports more rational, mechanism‐driven design of thermally conductive composites.

## 4 Materials and Methods

### 4.1 Materials

The PCM matrix was a commercial paraffin wax supplied as white pellets (Grade A, HalalEveryday, USA). Graphene nanopowder was purchased from SkySpring Nanomaterials, Inc. with a thickness of 11–15 nm and an average lateral size of 50 μm. Expandable graphite was purchased from ACS materials with an average particle size of 80 μm, 95% purity, an expansion temperature of 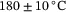, and an expansion volume of >150 $mL\;g^{-1}$. EG was made from the expandable graphite in a microwave oven (Black and Decker) with a power of 700 W for 60–90 s, or until the expansion reaction was visibly over.

### 4.2 Composite Preparation

Pure paraffin wax (7 g) was first melted in a beaker at 90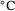 on a hot plate to ensure complete liquefaction for mixing. Once fully liquefied, the required mass of filler(s) was gradually added to the melt and dispersed using a magnetic stir bar at 800 rpm, with manual scraping of the beaker walls every 10 min to prevent edge buildup. The suspension was stirred for 1 h and then ultrasonicated in a hot‐water bath for an additional 1 h to further promote dispersion. The liquid composite was then cast into a silicone mold to form cylindrical pellets and allowed to solidify. A schematic of the preparation workflow is provided in Figure 2A.

### 4.3 Phase and Microstructure Characterization

The EG, GNP, paraffin, and resulting composites were analyzed using X‐ray diffraction (XRD; Cu Kα source, λ=1.54Å). Raman spectroscopy measurements of the EG, GNP, and composites were performed on a Horiba LabRam with a 532 nm coherent sapphire laser at room temperature to verify the composition. Fourier‐transform infrared (FTIR) spectra of the pure paraffin and the three composite types were collected on a Nicolet iS50 FTIR Advanced KBr Gold spectrometer equipped with a built‐in diamond attenuated total reflectance (ATR) module.

The morphology and microstructure of the filler materials and resulting composites were characterized using a TESCAN VEGA3 SBH scanning electron microscope with secondary‐electron (SE) detection at 5–10 kV. The composites were imaged at the UCLA Crump Institute on a benchtop microCT system (80 kV, 0.5 s exposure; 17 μ m isotropic voxels). Raw DICOM series were imported in Python using pydicom and converted to calibrated intensities using the header rescale slope and intercept. Three‐dimensional renderings were produced in ParaView.

### 4.4 Thermal Property Measurements

The heating and cooling curves, as well as the melting points, heat capacities, and latent heats of the composites, were measured using a differential scanning calorimeter (DSC, Netzsch DSC 214 Polyma) from 20℃ to 75℃. Sample masses were in the range of 10–12 mg, and the heating rate was set to 10 K/min under $N_{2}$ atmosphere. The heat capacity was measured using the $C_{p}$ ratio method with a sapphire reference sample. The thermal conductivities of the composites were measured using a Quantum Design Physical Property Measurement System (PPMS) with the Thermal Transport Option (TTO) at room temperature. Further details on the thermal property measurements and error analysis are available in the Supporting Information.

### 4.5 Image‐Based Thermal Transport Modeling

#### 4.5.1 Microstructure Reconstruction

Raw microCT DICOM sequences were stacked and converted into calibrated 3D intensity volumes. To eliminate edge artifacts and varying boundary conditions from the physical sample edges, interior representative volume elements (RVEs) were defined as the largest axis‐aligned rectangular prisms inscribable within the cylindrical sample bulk. The voxel resolution was isotropic at 17μm. Voxel intensities were clipped to the 0.5th–99.5th percentile range to suppress reconstruction outliers. Since the voxel resolution exceeds the thickness of individual nanoplatelets, a probabilistic soft‐GNP enrichment field P(r)∈[0,1] was computed from the local intensity statistics to encode sub‐voxel platelet content (details in Supporting Information). For each composition included in the image‐based analysis, the microCT‐derived RVE and the corresponding experimental room‐temperature thermal conductivity were obtained from the same cylindrical pellet. The resulting RVEs had approximate dimensions of 2 × 3 × 3.5 mm (x × y × z), where the x direction, corresponding to the applied thermal‐gradient direction, spans the full sample height.

#### 4.5.2 Constitutive Mapping and Calibration

Local thermal conductivity, κ(r), was assigned using a dual‐population mixing rule. The matrix phase conductivity was modulated by the probabilistic field P(r) using a constitutive relationship calibrated against the experimental GNP‐composite series, capturing the baseline enhancement from dispersed platelets. This GNP calibration was held fixed for all hybrid simulations. For hybrid composites, the EG worms were explicitly segmented, and a single effective EG worm conductivity ($\kappa_{\mathrm{worm}}$) was determined via inverse modeling to match the experimental κ (calibration details provided in Supporting Information).

#### 4.5.3 Numerical Implementation

Simulations were performed using a custom finite‐volume solver developed in Python and accelerated with CuPy sparse linear algebra libraries on an NVIDIA RTX 4090 GPU. The steady‐state heat diffusion equation, ∇·[κ(r)∇T]=0, was discretized on the native voxel grid using a cell‐centered formulation. Inter‐voxel fluxes were computed using the harmonic mean of adjacent cell conductivities to strictly enforce flux conservation across high‐contrast phase boundaries. Dirichlet boundary conditions established a fixed temperature gradient (ΔT) along the primary loading axis, while periodic boundary conditions were applied to the transverse faces to minimize finite‐size effects (details provided in Supporting Information).

#### 4.5.4 Solver Validation

Convergence was defined by a relative residual tolerance of $10^{-6}$, resulting in global energy balance residuals on the order of $10^{-9}$–$10^{-8}$ (see Figure S9). The solver accuracy was validated against analytical reference problems and by reproducing independent literature values for Diamond/Cu composites to within 0.2% (see Figures S10– S18).

## Author Contributions

X.C. and T.H. co‐designed the study, T.H. wrote the initial manuscript, T.H. performed the analysis and generated all figures. X.C., S.G., J.H., and B.C. contributed to interpretation of the results and revision of the manuscript. X.C. supervised the project and acquired funding.

## Conflicts of Interest

The authors declare no conflicts of interest.

## Supporting information


**Supporting File**: advs75791‐sup‐0001‐SuppMat.pdf.

## Data Availability

The data that support the findings of this study are available from the corresponding author upon reasonable request.
