# Supplementary material for: Beyond Percolation: Graphene‐Enabled Network Reinforcement Enhances Thermal Transport in Paraffin Phase‐Change Composites
Source: Adv Sci (Weinh). 2026 May 26;13(46):e75791. doi: 10.1002/advs.75791 (PMC13335974; doi:10.1002/advs.75791)
Supplement: Supplementary file 1 [file ADVS-13-e75791-s001.pdf]

Supporting Information for

Beyond Percolation: Graphene-Enabled Network Reinforcement Enhances Thermal Transport in Paraffin Phase-Change Composites

Thomas Hoke,[1] Jackson Hoke,[2] Shucheng Guo,[1] Brittany Chan,[1] Xi Chen[1,*]

[1] Department of Electrical and Computer Engineering, University of California, Riverside, California, 92521, USA

[2] Department of Computer Science and Engineering, University of California, Riverside, California, 92521, USA

[*]Correspondence: xichen@ucr.edu

S1 Phase and Optical Characterization

A summary of the material characterization results is shown in Figure S1. The FTIR results in Figure S1a show the transmittance spectrum for paraffin, GNP4, EG4, and H4 samples. Paraffin and the resulting composites containing GNP, EG, and the hybrid mixture of the two show the characteristic peaks at 725 cm^{-1}, 1380 cm^{-1}, 1470 cm^{-1}, and 2940-2855 cm^{-1}, attributable to the CH_2 rocking absorption, symmetric carbon-hydrogen bending absorption of the CH_3 group, and the carbon-hydrogen stretching and bending absorption bands, respectively [1]. The composites show some baseline drift which is due to the change in reflectivity arising from the addition of the fillers.

0

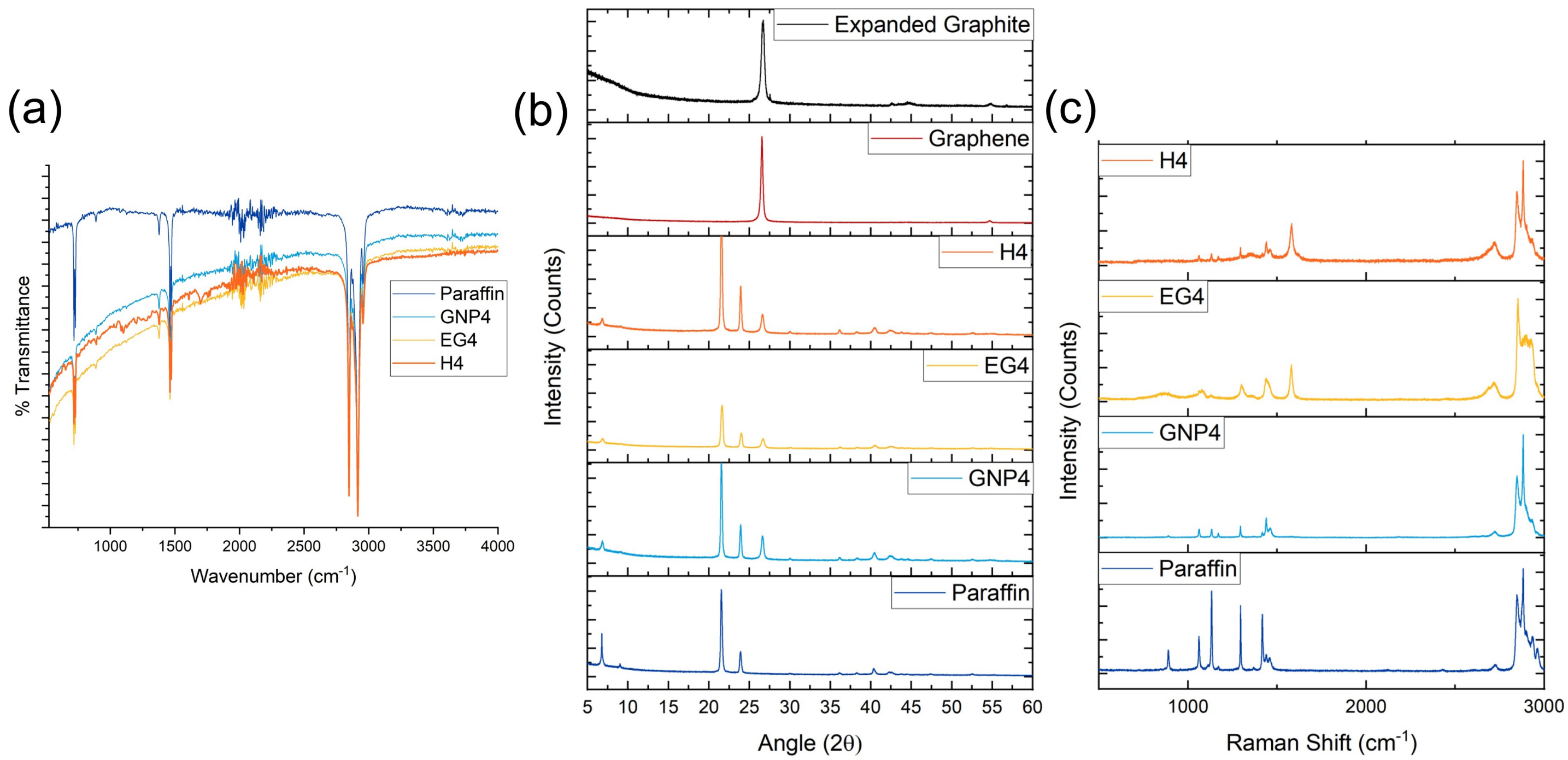

Figure S1: A summary of the chemical characterization results of the individual materials and resulting composites. (a) FTIR (b) XRD (c) Raman Spectroscopy

The XRD results are shown in Figure S1b, with paraffin showing characteristic peaks near $2\theta = 22°$ and 24°. Both the expanded graphite and graphene nanoplatelet samples show the characteristic pristine graphitic peak at $2\theta = 26.6°$ [2]. The EG4, GNP4, and H4 composite samples show a combination of the $2\theta = 22°$ and 24° peaks from paraffin and the $2\theta = 26.6°$ peak from the graphitic filler inclusions, indicating no chemical interaction between the PCM matrix and fillers, or interactions between the EG and GNP fillers in the hybrid sample.

Raman spectra of EG4, GNP4, and H4 in Figure S1c show the expected superposition of paraffin chain vibrations and graphitic sp^2-carbon modes. The paraffin matrix contributes the characteristic alkyl-chain bands, including C–C skeletal stretching near ~ 1060 and ~ 1130 cm^{-1}, a CH_2 twisting band near ~ 1300 cm^{-1}, and strong C–H stretching bands in the 2800–3000 cm^{-1} range [3]. The EG-containing samples (EG4 and H4) additionally show a pronounced graphitic G band near ~ 1580 cm^{-1}, consistent with sp^2 C–C bond stretching under visible excitation [4]. In the GNP4 composite, the spectrum is dominated by paraffin vibrations. The graphitic G band is weak at the probed locations and is not clearly resolved at the scale shown, consistent with Raman's localized sampling and a paraffin-rich surface.

S2 Thermal conductivity measurements and uncertainty analysis

Bulk thermal conductivity of the paraffin, EG, GNP, and hybrid pellets was measured using the thermal transport option (TTO) of a Quantum Design Physical Property Measurement System (PPMS). Cylindrical pellets with nominal diameter $d \approx 6$ mm and thickness $t \approx 2$–3 mm were mounted on the TTO puck in the standard two-probe lead configuration, with the pellet sandwiched between the hot and cold thermometer blocks using a thin layer of silver paint to ensure reproducible thermal contact. In this geometry, the thermal resistance of the paraffin-based pellets is much larger than that of the silver paint and copper pads in the 280–300 K range, so the additional series contact resistance is negligible and effectively absorbed into the reported conductivity. The effective length L was taken as the center-to-center distance between the thermometers, and the cross-sectional area was $A = \pi(d/2)^2$.

For each run, the cryostat temperature was swept from 300 to 280 K at a rate of 0.3 $\mathrm{K\,min^{-1}}$ in continuous mode. The TTO module applied a square-wave heating pulse and the heater power and period were adjusted automatically [5]. At each temperature, the PPMS software recorded the time-dependent temperature difference $\Delta T(t)$ between the hot and cold thermometers and fit it to the standard two-time-constant model implemented for the TTO option. From this fit, the steady-state temperature rise ΔT_{ss} and characteristic time constants $\tau_{1,2}$ were obtained.

The total thermal conductance between the thermometers,

$$K(T) = \frac{P}{\Delta T_{\mathrm{ss}}}, \tag{1}$$

was computed using the net heater power P after subtracting radiative losses. The heater power is given by

$$P = I^2 R - P_{\mathrm{rad}}, \tag{2}$$

where I and R are the heater current and resistance, and P_{rad} is the radiative loss estimated from the Stefan–Boltzmann law using the sample surface area and an effective emissivity. In the narrow temperature window of 280–300 K, P_{rad} is much smaller than P and contributes only a minor correction to $K(T)$. The sample thermal conductance is obtained by subtracting the pre-calibrated conductance of the addenda (shoes + silver paint),

$$K_{\mathrm{sample}}(T) = K(T) - K_{\mathrm{shoe}}(T), \tag{3}$$

where $K_{\mathrm{shoe}}(T)$ is represented by a low-order polynomial fit in T determined from a separate addenda calibration. Assuming one-dimensional heat flow between the thermometers, the bulk thermal conductivity is

$$\kappa(T) = K_{\mathrm{sample}}(T)\,\frac{L}{A}, \tag{4}$$

with L and A defined above.

Uncertainties in κ arise from (i) the fit to $\Delta T(t)$, (ii) the determination of the heater power (including the resistance and current calibration and the small radiative correction), (iii) the calibration of $K_{\mathrm{shoe}}(T)$, and (iv) measurement of the sample dimensions (L, d, and t). The uncertainty in the steady-state rise, $\sigma_{\Delta T}$, was estimated from the root-mean-square deviation between the measured $\Delta T(t_i)$ and the fitted model over all time points in a given pulse. The relative uncertainty in κ was then obtained by standard error propagation,

$$\left[\frac{\sigma(\kappa)}{\kappa}\right]^2 = \left[\frac{\sigma_{\Delta T}}{\Delta T_{\mathrm{ss}}}\right]^2 + \left[\frac{\sigma_P}{P}\right]^2 + \left[\frac{\sigma_{K_{\mathrm{shoe}}}}{K_{\mathrm{sample}}}\right]^2 + \left[\frac{\sigma_L}{L}\right]^2 + \left[\frac{\sigma_A}{A}\right]^2, \tag{5}$$

where σ_P captures uncertainties in the heater power (including the small radiative-loss correction), $\sigma_{K_{\mathrm{shoe}}}$ denotes the uncertainty in the shoe conductance calibration, and σ_L and σ_A denote uncertainties in the geometric factors. For our pellets, the geometric terms dominate, with the radiative-loss and addenda terms contributing much smaller amounts. The error bars on the room-temperature thermal conductivities correspond to $\pm\sigma(\kappa)$ from this combined uncertainty.

S3 Image-based modeling framework

Here, we seek a mechanistic interpretation of the bulk conductivity in hybrid EG+GNP composites by quantifying synergistic filler interactions and extracting a worm-phase conductivity κ_{worm} that agrees with macroscopic measurements. To do this, we develop a microstructure-resolved, image-based modeling framework that links the measured effective conductivity directly to the three-dimensional architecture of the expanded-graphite (EG) worm network, the surrounding paraffin-rich matrix, and GNPs.
A key difficulty is that the microCT voxel size is comparable to or larger than the GNP thickness, so GNPs do not appear as resolved inclusions. Instead, they brighten voxels that also contain paraffin (and, in hybrids, EG). Consequently, the GNP contribution to heat transport cannot be separated via hard three-phase segmentation. We therefore treat GNP enrichment as composite in each voxel and determine its transport effect through calibration on GNP-only composites before applying the same calibrated description to the hybrids, leaving κ_{worm} as the sole unknown effective property of the EG worms.
The framework proceeds in four stages. (i) For each composition, we reconstruct a microCT volume, select an interior representative volume element (RVE), and extract image-derived phase fields, a binary EG phase, a pore mask, and a probabilistic soft-GNP field that encodes local GNP enrichment where platelets are sub-voxel. (ii) These geometric and probabilistic fields are converted into voxel-level conductivity tensors using a constitutive model that treats EG worms as a distinct effective phase and maps the soft-GNP field into an orientation-averaged GNP+paraffin mixture law. (iii) Using the GNP-only composites, we calibrate the parameters of this GNP mixture model by matching image-based finite-volume predictions of $\kappa_{\mathrm{eff},x}$ to experimental conductivities, thereby capturing the unresolved platelet contribution to heat transport. (iv) With the GNP mixture parameters fixed, we apply the same model to the hybrid RVEs and scan κ_{worm} until the FVM-predicted $\kappa_{\mathrm{eff},x}$ matches the measured conductivities, isolating κ_{worm} and quantifying the relative contributions of the EG network and the paraffin+GNP solid.
In this framework, the microCT data fully determine the mesoscale geometry (worm connectivity, pore structure, and the spatial pattern of GNP enrichment), while the remaining constitutive parameters are constrained by bulk measurements. Together, these ingredients not only reproduce the effective conductivities across compositions, but also yield voxel-resolved heat-flux fields that decompose hybrid transport into worm-mediated and matrix-mediated paths, identify transport bottlenecks, and quantitatively assess synergistic interactions between the EG worms and GNP platelets in these dual-filler composites.

S3.1 DICOM volume reconstruction and definition of the representative volume element

MicroCT data were provided as DICOM image stacks and converted to calibrated 3D intensity volumes by sorting slices by physical z position and applying the standard linear rescale from the DICOM metadata. The in-plane voxel spacing (dx, dy) was taken from the pixel spacing tag and dz was set to the median inter-slice spacing; when multiple images shared the same z position, they were averaged to form a single plane. All subsequent analysis was performed on a manually defined, axis-aligned interior RVE. In orthogonal views, we first identified the cylindrical specimen cross-section and then selected the largest inscribed rectangular prism that fits within this cylinder without intersecting the specimen boundary, which avoids edge and mounting artifacts by construction, shown in Figure S2. The analysis domain is the subvolume $V_{\mathrm{RVE}} = V[z_0{:}z_1,\ y_0{:}y_1,\ x_0{:}x_1]$.

S3.2 ROI intensity distribution and robust clipping

Let $V_{\mathrm{RVE}}(z, y, x)$ denote the reconstructed intensity volume. To suppress boundary artifacts, all intensity statistics are computed over an interior mask $M(z, y, x)$ that excludes a thin margin at the RVE faces. We then apply percentile-based clipping to the masked intensities $\{v_i \mid M = 1\}$ to reduce sensitivity to rare extremes (e.g., beam hardening, streaks, or isolated high-density inclusions). We clip masked intensities to the 0.5th–99.5th percentile range, retaining voxels with $v_{\mathrm{low}} \leq v_i \leq v_{\mathrm{high}}$. All subsequent classification operates on this clipped intensity set, which removes a small outlier fraction while preserving the bulk matrix and GNP-related intensity structure. The clipped 1D histogram can be seen as the blue curve in Figure S3a.

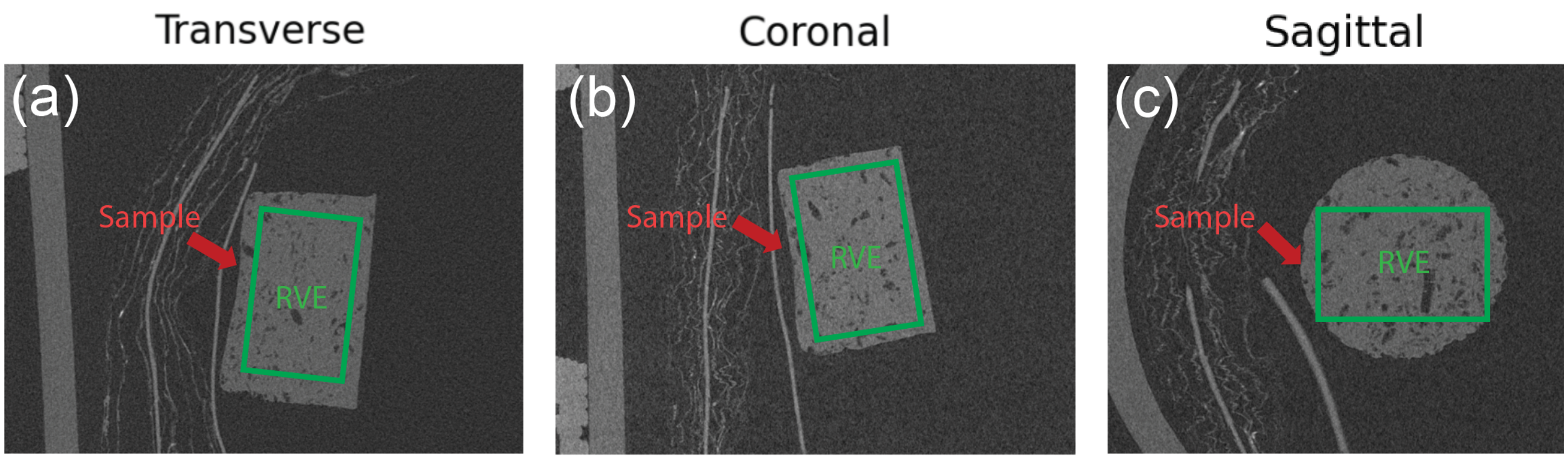

Figure S2: Orthogonal **transverse** (a), **coronal** (b), and **sagittal** (c) views of a representative raw microCT reconstruction. The sample is indicated by red arrows, and the selected representative volume element (RVE) is outlined in green.

S3.3 Volume Fraction Analysis

The resulting volume fractions within the RVE are summarized in Table S1. Here, ϕ_{EG} denotes the expanded-graphite volume fraction in the hybrid samples. For H2, H4, and H5, $\phi_{\mathrm{EG}} \approx 0.11$–$0.17$, with an additional $\sim 1\%$ pore volume fraction outside the EG mask. By contrast, the EG-only samples (EG2, EG4) contain only $\phi_{\mathrm{EG+pores}} \approx 0.008$–$0.016$ of low-intensity material in total, even when pores are included. In other words, introducing GNP increases the amount of contiguous EG-like phase by roughly an order of magnitude. Because the composites share the same mixing and sonication procedure, this disparity points to a stabilizing role of GNP during processing. One possible mechanism is that GNP platelets embedded in or adjacent to the worms can stiffen the walls, redistribute local stresses, and help prevent collapse or fragmentation of the pore-rich worm structure. The microCT measurements therefore indicate that worm survival is substantially higher in the hybrid formulations than in the EG-only composites.

Table S1: Phase volume fractions within the microCT RVE for hybrid and EG-only composites. For H2, H4, and H5, ϕ_{EG} is the volume fraction of voxels classified as expanded graphite and ϕ_{pore} is the pore fraction outside the EG mask. For EG2 and EG4, ϕ_{EG} denotes the combined volume fraction of low-intensity voxels attributed to EG remnants and pores, which cannot be reliably separated.

Sample	ϕ_{EG}	ϕ_{pore}
H2	0.107	0.0096
H4	0.137	0.0140
H5	0.167	0.0122
EG2	0.0077	–
EG4	0.0160	–

S3.4 Multi-threshold and mixture models for phase identification

To demonstrate that the matrix and GNP phases are separable by brightness and to obtain consistent class statistics, we analyze the clipped intensity values using two complementary classifier families, namely histogram-based multi-thresholding and parametric Gaussian mixture modeling.

Multi-level Otsu thresholding

As a non-parametric benchmark, multi-level Otsu thresholding is applied to the clipped intensity histogram, implemented using `scikit-image` [6, 7, 8]. In the three-class setting, denoted `multi3`, two thresholds $t_1 < t_2$ are chosen to partition the intensities into $(-\infty, t_1]$, $(t_1, t_2]$, and (t_2, ∞) by minimizing the within-class variance. Voxels are assigned to the resulting three classes based on their intensity. The classes are then ordered by mean intensity. The darkest class is interpreted as pores or voids, and the two brighter classes are interpreted as candidate matrix and GNP-rich phases. Among the two brighter classes, the class with the larger voxel count is labeled as the paraffin-rich matrix, and the remaining class is labeled as the GNP-rich phase.

In the four-class setting, denoted `multi4`, three thresholds are selected to produce four classes. After sorting by mean intensity, the two darkest classes are combined and interpreted as pores or EG. The two brighter classes are then assigned

as matrix and GNP-rich phases using the same occupancy rule, with the more populous class labeled as matrix. This configuration provides added flexibility for broad pore intensity distributions while retaining a two-class separation between matrix and GNP within the solid.

Gaussian mixture modeling

As a complementary parametric approach, a three-component Gaussian mixture model (GMM) is fit to the clipped intensity values. The model assumes the intensity probability density within the RVE can be written as

$$p(v) = \sum_{k=1}^{3} w_k \mathcal{N}\big(v \mid \mu_k, \sigma_k^2\big) \tag{6}$$

where v denotes a microCT intensity value drawn from the masked voxel set $\{v_i \mid M = 1\}$ within the representative volume element (RVE), and $p(v)$ is the corresponding probability density of these intensities in the RVE. The index $k \in \{1, 2, 3\}$ labels the three Gaussian components, $w_k \geq 0$ are the mixture weights satisfying $\sum_{k=1}^{3} w_k = 1$, and $\mathcal{N}(v \mid \mu_k, \sigma_k^2)$ denotes a normal density in v with mean μ_k and variance σ_k^2 (standard deviation σ_k) [9]. Parameters are estimated by maximum likelihood using the expectation–maximization algorithm, with initial means chosen to span the lower, central, and upper portions of the observed histogram [10]. After convergence, the components are ordered by their fitted means. The lowest-intensity component is interpreted as pores or voids, while the middle- and highest-intensity components are interpreted as matrix and GNP-rich phases, respectively.

For each voxel at position $\mathbf{r}$, the fitted model yields responsibilities $r_k(\mathbf{r})$ that sum to one and quantify soft membership in each component. For the phase separability analysis in this section, these responsibilities are converted to hard labels by assigning each voxel to the component with the largest responsibility. This produces a three-class segmentation of the clipped intensities into pores, matrix, and GNP that can be directly compared to the multi-threshold segmentation described above.

S3.5 Global separation metrics

For each sample and for each segmentation method, voxels labeled as matrix and as GNP are extracted and their empirical means and standard deviations are computed as μ_{paraffin}, σ_{paraffin}, μ_{GNP}, σ_{GNP}. Let n_{paraffin} and n_{GNP} denote the corresponding voxel counts. The brightness contrast between phases is quantified by

$$\Delta = \mu_{\text{paraffin}} - \mu_{\text{GNP}}, \tag{7}$$

where $\Delta < 0$ corresponds to the physically expected case in which GNP-rich voxels are brighter than the paraffin-rich matrix. Assuming the sample means are approximately normally distributed, the standard error of Δ is estimated as

$$\text{SE}(\Delta) = \sqrt{\frac{\sigma_{\text{paraffin}}^2}{n_{\text{paraffin}}} + \frac{\sigma_{\text{GNP}}^2}{n_{\text{GNP}}}}, \tag{8}$$

and a nominal 95% confidence interval is reported as $\Delta \pm 1.96\,\text{SE}(\Delta)$. This interval summarizes uncertainty in the mean brightness difference between matrix and GNP within the selected RVE.

Separability is further characterized using an intensity-threshold classifier that discriminates matrix and GNP. The matrix and GNP class histograms are approximated as Gaussian distributions with means μ_{paraffin} and μ_{GNP} and standard deviations σ_{paraffin} and σ_{GNP}. The corresponding receiver operating characteristic curve is evaluated numerically and its area under the curve is computed. Because these metrics are derived from Gaussian approximations to the class histograms, the resulting area under the curve and misclassification probabilities should be interpreted as measures of distributional overlap rather than voxel-wise error rates. An operating threshold is identified near the intersection of the two Gaussian probability density functions, and the associated false positive and false negative probabilities are reported for matrix classified as GNP and GNP classified as matrix.

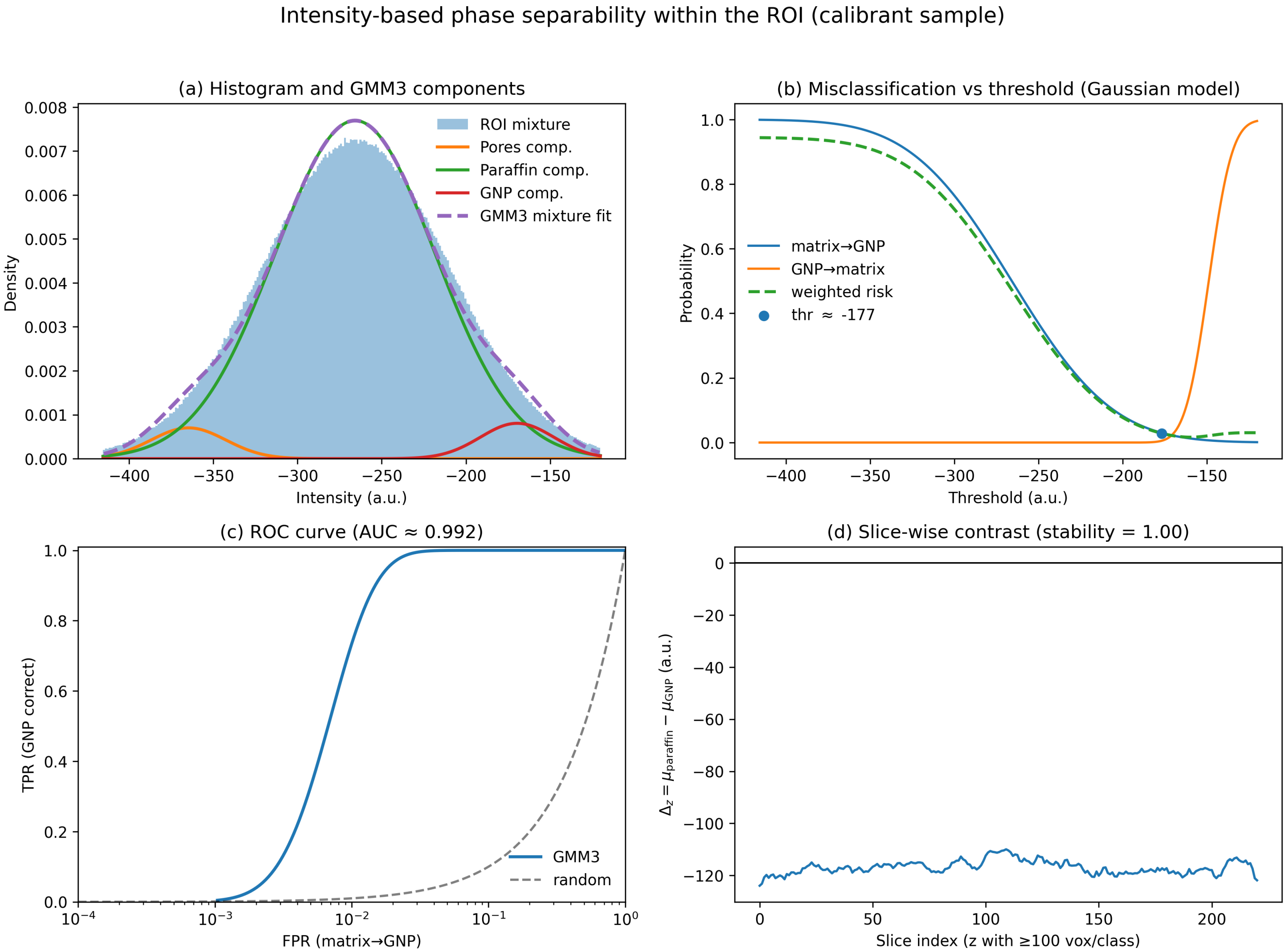

Figure S3: Intensity-based phase separability in the calibrant ROI. (**a**) One-dimensional intensity histogram of the interior ROI, overlaid with the fitted GMM3 components for paraffin and GNP, together with their weighted sum and the operating threshold. (**b**) Misclassification probabilities for paraffin mislabeled as GNP (matrix→GNP) and GNP mislabeled as paraffin (GNP→matrix) as a function of intensity threshold, along with the corresponding weighted risk. (**c**) Receiver operating characteristic (ROC) curve for the GMM3 classifier on the same ROI, with area under the curve (AUC) reported in the panel. (**d**) Slice-wise intensity contrast $\Delta_z = \mu_{\text{paraffin}} - \mu_{\text{GNP}}$ across all z-slices containing at least 100 voxels of each class, together with a simple stability metric that quantifies how consistently the sign of Δ_z is preserved along the ROI.

Figure S3**a** shows that the pooled ROI histogram is often close to unimodal and that the GNP population appears primarily as a bright-tail shoulder that is isolated by the fitted GMM components. Figure S3**b** quantifies the matrix to GNP and GNP to matrix tradeoff under the Gaussian overlap model as the threshold is swept and identifies an operating threshold near the intersection of the two Gaussian probability density functions. Figure S3**c** confirms strong discrimination on the calibrant ROI through a high ROC area under the curve.

The pooled separability metrics above imply $\Delta < 0$, i.e., GNP-rich voxels are brighter than the paraffin-rich matrix. Figure S3**d** tests whether this ordering is preserved along the scan direction. For each axial slice z, voxels within the interior mask M that are labeled as matrix or GNP are used to compute the slice-wise class means $\mu_{\text{paraffin}}(z)$ and $\mu_{\text{GNP}}(z)$, and the slice-wise contrast

$$\Delta_z = \mu_{\text{paraffin}}(z) - \mu_{\text{GNP}}(z) \tag{9}$$

is evaluated after excluding slices with fewer than a minimum voxel count in either class (typically 100). Stability is quantified by the sign-consistency fraction

$$S = \frac{1}{N_{\text{valid}}} \sum_z \mathbf{1}[\text{sign}(\Delta_z) = \text{sign}(\Delta)], \tag{10}$$

where N_{valid} is the number of retained slices. In Figure S3**d**, $\Delta_z < 0$ for all valid slices, giving $S = 1.00$ and confirming slice-wise preservation of the ordering; across samples, trusted methods yield similarly high stability (typically $S \gtrsim 0.95$),

with Δ_z varying weakly about the pooled value. This supports using intensity contrast to construct the probabilistic occupancy fields for the transport model.
In subsequent analysis, the three-component GMM is treated as the primary, physically informed segmentation because it models the ROI intensity histogram as a three-phase mixture and naturally isolates the small bright tail in the measured ROI intensity distribution, yielding a sparse bright class attributed to GNP-bearing voxels. The fit is initialized using the GNP fraction implied by the sample's nominal composition. This improves convergence and reduces class-label ambiguity without imposing a hard constraint, since the final mixture weights and voxel labels are still determined by the intensity data. The multi-level Otsu segmentations `multi3` and `multi4` are retained as prior-free histogram baselines.
Figure S4a shows that all trusted classifiers agree on the sign and magnitude of the GNP to paraffin brightness contrast and report confidence intervals that exclude zero. Figure S4b shows consistently high AUC across classifiers, indicating that separability is not sensitive to the choice of method. Across all samples, the GMM is required to satisfy $\Delta < 0$, a 95% confidence interval for Δ that lies entirely below zero, and a high area under the curve that is typically at least 0.9. Agreement of the Otsu baselines with the GMM is checked for the sign of Δ and for a high area under the curve, but their voxel fractions and masks are not used for quantitative modeling because they tend to overestimate the bright GNP phase. When the GMM criteria are satisfied, the matrix and GNP classes are taken to be separable by intensity within the RVE and the intensity spectrum is taken to be suitable for constructing the soft GNP enrichment fields used in the transport model.

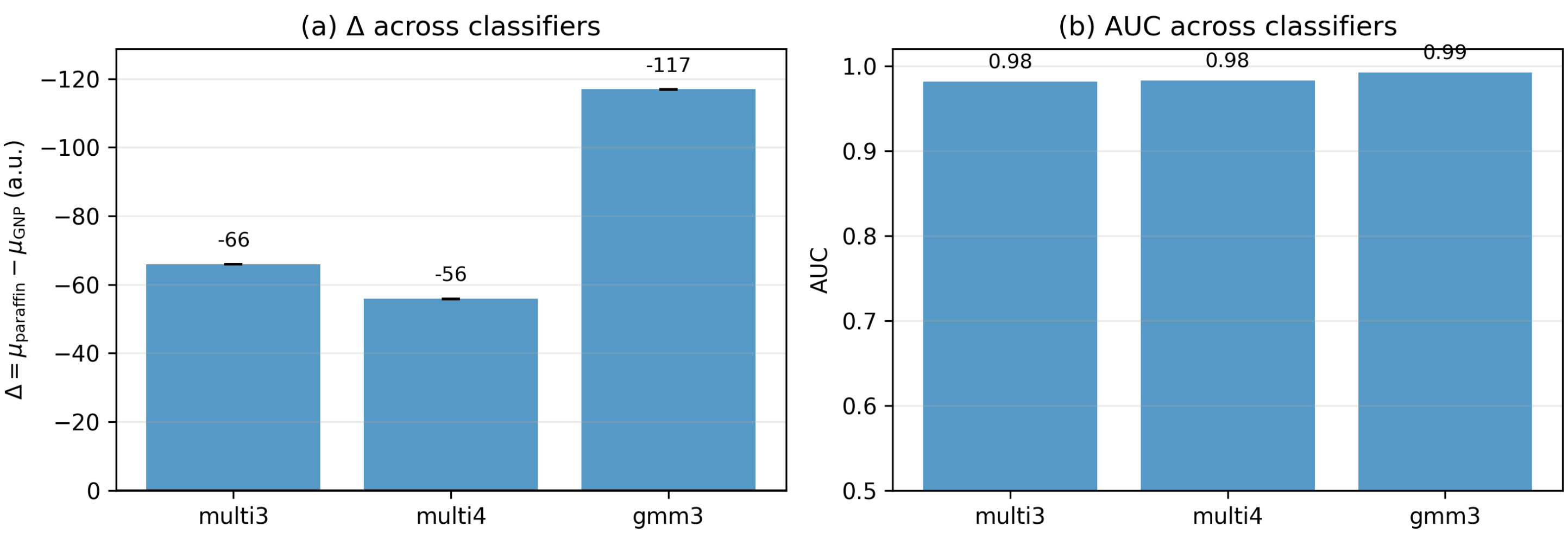

Figure S4: Classifier-level summary of phase separability in the calibrant ROI. (**a**) Global brightness contrast $\Delta\mu = \mu_{\text{paraffin}} - \mu_{\text{GNP}}$ for the trusted classifiers (multi-level Otsu with three and four classes, and GMM3), shown as bar plots with 95% confidence intervals obtained from voxel-level statistics. (**b**) Corresponding AUC values for the same classifiers, summarizing their ROC performance on the calibrant ROI. Together, these metrics show that all trusted methods agree on the sign and magnitude of the GNP–paraffin intensity contrast and yield high AUC values, supporting robust phase separability.

S3.6 Effective intensity resolution and quantization

MicroCT reconstructions are stored with finite gray-level resolution. The effect of intensity quantization is checked relative to the intrinsic spread of the matrix and GNP intensity populations used to construct the soft-GNP fields. Within the interior mask ($M = 1$), a representative gray-level increment is estimated as the median spacing between adjacent unique intensity values, denoted ΔI_{step}. We then define a dimensionless quantization ratio by normalizing this step by a pooled within class standard deviation,

$$\sigma_{\text{pooled}} = \tfrac{1}{2}\big(\sigma_{\text{paraffin}} + \sigma_{\text{GNP}}\big), \qquad \text{QNR} = \frac{\Delta I_{\text{step}}}{\sigma_{\text{pooled}}}, \tag{11}$$

where σ_{paraffin} and σ_{GNP} are the matrix and GNP class standard deviations obtained from the intensity fit. For the representative RVE analyzed here, this procedure gives QNR = 0.00717. Thus, the discrete gray-level step is far smaller than the natural width of the matrix and GNP intensity distributions, and quantization does not limit matrix–GNP separability or the construction of smooth probabilistic GNP fields.

S3.7 MicroCT RVEs and probabilistic phase fields

All phase fields used in the finite-volume modeling are extracted from the interior RVE defined in the preceding section. Figure S5**a** shows a representative xy slice of the reconstructed Hounsfield-unit (HU) intensities within this domain. Low-attenuation regions appear dark and correspond to either EG or void space, while the paraffin matrix forms the dominant

mid-intensity background. Superimposed on this background are localized higher-attenuation features, which we interpret as mixed voxels with elevated GNP content under the present voxel size (17 μm).
We segment the EG worms by thresholding the low-HU tail and retaining only large connected components, which robustly isolates the elongated, percolating morphology. Because pores and EG can overlap in HU, a pore mask is defined only after removing the connected EG phase. Residual low-attenuation regions that are not part of the worm network are treated as voids and assigned the thermal conductivity of air ($\kappa = 0.026\ \mathrm{W\,m^{-1}\,K^{-1}}$) in the subsequent simulations.
To represent the unresolved GNP contribution without imposing a hard global threshold, we construct a soft-GNP occupancy field from the interior HU intensities. We fit a three-component Gaussian mixture model (GMM3) to the clipped histogram, with components corresponding to (i) low attenuation (EG/pore), (ii) matrix, and (iii) high attenuation (GNP-rich). The fitted mixture provides voxel-wise posterior responsibilities

$$R_{\mathrm{pore/EG}}(\mathbf{r}),\ R_{\mathrm{paraffin}}(\mathbf{r}),\ R_{\mathrm{GNP}}(\mathbf{r}), \qquad R_{\mathrm{pore/EG}} + R_{\mathrm{paraffin}} + R_{\mathrm{GNP}} = 1, \tag{12}$$

from which we define the scalar soft-GNP field on non-pore, non-EG voxels by renormalizing the matrix and GNP components:

$$P(\mathbf{r}) = \frac{R_{\mathrm{GNP}}(\mathbf{r})}{R_{\mathrm{paraffin}}(\mathbf{r}) + R_{\mathrm{GNP}}(\mathbf{r})}, \qquad 0 \le P(\mathbf{r}) \le 1. \tag{13}$$

Figure S5**b** visualizes $P(\mathbf{r})$ on the same slice. The field exhibits a low baseline across most of the matrix, punctuated by spatially localized high-P regions that coincide with the brightest HU features in panel **a**. This pattern is consistent with partial-volume mixing at the voxel scale. Rather than assigning all moderately bright voxels to a discrete GNP phase, the soft field preserves intermediate occupancies and yields a smooth, scan-to-scan comparable measure of local enrichment.
Figure S5**c** overlays the HU-derived EG mask (red outline) on $P(\mathbf{r})$. The binary EG network captures the connected worm backbone, while $P(\mathbf{r})$ encodes graded GNP enrichment in the surrounding solid matrix.
It is important to note that $P(\mathbf{r})$ is not interpreted as a literal local GNP volume fraction. It is an intensity-based occupancy weight used to modulate the local solid-phase conductivity within the RVE. The absolute GNP loading is constrained separately by the nominal composition and by calibration of the matrix–GNP conductivity response to the measured effective conductivities.

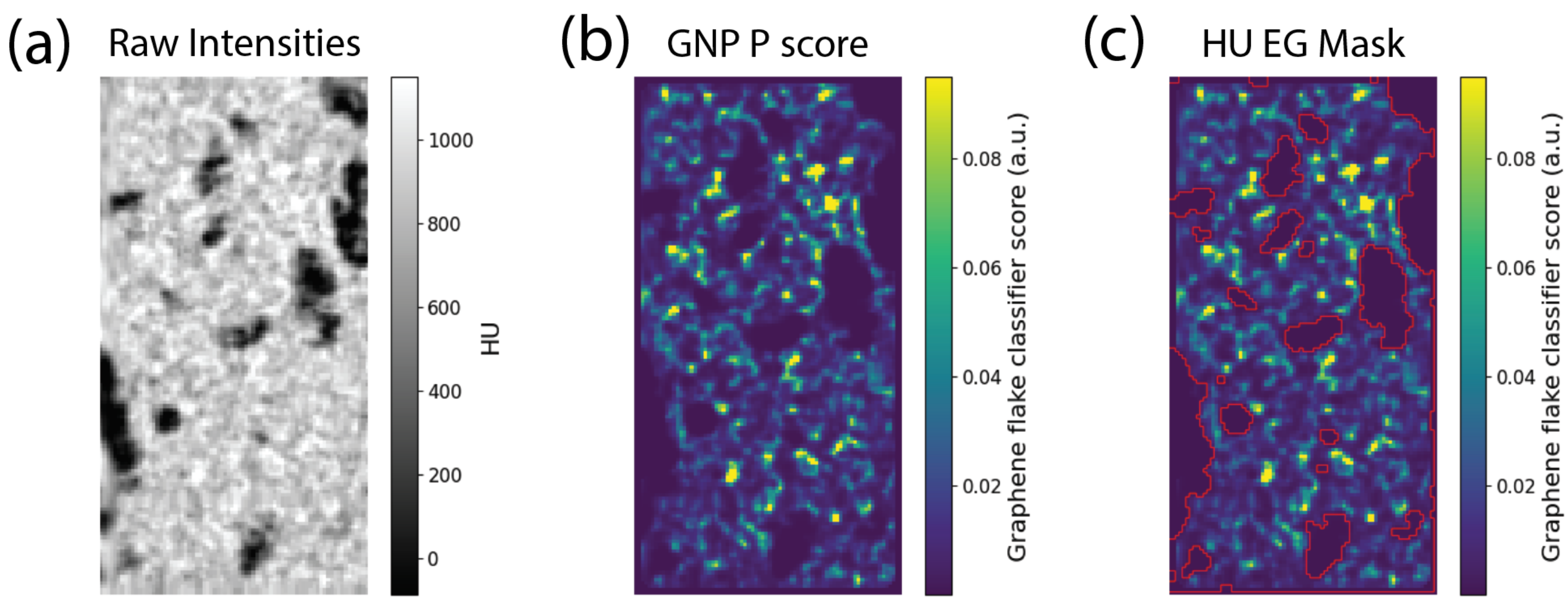

Figure S5: (a) Example xy slice of microCT Hounsfield-unit (HU) intensities within the RVE. (b) Soft-GNP field $P(\mathbf{r})$ inferred from the HU intensities. (c) EG mask (red outline) overlaid on $P(\mathbf{r})$.

S3.8 Voxel-level constitutive model

The soft GNP fields described above are next converted into a local conductivity tensor for each voxel in the RVE. We treat each voxel as a homogeneous continuum element with a diagonal conductivity tensor

$$\kappa(\mathbf{r}) = \begin{bmatrix} \kappa_{xx}(\mathbf{r}) & 0 & 0 \\ 0 & \kappa_{yy}(\mathbf{r}) & 0 \\ 0 & 0 & \kappa_{zz}(\mathbf{r}) \end{bmatrix}, \tag{14}$$

whose entries depend on whether the voxel belongs to the EG mask, the pore mask, or the paraffin+GNP solid.
Voxels belonging to the segmented EG network are modeled as a distinct "worm" phase with effective conductivity κ_{worm}. This quantity represents a voxel-scale homogenized conductivity of the porous EG composite and captures both conduction

through the graphite ligament framework and the mean resistance associated with worm–matrix contacts. Worm–matrix interfacial effects are therefore embedded intrinsically in κ_{worm}, and we do not introduce a separate EG–matrix interface resistance parameter in the finite-volume model. Here, κ_{worm} is taken to be isotropic at the voxel level, with anisotropy emerging primarily from the geometry and connectivity of the percolating worm network rather than from an imposed intrinsic directional conductivity.

To convert the soft-GNP occupancy field $P(\mathbf{r}) \in [0,1]$ into an effective local filler loading, we define a proxy GNP volume fraction $\phi_{\mathrm{GNP}}(\mathbf{r})$ that scales with $P(\mathbf{r})$ through a per-sample factor s_{soft} and is limited by a maximum value $\phi_{\max}$. Here s_{soft} sets the overall enrichment level for a given scan, while $\phi_{\max}$ prevents unrealistically high local fractions.

Within the paraffin+GNP solid, we model in-plane conduction (along x and y) using a generalized Voigt–Reuss interpolation between admissible effective-medium bounds. The Voigt and Reuss mixing rules provide classical upper and lower bounds on the homogenized thermal-conductivity tensor of a two-phase mixture, corresponding to uniform temperature-gradient and uniform heat-flux idealizations. For the conductivity component parallel to the imposed thermal gradient, the voxel-scale matrix–GNP mixture conductivity $\kappa_{\parallel}^{\mathrm{mix}}$ satisfies $\kappa_{\parallel}^{\mathrm{R}} \le \kappa_{\parallel}^{\mathrm{mix}} \le \kappa_{\parallel}^{\mathrm{V}}$, where $\kappa_{\parallel}^{\mathrm{V}}$ and $\kappa_{\parallel}^{\mathrm{R}}$ are the corresponding Voigt (uniform-gradient) and Reuss (uniform-flux) bounds. [11, 12] The arithmetic mean of these bounds defines the Hill (Voigt–Reuss–Hill, VRH) estimate in the special case of a spatially uniform weight $w = 1/2$ [13, 14, 15].

Here we extend the VRH-type interpolation by introducing a microstructure-informed, spatially varying connectivity weight $w(\mathbf{r}) \in [0,1]$ that blends a lower (series-like) Reuss bound $k_{\parallel}^{\mathrm{R}}(\phi_{\mathrm{GNP}})$ with a conservative upper bound $\kappa_{\parallel}^{\mathrm{U}}(\phi_{\mathrm{GNP}})$. In practice, $\kappa_{\parallel}^{\mathrm{U}}$ is defined as the minimum of the Voigt estimate, a Hashin–Shtrikman-type thermal upper bound, and a physical cap $\kappa_{\parallel,\mathrm{cap}}$ to prevent unrealistically large local conductivities [16, 17]. The resulting local conductivity is

$$\kappa_{\parallel}(\mathbf{r}) = \big(1 - w(\mathbf{r})\big)\, \kappa_{\parallel}^{\mathrm{R}}\big(\phi_{\mathrm{GNP}}(\mathbf{r})\big) + w(\mathbf{r})\, \kappa_{\parallel}^{\mathrm{U}}\big(\phi_{\mathrm{GNP}}(\mathbf{r})\big), \tag{15}$$

and we set $\kappa_{xx} = \kappa_{yy} = \kappa_{\parallel}$. In this context, $w(\mathbf{r})$ acts as a local connectivity indicator. Values $w \approx 0$ highlight Reuss-like, bottlenecked regions, whereas $w \approx 1$ favors upper-bound-like, well-connected in-plane pathways. Physically, the Voigt and Reuss limits bracket thermal transport by invoking two idealized local field states. The Voigt (parallel-like) case assumes a uniform temperature gradient across phases, whereas the Reuss (series-like, bottlenecked) case assumes a uniform heat flux. The true effective response must therefore lie between these bounds [11, 12]. Accordingly, we treat $\kappa_{\parallel}^{\mathrm{R}}$ and the Voigt-based estimate as admissible voxelwise lower and upper limits, and enforce conservatism by taking their upper envelope through the intersection with the Hashin–Shtrikman-type bound and $k_{\parallel,\mathrm{cap}}$ [16, 17].

To connect this voxelwise interpretation of $w(\mathbf{r})$ to the underlying soft-GNP field, we first define the standardized field

$$\tilde{P}_{\mathrm{GNP}}(\mathbf{r}) = \frac{P(\mathbf{r}) - \langle P \rangle}{\mathrm{std}(P)}, \tag{16}$$

where $\langle P \rangle$ and $\mathrm{std}(P)$ are computed over interior voxels, so that $\tilde{P}_{\mathrm{GNP}}$ has zero mean and unit variance. Here $w(\mathbf{r})$ is then obtained by mapping $\tilde{P}_{\mathrm{GNP}}$ through a logistic function,

$$w(\mathbf{r}) = \sigma\big(\alpha + \beta\, \tilde{P}_{\mathrm{GNP}}(\mathbf{r})\big), \qquad \sigma(z) = \frac{1}{1 + e^{-z}}, \tag{17}$$

This logistic projection is a standard smooth-Heaviside (sigmoid) mapping used to convert a continuous indicator field into a bounded phase/connectivity weight in $[0,1]$. The parameter β controls the steepness of the transition between matrix-like and GNP-like regions, and α is chosen so that the interior-RVE mean of the field satisfies $\langle w \rangle = w_{\mathrm{target}}$ for each sample, where w_{target} is a prescribed target mean connectivity [18, 19, 20, 21]. In this way, $w \approx 0$ corresponds to series-dominated, bottlenecked regions and $w \approx 1$ to locally well-connected, parallel-like paths.

For the through-plane direction, we adopt a crowding-type throttling law for conduction along the platelet normals. Crowding-factor approaches for non-dilute composites show that particle interactions and reduced accessible matrix volume require a loading-dependent correction beyond dilute EMT, typically entering as a monotone increase in effective resistivity with volume fraction [22]. Consistent with this non-dilute philosophy, but tailored to our voxel-scale uniaxial channel rather than a bulk effective conductivity, we take the local through-plane conductivity of the GNP-rich phase as

$$\kappa_{\perp}(\phi_{\mathrm{GNP}}) = \frac{\kappa_{\perp,\mathrm{base}}}{1 + \gamma\, \phi_{\mathrm{GNP}}}, \tag{18}$$

so that $\kappa_{\perp} \to \kappa_{\perp,\mathrm{base}}$ as $\phi_{\mathrm{GNP}} \to 0$, which is equivalent to assuming an approximately linear growth of platelet-normal thermal resistivity with local GNP crowding. This choice captures the experimentally observed suppression of through-plane transport by platelet stacking/alignment [23] and the increasing role of Kapitza-type interfacial resistance in platelet composites at higher loadings [24, 25]. This GNP-normal response is then mixed with the matrix in the voxel-scale mixture law, ensuring that $\kappa_{zz} \to \kappa_m$ as $\phi_{\mathrm{GNP}} \to 0$, and is capped by an upper limit $\kappa_{zz,\mathrm{cap}}$ to prevent nonphysical growth at high local fractions. Here $\kappa_{\perp,\mathrm{base}}$ sets the nominal through-plane conductivity of well-separated platelets, while γ controls how rapidly this channel is throttled by crowding and interfacial resistance as the local GNP content increases.

Because individual platelet orientations are not resolved at the microCT voxel scale, we adopt a random-orientation approximation and collapse this uniaxial response to an effective isotropic GNP conductivity by averaging the principal values. For a transversely isotropic conductivity tensor with eigenvalues $(\kappa_\parallel, \kappa_\parallel, \kappa_\perp)$, uniform orientation averaging over a randomly oriented ensemble yields an isotropic conductivity equal to one third of the tensor trace, i.e., $\kappa_{\text{iso}} = (2\kappa_\parallel + \kappa_\perp)/3$ [26, 27]:

$$\kappa_{\text{GNP,iso}}(\phi_{\text{GNP}}) = \frac{2\,\kappa_\parallel(\phi_{\text{GNP}}) + \kappa_\perp(\phi_{\text{GNP}})}{3}. \tag{19}$$

This $\kappa_{\text{GNP,iso}}$ is then used in the local mixture law with the paraffin matrix to define the voxel-scale solid-phase conductivity. In this sense, the reported voxel conductivities are already orientation-averaged over an underlying ensemble of randomly oriented platelets.

S4 Specific heat capacity

Figure S6 compares the heating specific heat capacity $C_p(T)$ of neat paraffin and the EG2 composite. For neat paraffin, the baseline heat capacity away from the transition is $\sim 2\ \mathrm{J\,g^{-1}\,K^{-1}}$, with $C_p = 2.21\ \mathrm{J\,g^{-1}\,K^{-1}}$ at 35.5 °C. The apparent C_p increases sharply through the melting interval and reaches a maximum of $\sim 19.6\ \mathrm{J\,g^{-1}\,K^{-1}}$ at 67.5 °C, consistent with reported modulated-DSC measurements of paraffin waxes [28]. Relative to neat paraffin, EG2 shows a modest but clear reduction in C_p across the range. At 35.5 °C, C_p decreases to $1.92\ \mathrm{J\,g^{-1}\,K^{-1}}$, corresponding to a 13% reduction. The melting peak height is also reduced, with a maximum of $\sim 17.7\ \mathrm{J\,g^{-1}\,K^{-1}}$ compared with $\sim 19.6\ \mathrm{J\,g^{-1}\,K^{-1}}$ for neat paraffin, consistent with dilution of the PCM by the graphite phase and partial confinement of paraffin near the EG network. Literature on EG–PCM composites likewise reports that C_p decreases with increasing EG content. Zhao et al. report that the liquid-state C_p drops from 2.13 to $1.12\ \mathrm{J\,g^{-1}\,K^{-1}}$ as EG increases from 15 to 25 wt%. This stronger decrease at higher EG loading provides a quantitative benchmark consistent with the smaller reduction we observe at 2 wt% EG [29].

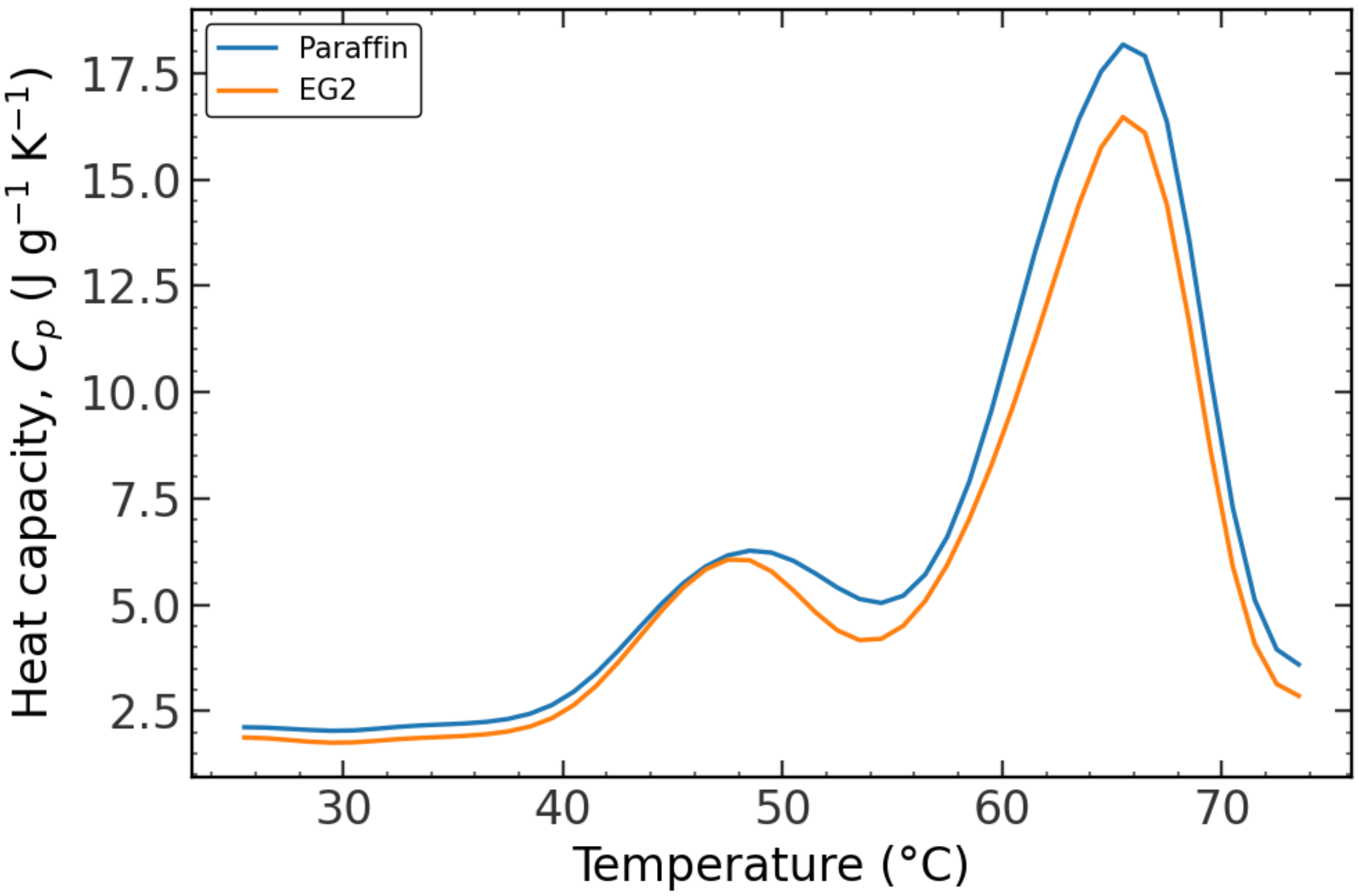

Figure S6: Specific heat capacity C_p as a function of temperature for paraffin and the EG2 composite.

S5 Cyclic Melting–Resolidification Stability

To assess whether the hybrid filler network is retained after repeated phase transitions, sample H2 was subjected to two successive melting–resolidification cycles. In each cycle, the sample was reheated to 90 °C in its original fabrication mold until fully liquid, then cooled to room temperature under ambient conditions and remeasured using the same PPMS-based thermal transport measurement described in Section S2.

The measured thermal conductivities were 1.1 ± 0.1, 1.0 ± 0.1, and 1.1 ± 0.1 $\mathrm{W\,m^{-1}\,K^{-1}}$ for the as-fabricated sample, after cycle 1, and after cycle 2, respectively (Figure S7). All three values agree within experimental uncertainty, with a maximum deviation of approximately 6% observed after the first melting cycle, followed by full recovery to the original value after the second cycle. The modest cycle-to-cycle variation is well within the combined measurement uncertainty and does not indicate a systematic degradation trend.

These results suggest that the composite microstructure, including the EG backbone and the surrounding GNP-enriched matrix, re-forms reliably upon resolidification in a confined mold geometry and that the effective thermal conductivity of the hybrid composite is not degraded by repeated melting–freezing cycles over the range tested.

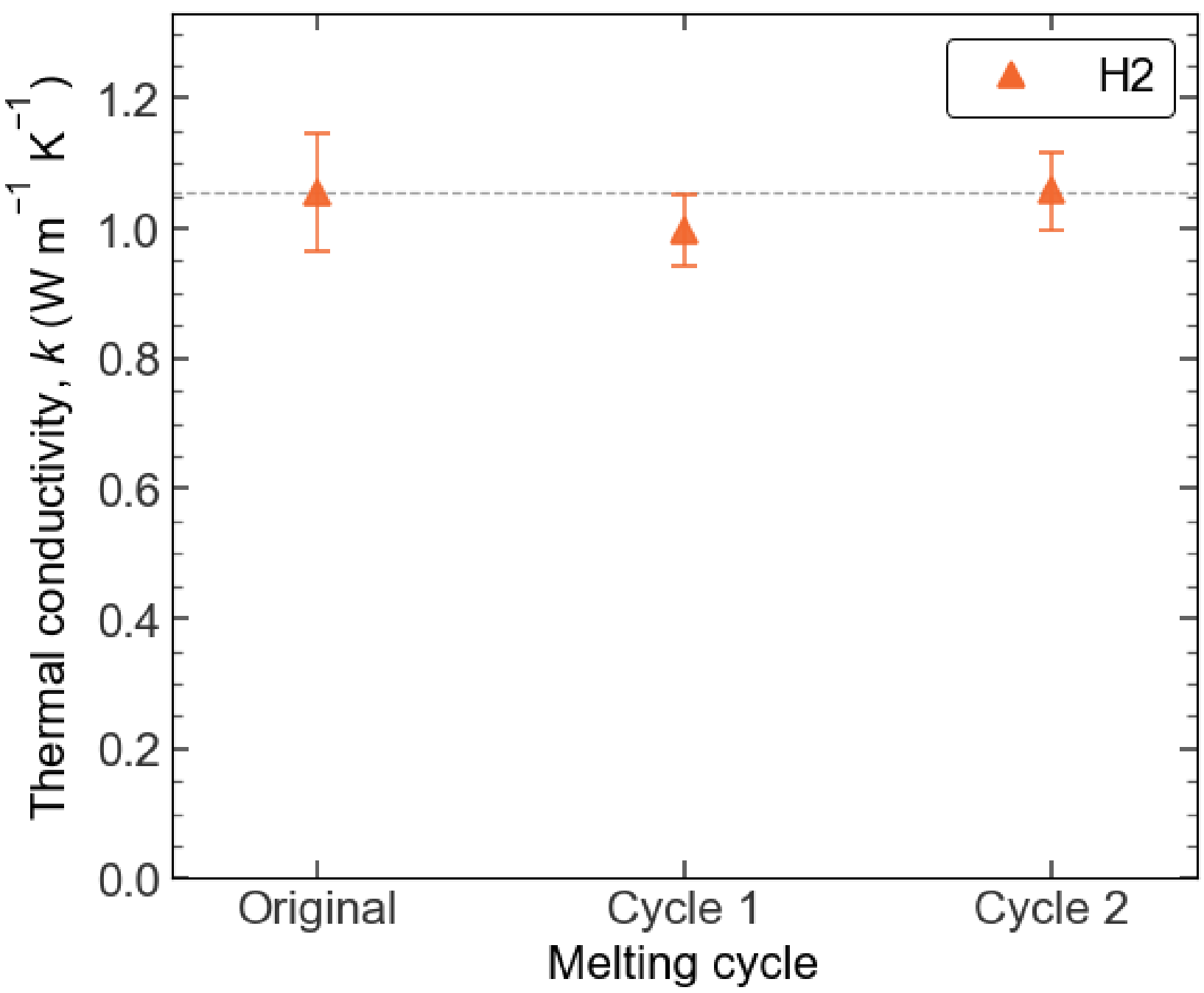

Figure S7: Thermal conductivity of sample H2 before and after two melting–resolidification cycles

S6 Finite-volume conduction solver

Having assigned each interior voxel either a pore, worm, or paraffin+GNP conductivity tensor, we compute the effective macroscopic conductivity by solving the steady-state heat equation on the RVE, following standard digital-microstructure homogenization practice [30]. At the continuum level, the temperature field $T(\mathbf{r})$ satisfies

$$\nabla \cdot \big(\kappa(\mathbf{r})\nabla T(\mathbf{r})\big) = 0, \tag{20}$$

within the interior RVE. The conductivity tensor $\kappa(\mathbf{r})$ is taken as voxelwise constant and diagonal, with jump discontinuities across phase boundaries, and the domain contains no volumetric heat sources [31].
The finite-volume discretization is carried out on the same Cartesian grid as the microCT voxels, restricted to the interior mask so that all control volumes lie fully inside the specimen. In the cell-centered formulation, the governing equation is integrated over each voxel and the divergence theorem converts it to a balance of conductive fluxes across the six voxel faces, yielding a conservative 7-point stencil on the voxel grid. A conservative, cell-centered finite-volume formulation is particularly well suited to this voxelized setting because it operates directly on the native grid, enforces local flux conservation, and avoids remeshing of high-contrast microstructures. Alternative voxel-FEM or FFT solvers would recover the same homogenized limit for a sufficiently resolved, representative RVE, but are less convenient for our trimmed interior domain and mixed boundary conditions [32, 33].
We impose a fixed temperature difference ΔT between two opposite faces of the RVE as a Dirichlet boundary (taken here as the planes at $x = 0$ and $x = L_x$), with

$$T(x=0) = T_{\text{hot}}, \qquad T(x=L_x) = T_{\text{cold}} = T_{\text{hot}} - \Delta T. \tag{21}$$

A schematic of the Dirichlet boundaries across the x-direction of the RVE is shown in Figure S8a. Numerically, these Dirichlet planes are enforced by treating the boundary as a fixed-temperature ghost state located one half-voxel outside the domain, so that the boundary-face conductance uses the appropriate half-cell distance and contributes to the diagonal and right-hand-side of the discrete balance for the boundary-adjacent voxels. The x-direction of the RVE aligns with the temperature-gradient direction of the experimental measurements. The remaining four faces are treated as periodic in the transverse directions, which is a canonical RVE boundary condition that improves convergence to effective properties and suppresses artificial lateral boundary layers for statistically representative media [32, 31]. In practice, periodicity is enforced by wrapping neighbor indices across opposite transverse faces during stencil assembly, so that temperatures are periodic and the outward flux leaving one side re-enters the opposite side with equal magnitude and opposite sign. A schematic of the transverse periodic boundary condition is shown in Figure S8b.

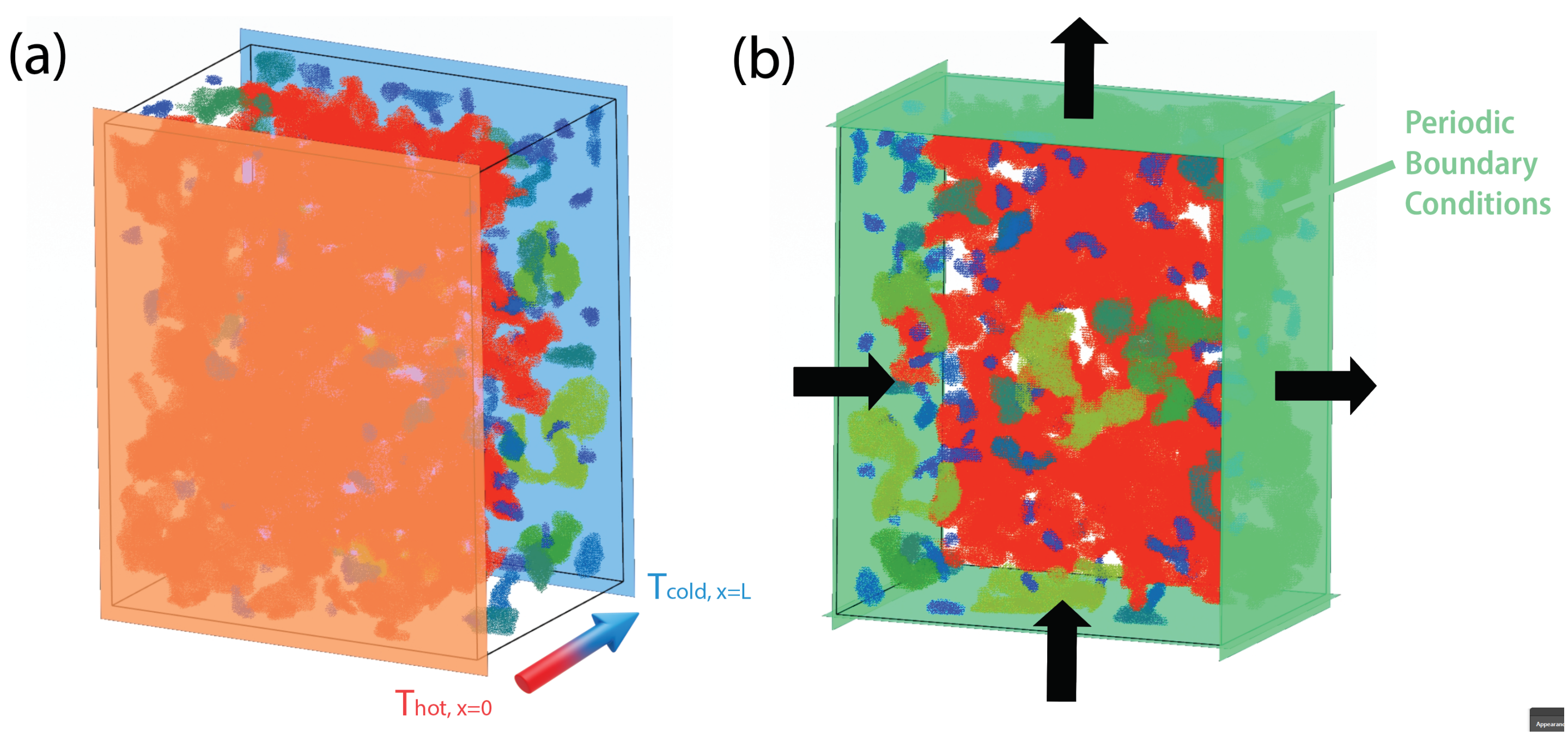

Figure S8: Finite-volume boundary conditions. **(a)** Dirichlet boundary conditions are applied on the x-faces, prescribing T_{hot} at $x = 0$ and T_{cold} at $x = L$ (red/blue) to match the experimental gradient direction. **(b)** Periodic boundary conditions are applied on the transverse faces (y and z; green).

Interfaces between pore, worm, and paraffin+GNP voxels are handled implicitly through the spatial variation of $\kappa(\mathbf{r})$. We do not introduce any additional contact-resistance terms beyond what is already subsumed in the voxel-scale conductivi-

ties. At this voxel scale, the relevant thermal boundary resistances are not independently constrained and would trade off against the inferred κ_{worm} and the calibration of $P(\mathbf{r})$, so the fitted conductivities are interpreted as effective, interface-inclusive parameters. For each interior face, the normal heat flux is written in terms of a face conductance that combines the two neighboring cell conductivities in series over half-voxel distances (equivalently, a harmonic construction along the face normal), which yields physically consistent fluxes for discontinuous media and preserves symmetry for diagonal tensors.

While we choose not to implement it here, the solver includes an optional explicit interfacial thermal resistance $R_{b,ij}$ with units of $\text{m}^2\,\text{K/W}$. Here, i and j denote the material classes of the two voxels sharing a face. For a face of area A_f with unit normal $\mathbf{n}$ and grid spacing Δ along $\mathbf{n}$, the face resistance and conductance are

$$\mathcal{R}_f = \frac{\Delta}{2\kappa_{n,i}} + \frac{\Delta}{2\kappa_{n,j}} + R_{b,ij}, \qquad G_f = \frac{A_f}{\mathcal{R}_f}, \tag{22}$$

where $R_{b,ij}$ is the interfacial thermal boundary resistance between material classes i and j, $\boldsymbol{\kappa}_i$ and $\boldsymbol{\kappa}_j$ are the voxel conductivity tensors, and

$$\kappa_{n,i} = \mathbf{n}^{\mathsf{T}}\boldsymbol{\kappa}_i\mathbf{n}, \qquad \kappa_{n,j} = \mathbf{n}^{\mathsf{T}}\boldsymbol{\kappa}_j\mathbf{n} \tag{23}$$

are their conductivity components along the face normal. This modified conductance G_f is used consistently during stencil assembly and in post-processing of internal and boundary heat fluxes, so that the discrete operator remains symmetric and the resulting fluxes satisfy the same local conservation properties as the base harmonic construction. The resistance can be specified as a single uniform value applied to all dissimilar interfaces, or as a symmetric phase-pair map satisfying $R_{b,ij} = R_{b,ji}$ and $R_{b,ii} = 0$. This interface resistance option is only implemented in the validation test cases that follow in a later section, and exists for general applicability for material systems beyond the one presented in this work.

The governing equation is discretized using a standard cell-centered finite-volume scheme with conservative fluxes across each voxel face. Face conductivities are obtained from neighboring cell tensors using a harmonic, face-normal construction for discontinuous media, so that the resulting discrete operator is symmetric and strictly positive definite [33]. The resulting sparse linear system for the cell-centered temperatures is solved iteratively with a preconditioned Krylov method using conjugate gradient and a diagonal (Jacobi) preconditioner [34]. For the large voxel counts required by microCT RVEs, the solve is accelerated on a GPU by storing the operator in compressed sparse row (CSR) format and executing sparse matrix–vector products and vector updates on-device via CuPy/`cupyx` (CUDA), with a CPU sparse-solver fallback implemented using SciPy when needed [35, 36]. Convergence is assessed using a relative residual tolerance.

Once a steady-state temperature field is obtained, we compute the local heat flux $\mathbf{q}(\mathbf{r}) = -\mathbf{k}(\mathbf{r})\nabla T(\mathbf{r})$ using the same face-consistent gradients employed in the finite-volume balance, and extract the effective conductivity along the imposed gradient direction from the net flux through the hot and cold faces, consistent with standard thermal RVE averaging [31, 32]. Denoting by $\langle q_x \rangle$ the area-averaged normal flux on the $x = 0$ or $x = L_x$ face, the effective conductivity in the x-direction is

$$\kappa_{\text{eff},x} = -\frac{\langle q_x \rangle L_x}{\Delta T}. \tag{24}$$

As a consistency check on the converged solution, the net heat rates through the hot and cold faces are in close agreement, indicating that the discrete fluxes are locally conservative. Because the lateral boundaries are periodic and the microstructure within the RVE is statistically representative, $\kappa_{\text{eff},x}$ can be interpreted as the effective bulk conductivity of that composite along the loading direction [32].

S6.1 Hybrid Microstructure FVM Runtime and Energy Balance

Figure S9 summarizes solver performance for the hybrid κ_{worm} parameter sweep. For each hybrid microCT RVE (H2–H5), we run the finite-volume solver at each candidate κ_{worm} value while holding the matrix–GNP mixture response fixed to the parameters calibrated from the 2 wt% GNP fitting procedure. For every run we record (i) a boundary energy-imbalance metric based on the mismatch between the inlet and outlet heat currents and (ii) the wall-clock runtime on an NVIDIA RTX 4090.

Across all samples and sweep points, the energy imbalance remains extremely small, with distributions clustered at $\sim 10^{-9}$–10^{-8} (Figure S9a). This indicates that the converged discrete solution is effectively source-free and flux-conservative, so the extracted κ_{eff} from the boundary heat flux is not limited by numerical nonconservation. The GPU-accelerated solve is also fast, with typical runtimes well below two minutes per run (Figure S9b). This throughput enables dense κ_{worm} sweeps and large ensembles of accurate simulations directly on multi-million voxel, experimentally reconstructed composite microstructures.

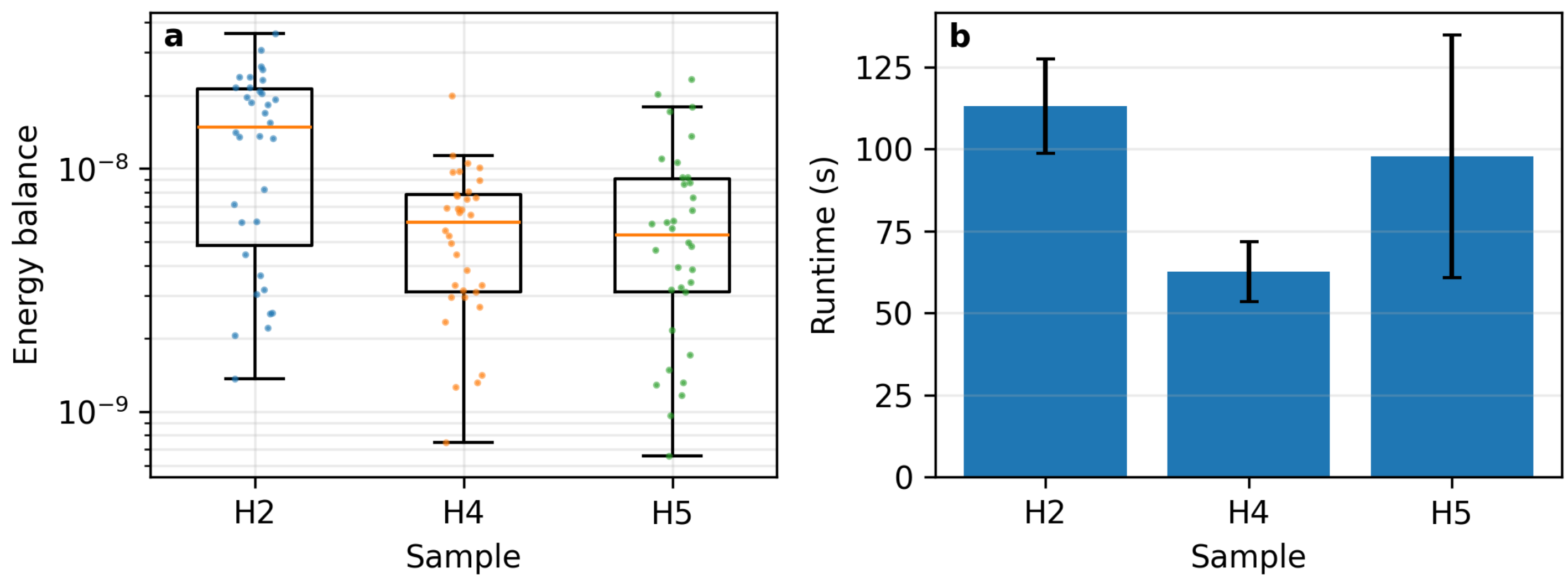

Figure S9: Energy conservation and runtime for hybrid κ_{worm} sweeps. (a) Distribution of the boundary energy-imbalance metric over all κ_{worm} sweep points for each hybrid RVE (H2–H5) using the 2 wt% GNP-calibrated matrix–GNP parameters. (b) Mean runtime per solve on an NVIDIA RTX 4090, with error bars showing the run-to-run variation across the sweep for each sample.

S6.2 Finite-volume Model Validation

To validate the FVM solver, we run a suite of conduction problems on simple voxelized geometries and compare the extracted effective conductivities to analytical references. Table S2 lists the parameters for each case and the corresponding closed-form expressions used for comparison. The homogeneous case admits an exact reference, the laminate and bar cases are compared to series and parallel mixture limits evaluated using the prescribed or realized phase fractions, and the dilute sphere pack is compared to effective-medium predictions with and without an interfacial thermal resistance [25, 37, 38]. Figure S10 summarizes the validation geometries. All cases are posed on a cubic domain of side length $L = 1$ mm with fixed temperatures applied on the two faces normal to the x axis and steady-state solutions computed under periodic conditions on the remaining faces. The suite probes limiting behaviors and challenging contrasts using a homogeneous reference, layered stacks with large property jumps, bar geometries that approach series and parallel bounds, and random spherical inclusions in the dilute limit where effective-medium predictions apply.

Case	N	$\{\kappa_i\}$ (W m^{-1} K^{-1})	Key parameters	Analytical reference
Uniform	20	$\{1\}$	none	$\kappa_{\mathrm{eff},x} = \kappa$
2-layer (1:100)	40	$\{1,\ 100\}$	$f_1 = f_2 = 0.5$	$\kappa_{\mathrm{eff},x}^{-1} = \sum_i f_i/k_i$
2-layer (1:5000)	40	$\{1,\ 5000\}$	$f_1 = f_2 = 0.5$	$\kappa_{\mathrm{eff},x}^{-1} = \sum_i f_i/k_i$
Bars series	32	$\{1,\ 10\}$	$n_{\mathrm{bars}} = 4,\ \phi = \langle I \rangle$	$\kappa_{\mathrm{eff},x}^{-1} = (1-\phi)/k_m + \phi/k_p$
Bars parallel	32	$\{1,\ 10\}$	$n_{\mathrm{bars}} = 4,\ \phi = \langle I \rangle$	$\kappa_{\mathrm{eff},x} = (1-\phi)\, k_m + \phi\, k_p$
Spheres MG	48	$\{\kappa_m,\ \kappa_p\} = \{1,\ 10\}$	$\phi_{\mathrm{target}} = 0.05,\ r_{\mathrm{vox}} = 4$	$\kappa_{\mathrm{eff}} = \kappa_m \frac{1+2A\phi}{1-A\phi}, \quad A = \frac{\kappa_p/\kappa_m - 1}{\kappa_p/\kappa_m + 2}$
Spheres ITR	48	$\{\kappa_m,\ \kappa_p\} = \{1,\ 10\}$	$R_b = 2 \times 10^{-7}$ m^2 K W^{-1}, $a = (L/N) r_{\mathrm{vox}}$	$\kappa_{\mathrm{eff}} = \kappa_m \frac{1+2A\phi}{1-A\phi}, \quad A = \frac{1-\beta}{1+2\beta}, \quad \beta = \frac{\kappa_m}{\kappa_p} + \frac{R_b k_m}{a}$
Multi-layer	40	$\{1,\ 0.5,\ 2\}$	$(0, 1, 2, 1)$ with $f_i = 0.25$	$\kappa_{\mathrm{eff},x}^{-1} = \sum_i f_i/\kappa_i$

Table S2: Validation case parameters and analytical references. All cases use $L = W = H = 1$ mm with $T_{\mathrm{hot}} = 310$ K and $T_{\mathrm{cold}} = 290$ K applied on faces normal to x, and periodic boundary conditions on the remaining faces. Here κ_m and κ_p denote matrix and inclusion conductivities, ϕ is the realized inclusion volume fraction from the voxel mask, and N is the voxel resolution (number of voxels per cube edge, so the voxel size is L/N). MG denotes the Maxwell–Garnett effective-medium form and ITR includes interfacial thermal resistance R_b, with a the inclusion radius. Analytical reference forms for the layered and bar geometries follow standard series/parallel mixture rules [39], while the MG and ITR sphere expressions follow Nan *et al.* [24].

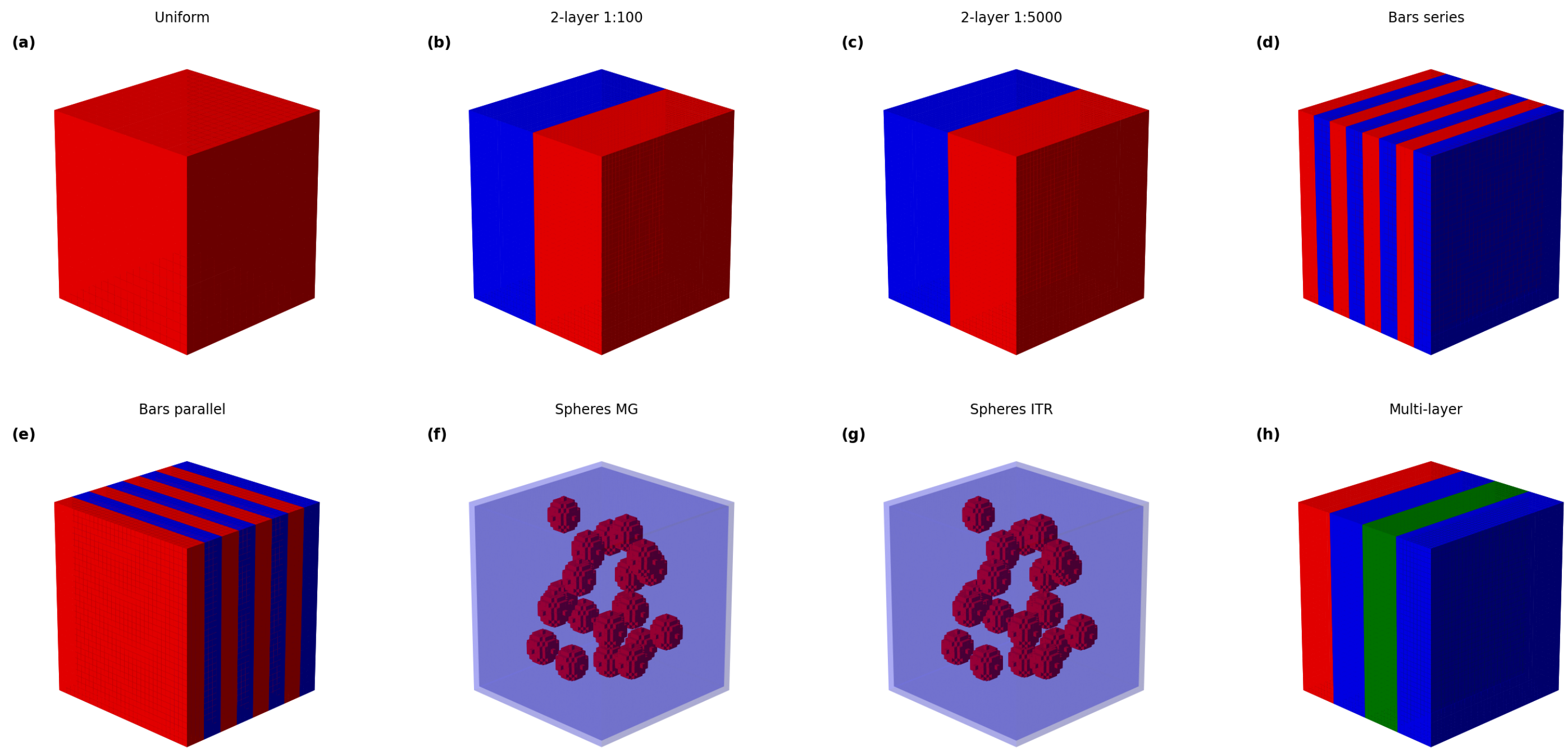

Figure S10: Validation geometries used to benchmark the finite-volume conduction solver across analytic limits and high-contrast cases. (a) Uniform single-phase cube. (b,c) Two-layer laminates with conductivity contrast ratios of 1:100 and 1:5000 (blue:red), oriented to probe the series response along the imposed conduction direction. (d,e) Alternating bar geometries arranged to enforce (d) series and (e) parallel bounds. (f) Random spherical inclusions (red) in a low-conductivity matrix (blue; rendered semi-transparent for visibility), compared to the Maxwell–Garnett effective-medium limit. (g) Same sphere geometry with an imposed interfacial thermal resistance (ITR), compared to the Hasselman–Johnson composite-sphere model. (h) Three-layer laminate (red/blue/green) used to validate multi-phase handling at high contrast.

Figure S11 reports the converged temperature field on an XY mid-slice for each validation case under identical boundary temperatures, with cases and analytical references summarized in Table S2. The uniform case exhibits an essentially linear gradient along the conduction axis, which is the expected solution for a homogeneous medium. In the two-layer cases, the temperature drop concentrates within the low-conductivity layer while the high-conductivity layer remains nearly isothermal, and this behavior persists at the highest contrast, which is consistent with continuity of normal heat flux across the interface. The bar cases produce gradients that reflect their effective series and parallel orientations, with stronger gradient localization in the series configuration and a more uniform macroscopic gradient in the parallel configuration. For the dilute sphere pack, the temperature field remains close to the uniform solution with smooth, weak perturbations around inclusions, while the added interfacial resistance increases the local distortion without introducing nonphysical oscillations. The multi-layer case shows slope changes aligned with the prescribed layer sequence, which is consistent with piecewise

constant conductivity and steady one-dimensional conduction in each layer.

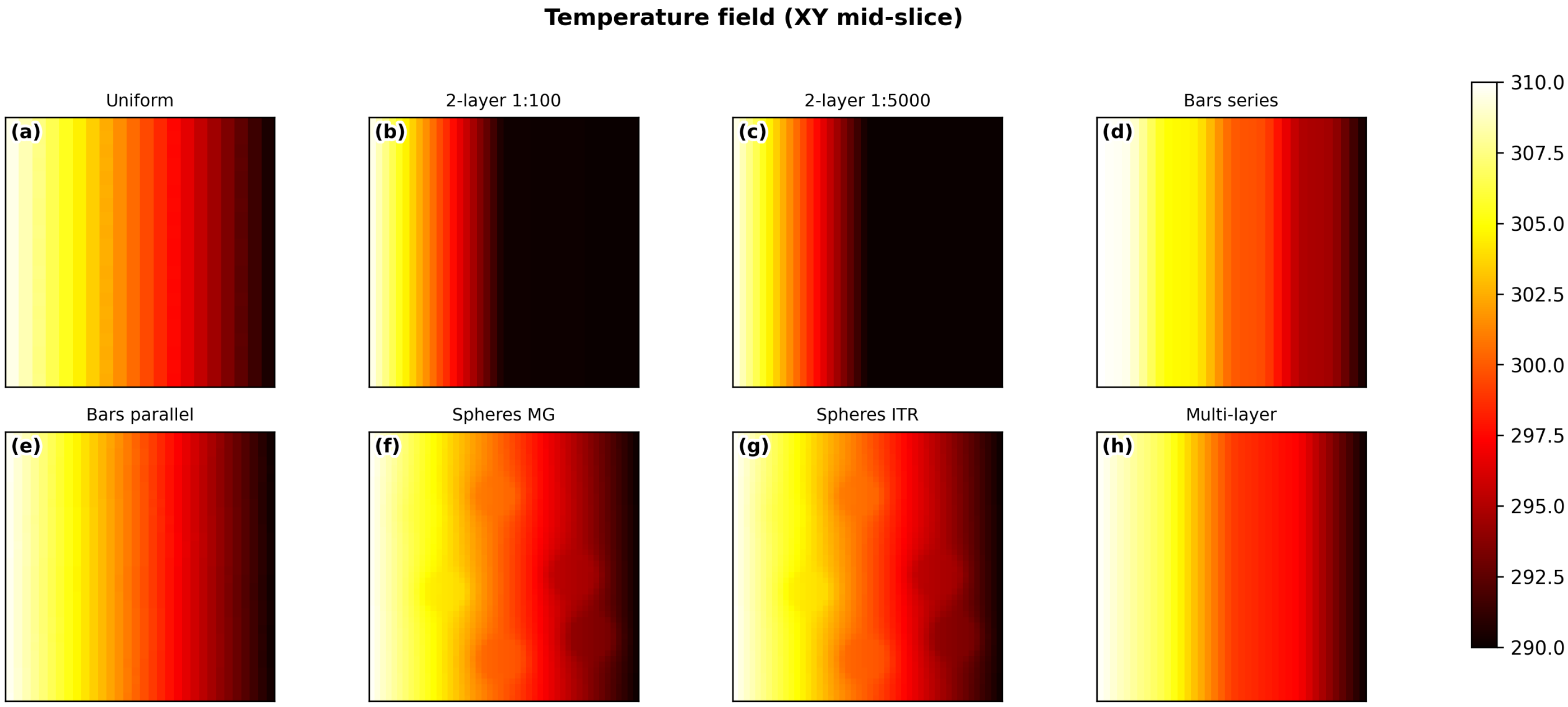

Figure S11: Converged steady-state temperature T on the XY mid-plane for each validation geometry under $T_{\text{hot}} = 310$ K and $T_{\text{cold}} = 290$ K applied on the faces normal to x. Panels (a) through (h) correspond to the cases listed in Table S2.

Figure S12 reduces each three-dimensional solution to a one-dimensional diagnostic by plotting the yz-averaged temperature profile $\langle T \rangle(x)$. The uniform case is nearly perfectly linear, which confirms the expected response for a homogeneous medium. In the two-layer cases, the profile becomes strongly piecewise linear and the temperature drop localizes almost entirely within the low-conductivity half, while the high-conductivity half is nearly isothermal. The bar series case exhibits multiple slope changes that track the alternating segments along the conduction direction, whereas the bar parallel case remains close to linear because the conductivity pattern does not vary along x and the cross-section provides parallel pathways at each slice. For the dilute sphere pack, $\langle T \rangle(x)$ stays close to linear with only smooth deviations, and the added interfacial resistance increases the deviation modestly without introducing nonphysical oscillations. The multi-layer case shows clear slope changes aligned with the layer sequence, consistent with steady one-dimensional conduction within each layer and continuity of heat current across interfaces.

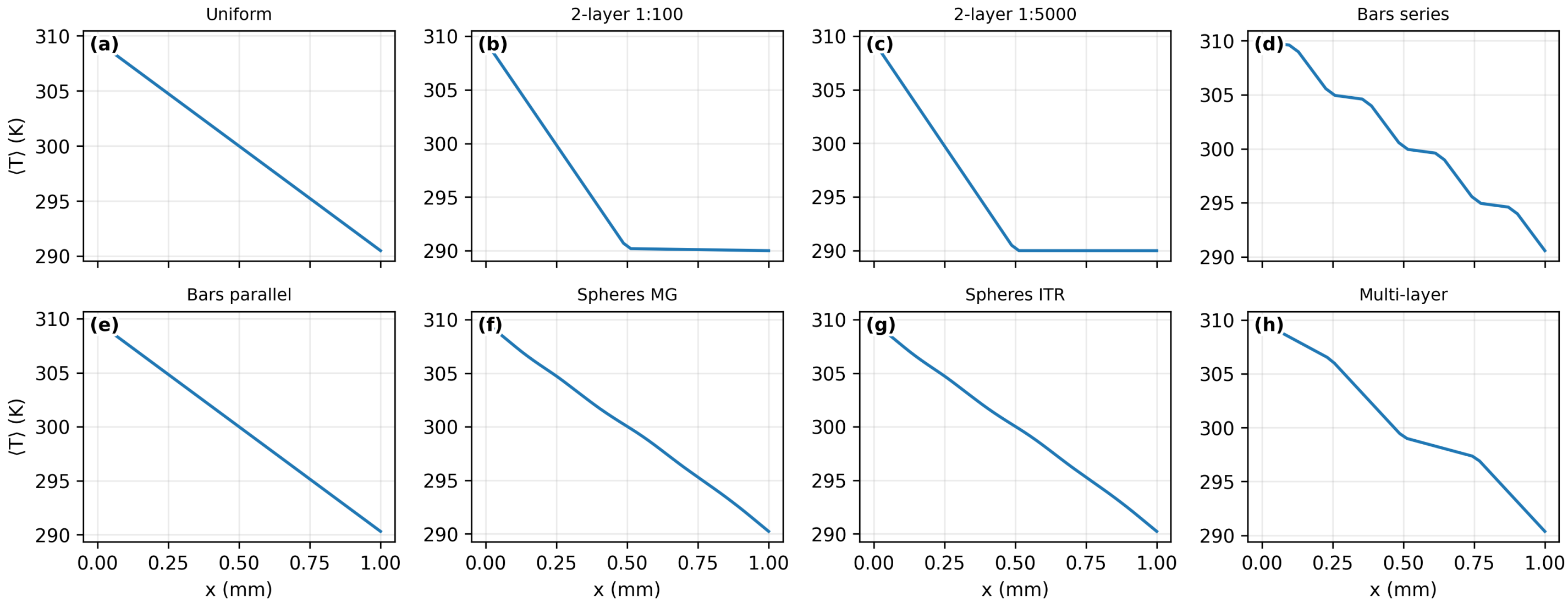

Figure S12: yz-averaged temperature profiles for the FVM validation suite. For each validation case listed in Table S2, the converged steady-state solution is reduced to a one-dimensional diagnostic by averaging the cell-centered temperature over each yz plane to obtain $\langle T \rangle(x)$. The uniform case yields an approximately linear profile, while layered and bar geometries exhibit slope changes that reflect their imposed spatial variations in conductivity along the conduction axis. The sphere cases remain close to linear with smooth deviations that arise from the realized inclusion distribution and, when present, added interfacial thermal resistance.

Figure S13 validates the finite-volume solver using closed-form benchmarks and a global conservation check. As shown in Figure S13**a**, the numerical results lie on the $1:1$ line to within the plotting symbols across all cases. Figure S13**b** reports the boundary energy imbalance, $\epsilon_{\mathrm{bc}} = \left|Q_{\mathrm{hot}} + Q_{\mathrm{cold}}\right| / \langle |Q| \rangle \times 100\%$, which remains extremely small for the full contrast range, at $\mathcal{O}(10^{-9}\text{–}10^{-5})\%$.

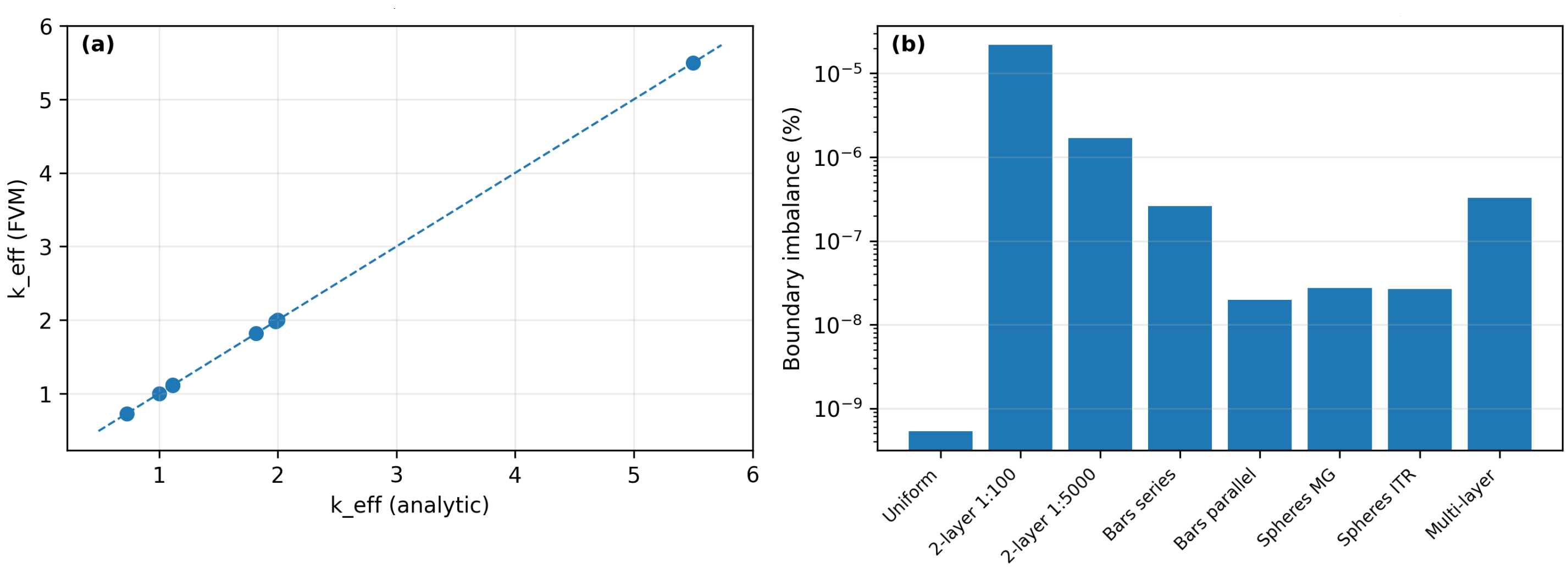

Figure S13: Analytical agreement and boundary energy balance. (a) Parity plot comparing κ_{eff} extracted from the FVM boundary heat flux to analytical predictions for the full validation suite; the dashed line indicates perfect agreement. **(b)** Boundary energy imbalance $\epsilon_{\mathrm{bc}} = \left|Q_{\mathrm{hot}} + Q_{\mathrm{cold}}\right| / \langle |Q| \rangle \times 100\%$ for each case (log scale), showing vanishing net-flux error even for high-contrast layered geometries.

Validation against a literature diamond/Cu composite

In addition to the closed-form benchmarks reported above, we further validate the FVM solver using a literature diamond/Cu composite with a complex, realistic microstructure, beyond idealized analytical benchmarks. Chang *et al.* report a peak composite thermal conductivity of $\kappa \approx 763\ \mathrm{W\,m^{-1}\,K^{-1}}$ at a reduced diamond volume fraction of $V_d = 45\%$ and an average particle radius $a = 115\ \mu$m, and interpret the results using a differential effective medium (DEM) model with an interfacial thermal conductance parameter G [40]. Their DEM analysis deduces an effective diamond–Cu interfacial conductance

of $G \approx 93.5\ \mathrm{MW\,m^{-2}\,K^{-1}}$ from the measured composite conductivities. This system provides a stringent numerical test because it combines a high conductivity contrast, a dense inclusion fraction beyond the dilute regime, and sensitivity to interface-limited transport captured through G.

We reconstruct a statistically similar 3D digital microstructure using the microCT cross-sections shown in Fig. 3 of Chang *et al.* as a quantitative reference. The empirical particle size distribution is extracted from three orthogonal microCT slices via watershed segmentation. A cylindrical volume matching the specimen geometry is populated using a dense random sphere packer that samples radii directly from the measured distribution at the reported diamond volume fraction of $V_d = 0.45$. The packing algorithm employs spatial hashing for efficient neighbor lookup and a three-mode placement strategy combining random insertion, near-contact placement to reproduce particle clustering, and preferred-distance placement to capture the characteristic nearest-neighbor spacing observed in the reference microstructure. Figure S14 shows 2D slices extracted from the synthesized cylinder alongside the corresponding microCT references. The resulting 3D inclusion mask defines the fixed geometry used for all subsequent FVM thermal analyses regarding the Cu-Diamond composites.

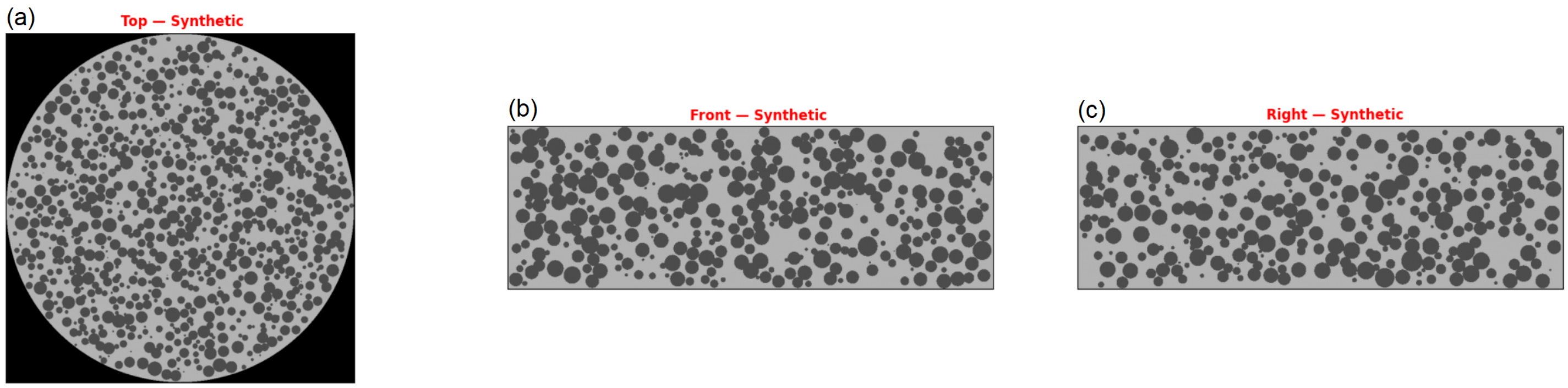

Figure S14: Slice-matched digital diamond/Cu microstructure. Representative orthogonal mid-plane slices through the synthesized 3D diamond-in-copper geometry used for the literature benchmark: (a) xy (top), (b) xz (front), and (c) yz (right). Dark regions indicate diamond inclusions and light regions indicate the Cu matrix. The inclusion size distribution and dense packing character were tuned to qualitatively reproduce the particle cross-sections and spacing observed in the reference microCT images at $V_d \approx 0.45$. [40]

Figure S15 provides a 3D rendering of the corresponding inclusion mask, confirming that the packing character observed in the slice views is representative of the full volume used for homogenization. To quantify the fidelity of the reconstruction, the two-point correlation function $S_2(r)$ is computed for three orthogonal cross-sections extracted from the synthesized cylinder and compared against the corresponding experimental micro-CT slices. The reference particle phase is identified by thresholding each micro-CT image at the intensity value yielding 45% area fraction, consistent with the reported diamond loading. Figure S16 overlays the experimental and synthetic $S_2(r)$ curves for the top, front, and right views, showing close agreement across all length scales with RMSE values of 0.011, 0.024, and 0.018 respectively. These residuals correspond to less than 4% relative deviation on the S_2 scale, confirming that the synthesized packing reproduces the spatial statistics of the reference microstructure, including particle sizes, area fractions, and interparticle spacing, within a single consistent 3D volume.

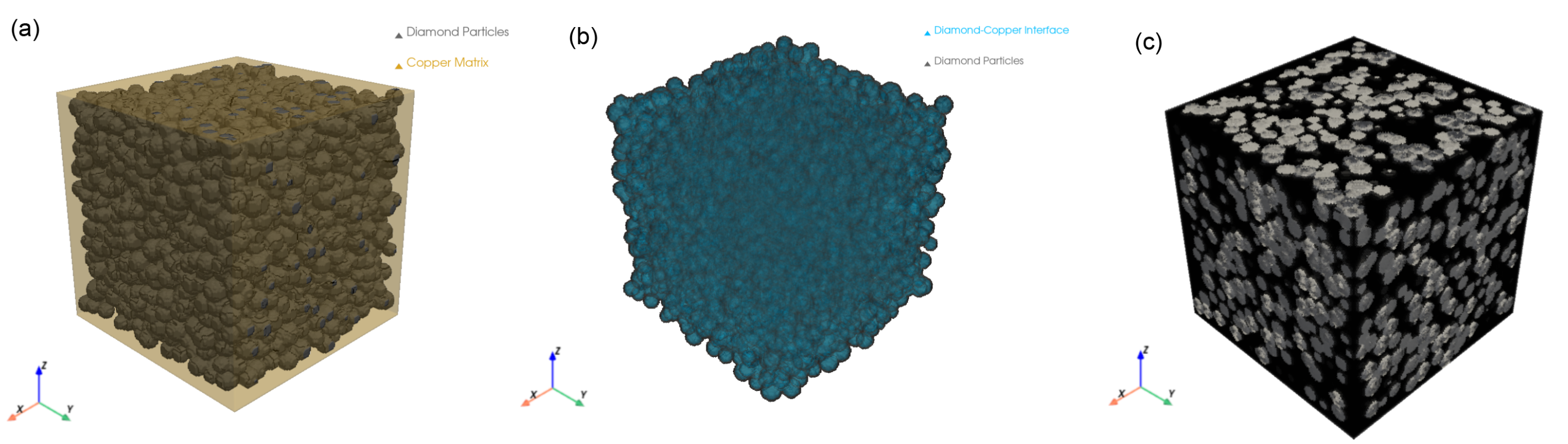

Figure S15: 3D views of the digital diamond/Cu recreated microstructure. (a) Two-phase rendering of the packed diamond inclusions in the Cu matrix. (b) Diamond phase with the diamond–Cu interface highlighted. (c) Grayscale volume view of the voxelized geometry used as input to the finite-volume solver. [40]

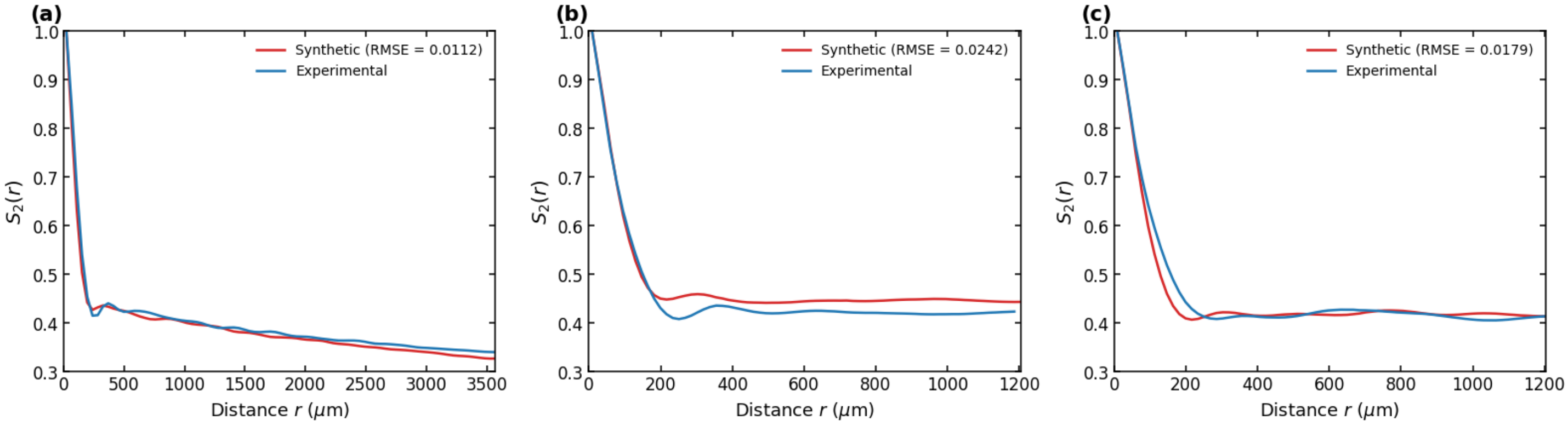

Figure S16: Two-point correlation functions $S_2(r)$ comparing the synthetic microstructure (red) against experimental micro-CT data (blue) for three orthogonal cross-sections of the Cu–diamond composite. RMSE values are indicated in each panel.

The synthesized Cu/diamond microstructure is converted to a two-phase voxel grid by thresholding the intensity field, with voxels above the threshold assigned as copper matrix and voxels below assigned as diamond inclusions. Each voxel is then assigned an isotropic thermal conductivity based on its phase, taking $\kappa_{\mathrm{Cu}} = 398\ \mathrm{W\,m^{-1}\,K^{-1}}$ and $\kappa_{\mathrm{dia}} = 1800\ \mathrm{W\,m^{-1}\,K^{-1}}$. A diamond–copper thermal boundary resistance R_b is applied at phase interfaces. The corresponding interfacial thermal conductance is defined as $G = 1/R_b$. A parametric sweep is performed from perfect contact through strongly resistive interfaces using $R_b \in \{0,\ 10^{-9},\ 3.33\times10^{-9},\ 10^{-8},\ 3\times10^{-8},\ 10^{-7}\}\ \mathrm{m^2\,K\,W^{-1}}$. All cases are posed on the same cubic domain used for the synthetic packing, with Dirichlet temperatures $T_{\mathrm{hot}} = 310$ K and $T_{\mathrm{cold}} = 290$ K applied on the two faces normal to the x axis and periodic boundary conditions on the transverse faces.

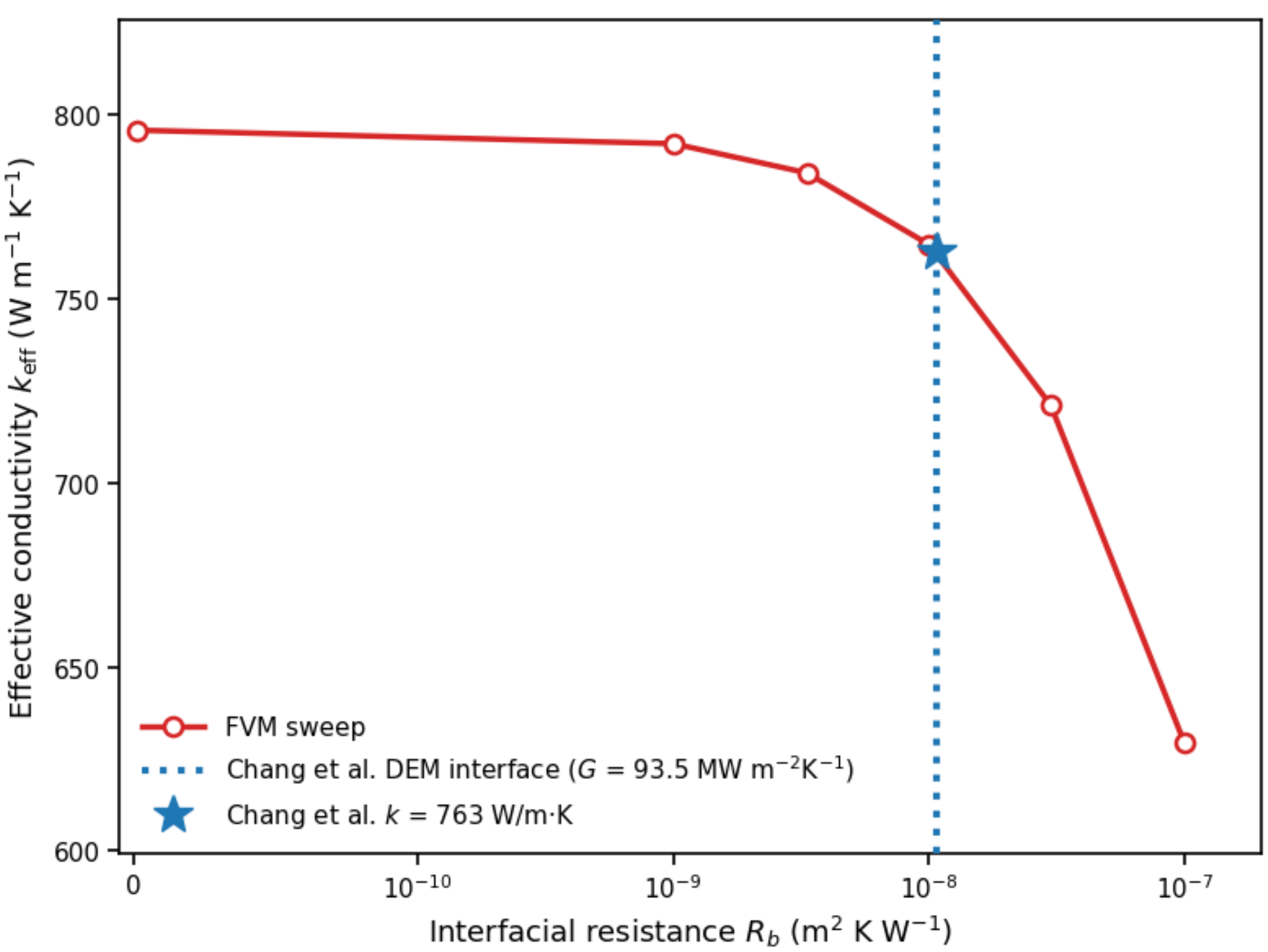

Figure S17: Cu/diamond benchmark: κ_{eff} sensitivity to interfacial resistance. Effective conductivity predicted by the finite-volume model for the digital Cu/diamond microstructure as a function of diamond–Cu thermal boundary resistance R_b. The dashed line indicates the interfacial conductance reported by Chang *et al.* converted to $R_b = 1/G$, and the star marks their measured composite conductivity. [40]

We solve the same steady-state conduction problem as in the benchmarks above. For each interfacial resistance R_b in the sweep, we compute $\kappa_{\mathrm{eff},x}$ and report auxiliary diagnostics that summarize how heat partitions between phases, including a flux-concentration factor defined from the mean flux magnitude in the diamond and copper voxels. Figure S17 reports the finite-volume prediction of the effective conductivity of the slice-matched Cu/diamond microstructure as the

diamond–Cu boundary resistance is increased from perfect contact to strongly resistive interfaces. Over the simulated range, κ_{eff} decreases monotonically from 795.9 $\mathrm{W\,m^{-1}\,K^{-1}}$ at $R_b = 0$ to 629.4 $\mathrm{W\,m^{-1}\,K^{-1}}$ at $R_b = 10^{-7}\ \mathrm{m^2\,K\,W^{-1}}$, demonstrating the expected transition from matrix/particle-limited transport to an interface-influenced regime. Chang *et al.* deduced an interfacial conductance of $G = 93.5\ \mathrm{MW\,m^{-2}\,K^{-1}}$ from their DEM analysis, corresponding to $R_b = 1/G \approx 1.07 \times 10^{-8}\ \mathrm{m^2\,K\,W^{-1}}$ [40]. Using this literature-reported interface value, the recreated microstructure yields $\kappa_{\text{eff}} = 765\ \mathrm{W\,m^{-1}\,K^{-1}}$, within 0.2% of the reported 763 $\mathrm{W\,m^{-1}\,K^{-1}}$.

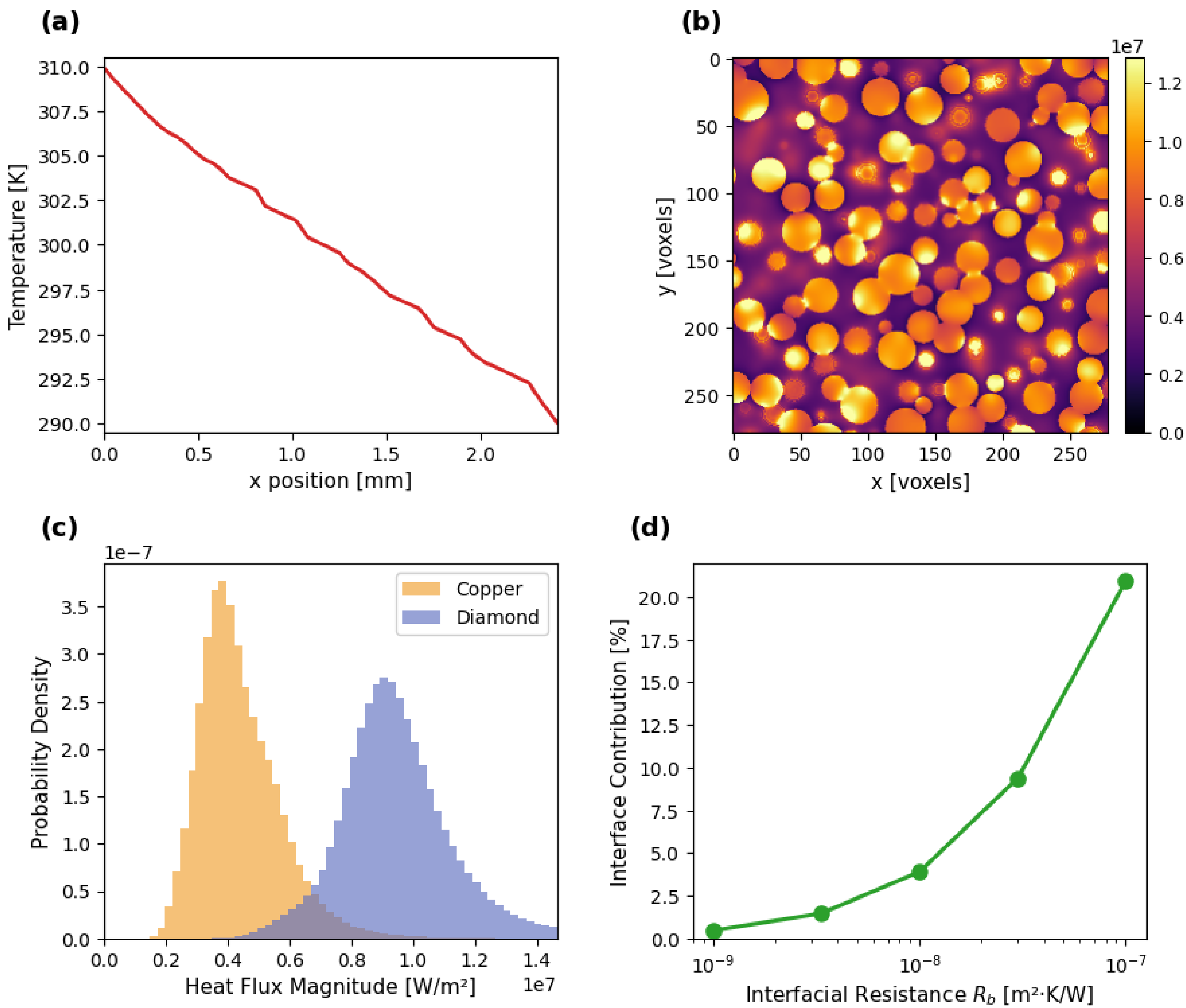

Figure S18: (a) Representative 1D temperature profile along the conduction axis. (b) Example mid-plane slice showing the spatial distribution of heat flux magnitude. (c) Phase-resolved distributions of heat-flux magnitude in the Cu matrix and diamond inclusions. (d) Fraction of the domain-scale resistance attributed to interface penalties as a function of interfacial resistance R_b.

Figure S18 summarizes diagnostics that connect the macroscopic κ_{eff} trends to the underlying transport fields. The temperature profile along the conduction axis in Figure S18a is nearly linear, with only weak local departures from linearity, which is consistent with a converged steady-state solution on a heterogeneous medium. Figure S18b shows that the heat-flux magnitude is spatially intermittent, with elevated flux corridors that track locally favorable through-paths set by the dense particle packing and the surrounding Cu ligaments. This phase selectivity is quantified in the flux histograms in Figure S18c, where diamond voxels exhibit a distribution shifted to higher $|\mathbf{q}|$ relative to the Cu matrix, reflecting preferential current-carrying through the high-conductivity inclusions while the matrix sustains connectivity between particle cross-sections. Finally, panel (d) reports the fraction of the domain-scale resistance attributed to interface penalties, defined as $[R_{\text{tot}}(R_b) - R_{\text{tot}}(0)]/R_{\text{tot}}(R_b)$. This contribution grows monotonically with R_b, rising from about 1% at $10^{-9}\ \mathrm{m^2\,K\,W^{-1}}$ to

about 30% at 10^{-7} $m^2\,K\,W^{-1}$, which explains the increasingly strong suppression of κ_{eff} at large interfacial resistance and confirms that the sweep spans both bulk-dominated and interface-influenced regimes.

The analytical validation suite and the Cu diamond benchmark demonstrate that the FVM remains accurate for both idealized voxel geometries with closed-form references and dense high-contrast microstructures representative of real composites. In the Cu diamond case, a slice matched, microCT inspired reconstruction combined with an interfacial resistance sweep yields effective conductivities consistent with the literature reported interface conductance and measured composite thermal conductivity. This benchmark also verifies the correct use of the built in interfacial thermal boundary resistance capability through the R_b sweep, even though the present paraffin based study does not require an explicit R_b term. These tests highlight the versatility of the framework and support its applicability to heterogeneous composites beyond the systems presented in this work.

S7 Coated platelet calculation for the graphene–paraffin interface resistance

The FVM calibration of the GNP-only composites yields an effective in–plane conductivity of the GNP-rich phase $\kappa_{\parallel,\mathrm{nom}} = 192.7\ \mathrm{W\,m^{-1}\,K^{-1}}$ and a base value for the cross–plane component $\kappa_{\perp,\mathrm{base}} = 4.75\ \mathrm{W\,m^{-1}\,K^{-1}}$. As a consistency check, we verify that the calibrated $\kappa_{\parallel,\mathrm{nom}}$ is compatible with plausible intrinsic properties of graphene nanoplatelets and a finite graphene–paraffin Kapitza resistance using an effective medium theory (EMT) model for GNP–polymer composites [24, 41]. This calculation is not intended as a microscopic measurement of the interface conductance, but rather as an order-of-magnitude sanity check that the fitted transport parameters are physically reasonable within a standard coated-inclusion parameterization.

We approximate each nanoplatelet as an oblate spheroid with semi–axes $a = L_{\mathrm{GNP}}/2$ in plane and $c = t_{\mathrm{GNP}}/2$ through the thickness, with $t_{\mathrm{GNP}} = 11$ nm and $L_{\mathrm{GNP}} = 15\ \mu$m. The intrinsic conductivities of the graphite core are taken as $\kappa_{1,\mathrm{bulk}} = 3000\ \mathrm{W\,m^{-1}\,K^{-1}}$ in the platelet plane and $\kappa_{3,\mathrm{bulk}} = 6\ \mathrm{W\,m^{-1}\,K^{-1}}$ normal to the plane. All GNP properties here are taken to be the manufacturer specifications (SkySpring Nanomaterials). The core is surrounded by an isotropic interphase of thickness $h = 1$ nm with conductivity κ_{int}, which represents the graphene–paraffin interface, and the entire coated particle is embedded in paraffin. The interphase conductivity is related to the thermal boundary resistance R_{bd} through

$$\kappa_{\mathrm{int}} = \frac{d}{R_{\mathrm{bd}}}, \tag{25}$$

where we set $d = h = 1$ nm as an interphase thickness used to parameterize the Kapitza resistance. With this convention, R_{bd} should be interpreted as an effective boundary resistance within the coated-layer model, and its numerical value depends on the adopted geometric and intrinsic-property assumptions.

For an oblate spheroid with aspect ratio

$$\alpha = \frac{t_{\mathrm{GNP}}}{L_{\mathrm{GNP}}} = \frac{c}{a} \quad (0 < \alpha < 1), \tag{26}$$

the depolarization factor in the platelet plane is

$$S_{11}(\alpha) = \frac{\alpha}{2\,(1-\alpha^2)^{3/2}} \left[\arccos(\alpha) - \alpha\sqrt{1-\alpha^2}\right], \tag{27}$$

and the normal component satisfies

$$S_{33} = 1 - 2S_{11}. \tag{28}$$

The interphase occupies the volume between the core with semi–axes (a, c) and the outer coated particle with semi–axes $(a + h, c + h)$. The corresponding interphase volume fraction within the coated inclusion is

$$c_{\mathrm{int}} = 1 - \frac{a^2 c}{(a+h)^2(c+h)} = 1 - \frac{(L_{\mathrm{GNP}}/2)^2(t_{\mathrm{GNP}}/2)}{(L_{\mathrm{GNP}}/2+h)^2(t_{\mathrm{GNP}}/2+h)}. \tag{29}$$

The directional effective conductivity of the coated particle in one principal direction with depolarization factor S and core conductivity κ_{bulk} is written as

$$\kappa_{\mathrm{dir}} = \kappa_{\mathrm{int}} \left[1 + \frac{(1-c_{\mathrm{int}})\,(\kappa_{\mathrm{bulk}} - \kappa_{\mathrm{int}})}{c_{\mathrm{int}} S\,(\kappa_{\mathrm{bulk}} - \kappa_{\mathrm{int}}) + \kappa_{\mathrm{int}}}\right]. \tag{30}$$

We evaluate (30) with $S = S_{11}$ and $\kappa_{\mathrm{bulk}} = \kappa_{1,\mathrm{bulk}}$ to obtain the in–plane effective conductivity $\kappa_{1,\mathrm{FM}}(R_{\mathrm{bd}})$ of the coated platelet, and with $S = S_{33}$ and $\kappa_{\mathrm{bulk}} = \kappa_{3,\mathrm{bulk}}$ to obtain the normal component $\kappa_{3,\mathrm{FM}}(R_{\mathrm{bd}})$.

To estimate the graphene–paraffin boundary resistance within this model, we fit the in–plane direction only, choosing R_{bd} to minimize the squared relative misfit between $\kappa_{1,\mathrm{FM}}(R_{\mathrm{bd}})$ and the calibrated value $\kappa_{\parallel,\mathrm{nom}}$,

$$E(R_{\mathrm{bd}}) = \left[\frac{\kappa_{1,\mathrm{FM}}(R_{\mathrm{bd}}) - \kappa_{\parallel,\mathrm{nom}}}{\kappa_{\parallel,\mathrm{nom}}}\right]^2. \tag{31}$$

The optimum is $R_{\mathrm{bd}} \approx 4.4 \times 10^{-8}\ \mathrm{m^2\,K\,W^{-1}}$, for which the coated–inclusion model predicts $\kappa_{1,\mathrm{FM}} \approx 194\ \mathrm{W\,m^{-1}\,K^{-1}}$, in excellent agreement with $\kappa_{\parallel,\mathrm{nom}}$, shown in Figure S19a. Using the same parameters in (30) with S_{33} and $\kappa_{3,\mathrm{bulk}}$ gives $\kappa_{3,\mathrm{FM}} \approx 0.14\ \mathrm{W\,m^{-1}\,K^{-1}}$, which is much smaller than the calibrated $\kappa_{\perp,\mathrm{base}}$, shown in Figure S19b. This shortfall is expected because the coated single–platelet model does not capture voxel–scale stacking, overlap, and GNP–GNP network formation that can enhance cross–plane conduction in the effective GNP-rich phase. Accordingly, this check is not expected to reproduce $\kappa_{\perp,\mathrm{base}}$, which is an emergent parameter of the image-based, voxel-scale GNP-rich phase rather than a single-platelet property.

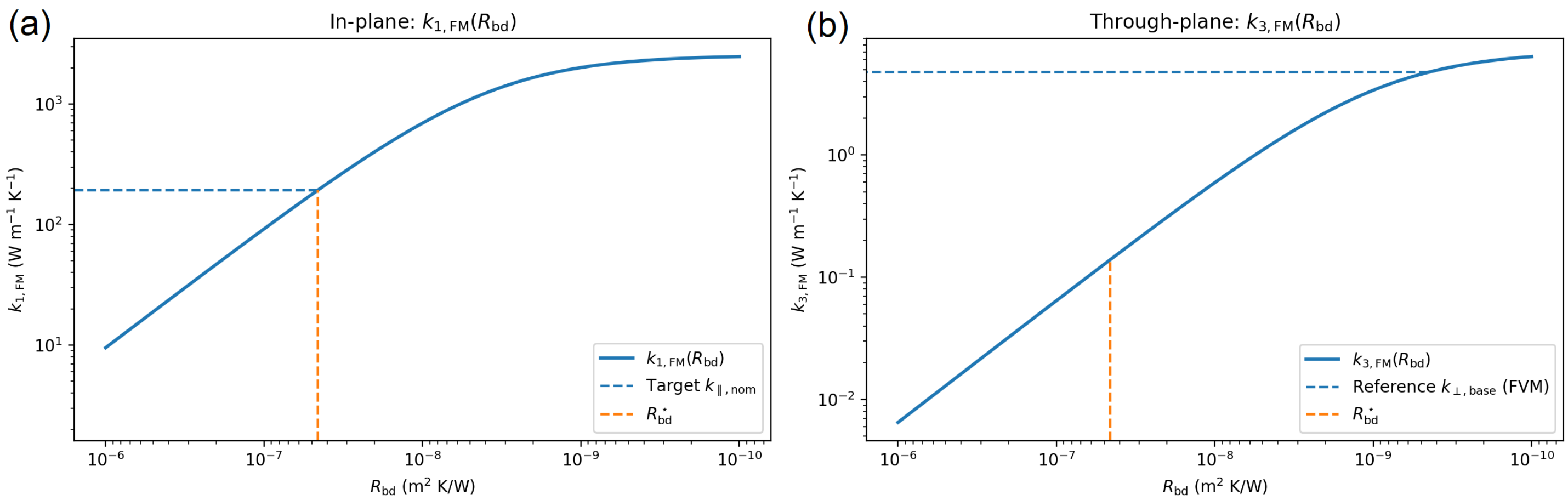

Figure S19: Coated-platelet consistency check for the graphene–paraffin interface resistance. Directional effective conductivities of a single coated oblate spheroid, computed from effective medium theory [24, 41] as functions of thermal boundary resistance R_{bd}. (**a**) In-plane response $\kappa_{1,\mathrm{FM}}(R_{\mathrm{bd}})$, where the dashed horizontal line indicates the FVM-calibrated in-plane target $\kappa_{\parallel,\mathrm{nom}}$ for the effective GNP-rich phase. (**b**) Through-plane response $\kappa_{3,\mathrm{FM}}(R_{\mathrm{bd}})$. The dashed vertical line marks the in-plane best-fit resistance $R^\star_{\mathrm{bd}}$ obtained by matching $\kappa_{1,\mathrm{FM}}$ to $\kappa_{\parallel,\mathrm{nom}}$.

We therefore interpret the fitted R_{bd} as an effective graphene–paraffin boundary resistance inferred from the in–plane attenuation required to reconcile $\kappa_{\parallel,\mathrm{nom}}$ with the assumed intrinsic GNP properties. In terms of interfacial conductance, the fitted value corresponds to $G_{\mathrm{eff}} = 1/R_{\mathrm{bd}} \approx 22.7\ \mathrm{MW\,m^{-2}\,K^{-1}}$. This magnitude is within the broad range reported for graphene–polymer interfaces and is consistent with (though not a direct validation of) interfacial conductances obtained from molecular dynamics simulations for related graphene/paraffin systems [42] and other coated-inclusion analyses [41]. Taken together, the coated-platelet calculation supports the conclusion that the calibrated $\kappa_{\parallel,\mathrm{nom}}$ can be reconciled with physically reasonable Kapitza resistances without introducing additional free parameters into the main image–based modeling framework.

S8 Fitting and Calibration

S8.1 Parameter calibration and joint fitting

The image-based FVM contains no adjustable geometric parameters. Once the pore mask, EG mask, and soft-GNP field are extracted from the microCT data, the voxel-level phase arrangement is fixed. Calibration is therefore confined to constitutive parameters describing how unresolved GNP platelets modify the local paraffin conductivity and how the soft-GNP field is mapped to effective local GNP loading and connectivity. We separate these into (i) shared physics parameters, collected in $\boldsymbol{\theta}_{\text{phys}}$, that are held common across all GNP-containing composites, and (ii) a small set of per-scan image-to-property factors $\boldsymbol{\theta}_{\text{img},j}$ that account for modest scan-to-scan variations in contrast while leaving the microCT-derived geometry unchanged. The shared parameters govern the GNP mixture laws and logistic connectivity mapping (including nominal in-plane and through-plane responses and their caps/bounds), while per-sample calibration enters only through a soft-field scale factor $s_{\text{soft},j}$ and a target mean connectivity $w_{\text{target},j}$, which sets the logistic offset α_j for a given β.

To connect the model to experiment, we jointly calibrate $\boldsymbol{\theta}_{\text{phys}}$ and $\{\boldsymbol{\theta}_{\text{img},j}\}$ by minimizing an absolute least-squares misfit between simulated and measured effective conductivities,

$$E(\boldsymbol{\theta}_{\text{phys}}, \{\boldsymbol{\theta}_{\text{img},j}\}) = \sum_j \left[\kappa_{\text{eff},x}^{\text{sim}}(j) - \kappa_{\text{eff}}^{\text{exp}}(j)\right]^2, \tag{32}$$

subject to physical bounds on all parameters. The bounded parameter space is explored with a stochastic global–local search. In a first stage, we fit $\boldsymbol{\theta}_{\text{phys}}$ using only the GNP-only composites (2 wt% and 4 wt% GNP), yielding a best-fit shared GNP parameter set and corresponding $\boldsymbol{\theta}_{\text{img},j}$ that reproduce the measured GNP-only conductivities within uncertainty. In a second stage, these shared GNP parameters are held fixed and the calibration is extended to the hybrid EG+GNP composites by scanning over κ_{worm} (with per-sample $\boldsymbol{\theta}_{\text{img},j}$ for the hybrid scans), retaining only parameter sets that simultaneously match both the GNP-only and hybrid conductivities. This two-stage procedure ensures that a single $\boldsymbol{\theta}_{\text{phys}}$ explains conductivity trends across all GNP-containing samples, while per-sample factors capture only modest scan-specific variations.

S8.2 From joint fits to worm-phase conductivity

Within the shared parameter set $\boldsymbol{\theta}_{\text{phys}}$, the only quantity that directly controls heat transport inside the EG phase is the worm conductivity κ_{worm}. All other shared parameters govern either the paraffin+GNP mixture (through $\kappa_{\|,\text{nom}}$, $\kappa_{\perp,\text{base}}$, γ and the conductivity caps) or the way the soft-GNP field is mapped into connectivity (through β and the mixture-law interpolation). Because the worm geometry itself is resolved voxel by voxel from microCT, the combination of the FVM and the measured effective conductivities allows us to invert for an effective worm-phase conductivity that is consistent with the observed behavior of worm-containing samples.

For each hybrid composition, we apply the same microCT and post-processing pipeline used for the GNP-only samples. A pore mask and EG worm mask are extracted from the intensity field, and a soft-GNP occupancy field is constructed in the remaining solid using the GMM-based approach. The voxel-level conductivity tensors in the paraffin+GNP regions are then computed using the previously calibrated GNP mixture parameters, while all voxels identified as part of the EG worms are assigned a single unknown worm conductivity κ_{worm}. For any trial value of κ_{worm} in a prescribed range of $20 - 225\ Wm^{-1}K^{-1}$, we run the FVM on each hybrid RVE and obtain simulated effective conductivities $\kappa_{\text{eff},x}^{\text{sim,hyb}}(\kappa_{\text{worm}})$.

To infer κ_{worm}, we compare these simulated conductivities to the experimentally measured values $\kappa_{\text{eff}}^{\text{exp}}$ for the hybrid composites and seek values of κ_{worm} that bring them into agreement. In practice, we evaluate a simple one-dimensional misfit function over the hybrid samples,

$$E_{\text{worm}}(\kappa_{\text{worm}}) = \sum_{j\in\mathcal{H}} \left[\kappa_{\text{eff},x}^{\text{sim,hyb}}(j;\kappa_{\text{worm}}) - \kappa_{\text{eff}}^{\text{exp}}(j)\right]^2, \tag{33}$$

where $\mathcal{H}$ denotes the set of worm-containing hybrids, and scan κ_{worm} over a grid. The minimizer of E_{worm} defines our best estimate of the worm-phase conductivity, and the range of κ_{worm} values for which E_{worm} remains near this minimum provides an uncertainty band that reflects both experimental uncertainty and the residual modeling error in the GNP-only calibration.

S8.3 Uncertainty propagation through the calibration

To quantify how experimental uncertainty in the measured effective conductivities propagates into the calibrated model parameters and ultimately into the inferred worm-phase conductivity, we repeat the joint calibration of Section S8.1 under systematically perturbed target conductivities. The experimental uncertainties $\delta_2 = 0.044\ \mathrm{W\,m^{-1}\,K^{-1}}$ and $\delta_4 = 0.076\ \mathrm{W\,m^{-1}\,K^{-1}}$ are taken from the PPMS-based analysis described in Section S2. In addition to the baseline targets $(\kappa_{2\text{wt}}^{\text{exp}}, \kappa_{4\text{wt}}^{\text{exp}})$, we consider four additional cases in which each target is independently shifted to its upper or lower 1σ bound,

$$\kappa_{2\text{wt}}^{(c)} = \kappa_{2\text{wt}}^{\text{exp}} \pm \delta_2, \qquad \kappa_{4\text{wt}}^{(c)} = \kappa_{4\text{wt}}^{\text{exp}} \pm \delta_4, \tag{34}$$

taken in all four combinations. These five cases sample the extremes of the experimentally admissible target conductivities for the two GNP-only compositions.

For each case, the same bounded stochastic search and least-squares objective (Eq. 32) are used to fit the shared physics parameters $\boldsymbol{\theta}_{\text{phys}}$ and per-scan factors $\boldsymbol{\theta}_{\text{img},j}$, with the parameter bounds widened modestly relative to the baseline fit to accommodate the shifted targets. All five fits converge to residuals well within $\pm 1\sigma$ of their respective targets, confirming that the constitutive model can accommodate the full range of experimentally admissible conductivities without systematic bias.

The five converged parameter sets are then propagated through the hybrid κ_{worm} inference described in Section 8.2. For each parameter set, the voxel-level conductivity tensors in the paraffin+GNP regions of every hybrid RVE are recomputed using the corresponding $\boldsymbol{\theta}_{\text{phys}}$, while all voxels belonging to the EG phase are assigned a trial worm conductivity κ_{worm}. The FVM solver is then run over the same κ_{worm} grid (20–225 $\mathrm{W\,m^{-1}\,K^{-1}}$ in 5 $\mathrm{W\,m^{-1}\,K^{-1}}$ steps) for each hybrid composition, and the one-dimensional misfit $E_{\text{worm}}(\kappa_{\text{worm}})$ (Eq. 33) is evaluated independently for each parameter set. Because the GNP mixture response varies across the five cases, the κ_{worm} value that minimizes E_{worm} shifts accordingly, producing a distribution of best-fit worm conductivities that reflects the experimental uncertainty in the GNP-only calibration. The overall uncertainty band is reported as $\kappa_{\text{worm}} = \kappa_{\text{worm,baseline}}\ (+\delta_{\text{upper}} / -\delta_{\text{lower}})$, where δ_{upper} and δ_{lower} are the largest positive and negative deviations from the baseline optimum across the five cases.

S9 Pore sensitivity analysis in the hybrid RVEs

Because the hybrid RVEs contain a small residual pore population outside the segmented EG phase, we tested whether these pores make a meaningful contribution to the modeled effective thermal conductivity. In the baseline model, pore voxels are assigned the conductivity of air, $\kappa_{\mathrm{air}} = 0.026\ \mathrm{W\,m^{-1}\,K^{-1}}$. For each hybrid composition, we then repeated the FVM calculation at the corresponding κ_{worm} intersection value after replacing all pore voxels with paraffin matrix, $\kappa_m = 0.25\ \mathrm{W\,m^{-1}\,K^{-1}}$, while keeping the EG skeleton, soft-GNP field, and all other calibrated constitutive parameters fixed. This toggle therefore isolates the effect of pore conductivity without altering the reconstructed hybrid architecture.
As shown in Figure S18, replacing all pore voxels with matrix produces only a negligible change in the modeled effective conductivity: H2 changes from 1.07 to 1.07 $\mathrm{W\,m^{-1}\,K^{-1}}$, H4 from 2.17 to 2.18 $\mathrm{W\,m^{-1}\,K^{-1}}$, and H5 from 2.67 to 2.68 $\mathrm{W\,m^{-1}\,K^{-1}}$. These correspond to relative changes of only +0.22%, +0.40%, and +0.25%, respectively. Thus, although pores are present in the reconstructed hybrid volumes, their thermal effect is negligible in the present framework. The hybrid conductivity enhancement is therefore not attributable to pore structure, but instead arises from the conductive solid architecture associated with the EG network and the paraffin+GNP phase.

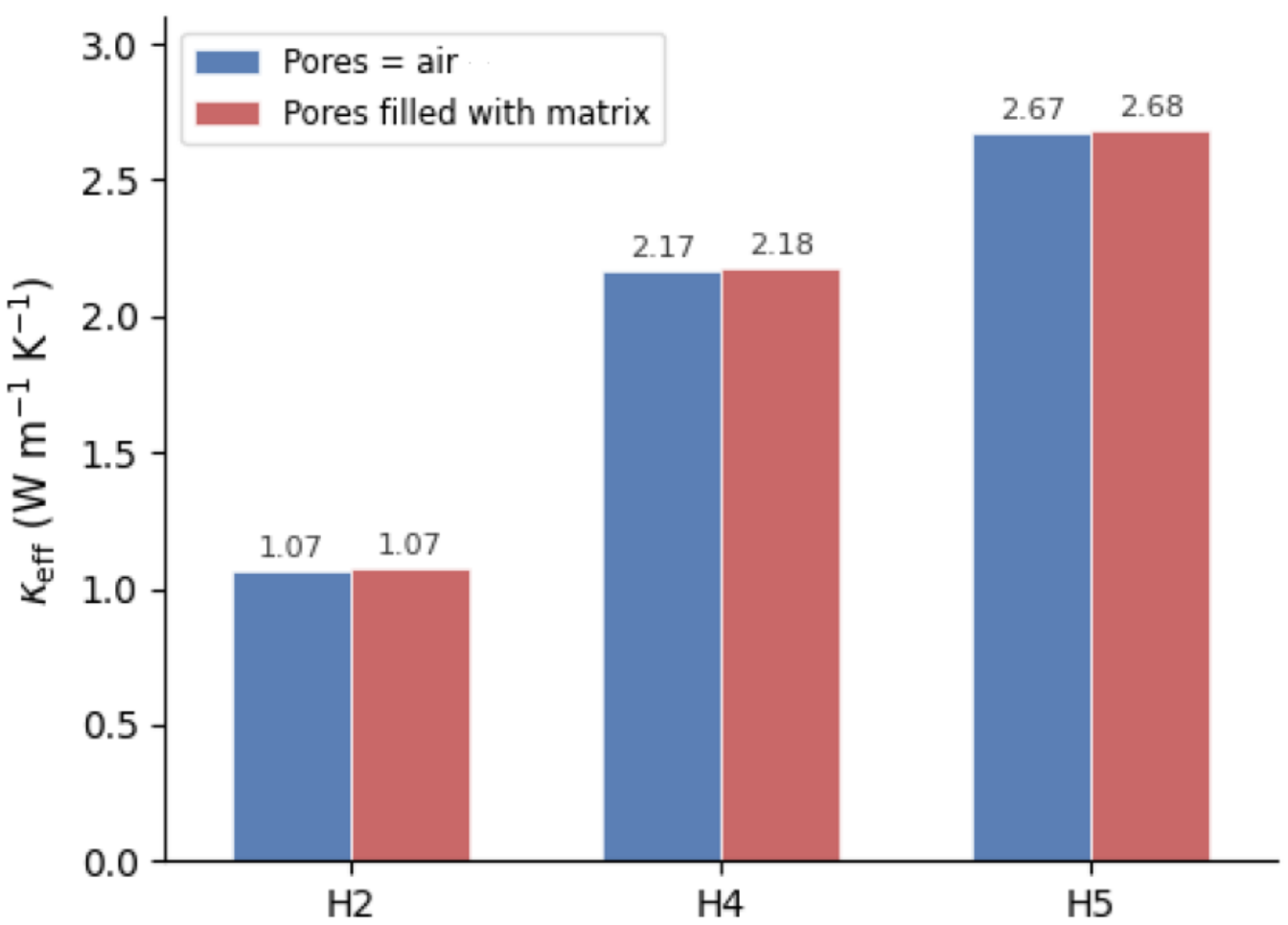

Figure S20: Effective thermal conductivity of hybrid samples with pore voxels treated as air (baseline) versus filled with matrix material

S10 Fixed-κ_{worm} cross-test and additive decomposition

To examine whether the hybrid conductivities can be explained by the presence of a spanning EG network alone, we performed a fixed-κ_{worm} cross-test on the H2, H4, and H5 hybrid RVEs. Instead of allowing κ_{worm} to vary with composition, it was fixed successively to the representative values inferred from H2, H4, and H5, namely $\kappa_{\text{worm}} \approx 72.6$, 169.1, and 182.6 W m^{-1} K^{-1}, and each fixed value was then applied across all three hybrid microstructures.
For each sample under each fixed κ_{worm}, four finite-volume cases were evaluated on the same microCT-derived geometry: (i) baseline (matrix + pores), (ii) GNP in matrix only, (iii) EG network only, and (iv) the full hybrid with both phases present. In all cases, the same RVE, pore treatment, voxel geometry, boundary conditions, and previously calibrated paraffin+GNP constitutive parameters were used; only the active EG- and GNP-mediated transport channels were changed.
The fixed-κ_{worm} results are summarized in Figure S21. No single composition-independent κ_{worm} reproduces the full H2/H4/H5 loading trend. Using the H2-derived value gives full-hybrid predictions of 1.07, 1.36, and 1.63 W m^{-1} K^{-1} for H2, H4, and H5, respectively (Figure S21a), which underpredict the higher-loading hybrids. The H4-derived value gives intermediate predictions of 1.44, 2.17, and 2.55 W m^{-1} K^{-1} (Figure S21b), but again does not reproduce all three samples simultaneously. Using the H5-derived value gives 1.49, 2.28, and 2.67 W m^{-1} K^{-1} (Figure S21c), which overpredicts H2 and slightly overpredicts H4. Thus, the increase in microCT-resolved connectivity alone does not collapse the hybrid response onto a single universal κ_{worm}.

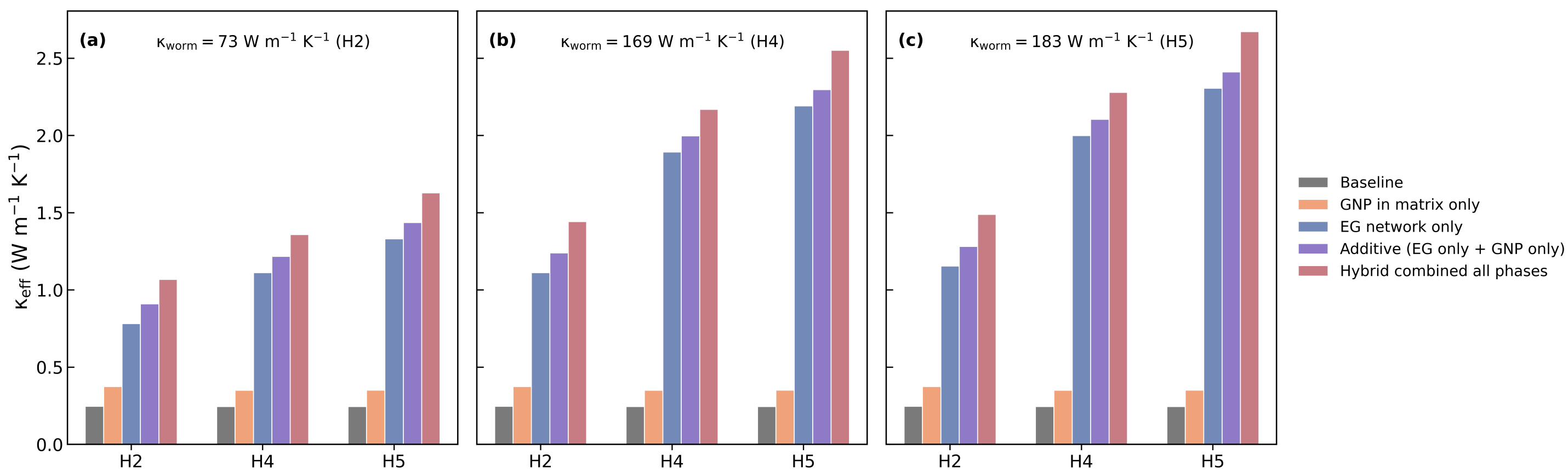

Figure S21: Fixed-κ_{worm} cross-test for the hybrid RVEs with κ_{worm} fixed to the value inferred from (a) H2, (b) H4, and (c) H5.

To assess additivity, we defined

$$\kappa_{\text{additive}} = \kappa_{\text{baseline}} + (\kappa_{\text{EG only}} - \kappa_{\text{baseline}}) + (\kappa_{\text{GNP only}} - \kappa_{\text{baseline}}), \tag{35}$$

and compared it with the fully coupled hybrid result, κ_{both}. The difference

$$\Delta\kappa_{\text{syn}} = \kappa_{\text{both}} - \kappa_{\text{additive}} \tag{36}$$

was taken as the synergy contribution.
Across all nine combinations of sample and fixed κ_{worm}, κ_{both} remained larger than κ_{additive}, with a positive synergy contribution of 0.141–0.260 W m^{-1} K^{-1}. Figure S22 shows the composition-matched decomposition, for which the EG-network contribution provides the dominant share of the conductivity increase from H2 to H5, the GNP-in-matrix contribution remains comparatively modest, and the synergy term remains positive for all three hybrids. These results show that percolation of the EG backbone is necessary but not sufficient to explain the measured hybrid conductivities: the EG network forms the dominant transport scaffold, but the full hybrid response is not captured by a purely additive sum of EG-network and GNP-matrix contributions.

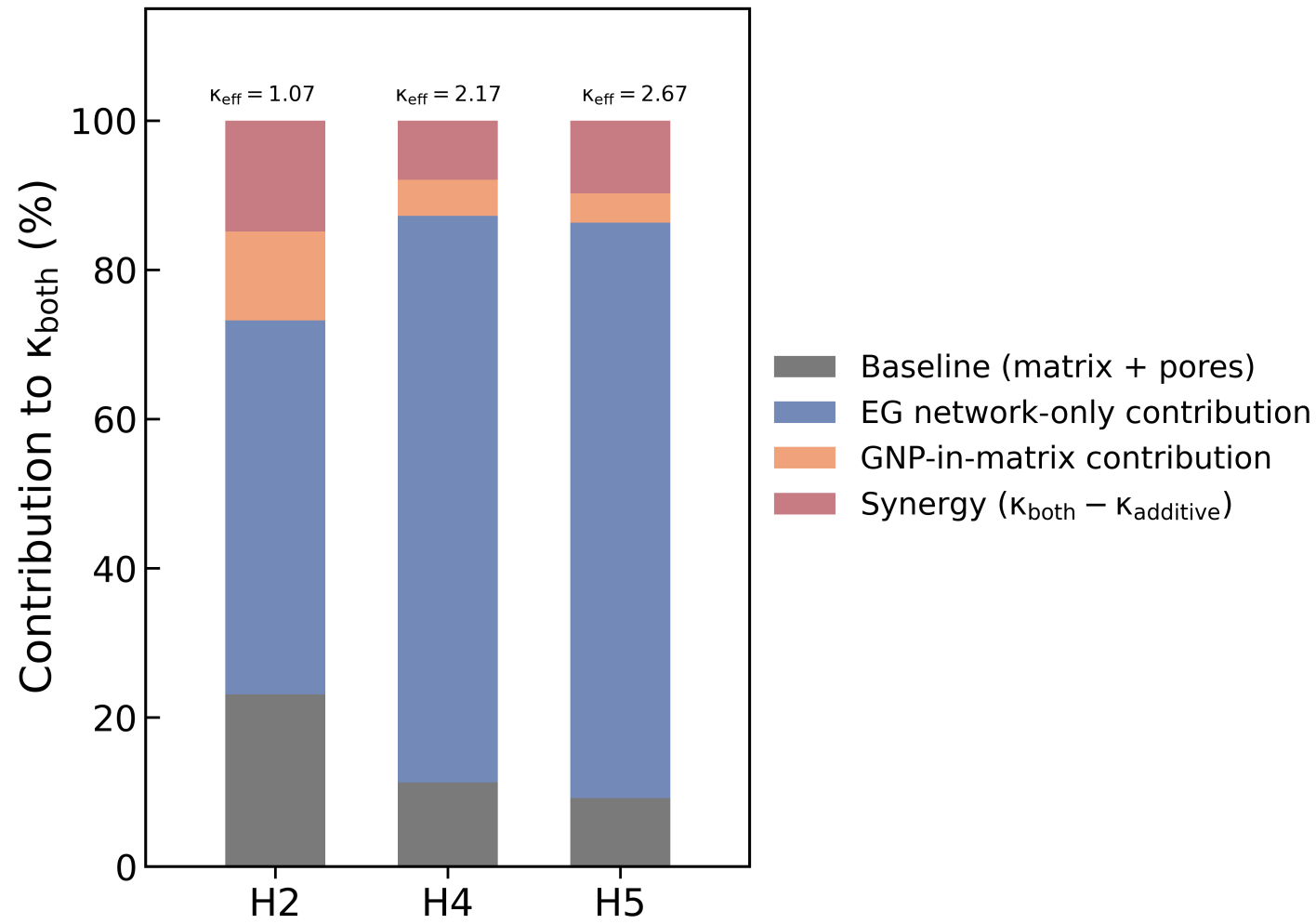

Figure S22: Composition-matched decomposition of the hybrid thermal conductivity into baseline, EG-network, GNP-in-matrix, and synergy contributions.

References

[1] Elham Fallahi, Mohammad Barmar, and Mohammad Haghighat Kish. Preparation of phase-change material microcapsules with paraffin or camel fat cores: Application to fabrics. *Iranian Polymer Journal*, 19(4):277–286, 2010.

[2] Zheng Bo, Xiaorui Shuai, Shun Mao, Huachao Yang, Jiajing Qian, Junhong Chen, Jianhua Yan, and Kefa Cen. Green preparation of reduced graphene oxide for sensing and energy storage applications. *Scientific Reports*, 4:4684, 04 2014.

[3] Mingjie Zheng and Weimin Du. Phase behavior, conformations, thermodynamic properties, and molecular motion of multicomponent paraffin waxes: A Raman spectroscopy study. *Vibrational Spectroscopy*, 40:219–224, 2006.

[4] Andrea C. Ferrari. Raman spectroscopy of graphene and graphite: Disorder, electron–phonon coupling, doping and nonadiabatic effects. *Solid State Communications*, 143:47–57, 2007.

[5] O. Maldonado. Pulse method for simultaneous measurement of electric thermopower and heat conductivity at low temperatures. *Cryogenics*, 32(10):908–912, 1992.

[6] Nobuyuki Otsu. A threshold selection method from gray-level histograms. *IEEE Transactions on Systems, Man, and Cybernetics*, 9(1):62–66, 1979.

[7] Ping-Sung Liao, Tse-Sheng Chen, and Pau-Choo Chung. A fast algorithm for multilevel thresholding. *Journal of Information Science and Engineering*, 17(5):713–727, 2001.

[8] Stéfan van der Walt, Johannes L. Schönberger, Juan Nunez-Iglesias, François Boulogne, Joshua D. Warner, Neil Yager, Emmanuelle Gouillart, Tony Yu, and the scikit-image contributors. scikit-image: Image processing in python. *PeerJ*, 2:e453, 2014.

[9] Christopher M. Bishop. *Pattern Recognition and Machine Learning*. Information Science and Statistics. Springer, 2006.

[10] Arthur P. Dempster, Nan M. Laird, and Donald B. Rubin. Maximum likelihood from incomplete data via the EM algorithm. *Journal of the Royal Statistical Society: Series B (Methodological)*, 39(1):1–38, 1977.

[11] P. Majumder and A. Bhattacharyya. On the anisotropic thermal conductivity of shape memory alloy single crystals. *Acta Mechanica*, 193:151–176, 2007.

[12] M. Jiang, I. Jasiuk, and M. Ostoja-Starzewski. Apparent thermal conductivity of periodic two-dimensional composites. *Computational Materials Science*, 25(3):329–338, 2002.

[13] R. Hill. The elastic behaviour of a crystalline aggregate. *Proceedings of the Physical Society. Section A*, 65(5):349–354, 1952.

[14] J. Peter Watt and Louis Peselnick. Clarification of the hashin–shtrikman bounds on the effective elastic moduli of polycrystals with hexagonal, trigonal, and tetragonal symmetries. *Journal of Applied Physics*, 51(3):1525–1531, March 1980.

[15] Matthias Kabel, Dennis Merkert, and Matti Schneider. Use of composite voxels in fft-based homogenization. *Computer Methods in Applied Mechanics and Engineering*, 294:168–188, 2015.

[16] Linzhi Wu. Bounds on the effective thermal conductivity of composites with imperfect interface. *International Journal of Engineering Science*, 48:783–794, 2010.

[17] Z. Hashin and S. Shtrikman. A variational approach to the theory of the effective magnetic permeability of multiphase materials. *Journal of Applied Physics*, 33(10):3125–3131, October 1962.

[18] Anders Clausen, Niels Aage, and Ole Sigmund. Topology optimization of coated structures and material interface problems. *Computer Methods in Applied Mechanics and Engineering*, 290:524–541, 2015.

[19] Atsushi Kawamoto, Tadayoshi Matsumori, Shintaro Yamasaki, Tsuyoshi Nomura, Tsuguo Kondoh, and Shinji Nishiwaki. Heaviside projection based topology optimization by a pde-filtered scalar function. *Structural and Multidisciplinary Optimization*, 44(1):19–24, 2011.

[20] Ole Sigmund. Morphology-based black and white filters for topology optimization. *Structural and Multidisciplinary Optimization*, 33(4-5):401–424, 2007.

[21] Nikolay Kyurkchiev and Svetoslav Markov. *Sigmoid Functions: Some Approximation and Modelling Aspects*. LAP LAMBERT Academic Publishing, Saarbrücken, Germany, 2015.

[22] J. Ordonez-Miranda, Ronggui Yang, and J. J. Alvarado-Gil. A crowding factor model for the thermal conductivity of particulate composites at non-dilute limit. *Journal of Applied Physics*, 114(6):064306, 2013.

[23] Xiaojuan Tian, Mikhail E. Itkis, Elena B. Bekyarova, and Robert C. Haddon. Anisotropic thermal and electrical properties of thin thermal interface layers of graphite nanoplatelet-based composites. *Scientific Reports*, 3:1710, 2013.

[24] Ce-Wen Nan, R. Birringer, David R. Clarke, and H. Gleiter. Effective thermal conductivity of particulate composites with interfacial thermal resistance. *Journal of Applied Physics*, 81(10):6692–6699, 1997.

[25] D. P. H. Hasselman and Lloyd F. Johnson. Effective thermal conductivity of composites with interfacial thermal barrier resistance. *Journal of Composite Materials*, 21(6):508–515, 1987.

[26] Fan Yang, Teruyuki Ikeda, G. Jeffrey Snyder, and Chris Dames. Effective thermal conductivity of polycrystalline materials with randomly oriented superlattice grains. *Journal of Applied Physics*, 108(3):034310, 2010.

[27] Jan Stranský, Jan Vorel, Jan Zeman, and Michal Šejnoha. Mori-tanaka based estimates of effective thermal conductivity of various engineering materials. *Micromachines*, 2(2):129–149, 2011.

[28] Yue Schuman. Thermal analysis of phase change materials: Three organic waxes using tga, dsc, and modulated dsc. Application Note TA405, TA Instruments, 2020. Available at www.tainstruments.com.

[29] Yanqi Zhao, Lu Jin, Boyang Zou, Geng Qiao, Tongtong Zhang, Lin Cong, Feng Jiang, Chuan Li, Yun Huang, and Yulong Ding. Expanded graphite – paraffin composite phase change materials: Effect of particle size on the composite structure and properties. *Applied Thermal Engineering*, 171:115015, 2020.

[30] Jörg Petrasch, Birte Schrader, Peter Wyss, and Aldo Steinfeld. Tomography-based determination of the effective thermal conductivity of fluid-saturated reticulate porous ceramics. *Journal of Heat Transfer*, 130(3):032602, 2008.

[31] Wenlong Tian, Lehua Qi, Xujiang Chao, Junhao Liang, and M. W. Fu. Numerical evaluation on the effective thermal conductivity of the composites with discontinuous inclusions: Periodic boundary condition and its numerical algorithm. *International Journal of Heat and Mass Transfer*, 134:735–751, 2019.

[32] T. Kanit, S. Forest, I. Galliet, V. Mounoury, and D. Jeulin. Determination of the size of the representative volume element for random composites: statistical and numerical approach. *International Journal of Solids and Structures*, 40:3647–3679, 2003.

[33] Fady Moukalled, Luca Mangani, and M. Darwish. *The Finite Volume Method in Computational Fluid Dynamics: An Advanced Introduction with OpenFOAM® and Matlab®*, volume 113 of *Fluid Mechanics and Its Applications*. Springer, Cham, Switzerland, 2016.

[34] Yousef Saad. *Iterative Methods for Sparse Linear Systems*. Society for Industrial and Applied Mathematics (SIAM), Philadelphia, PA, 2 edition, 2003.

[35] Ryoto Okuta, Yusuke Unno, Daisuke Nishino, Shinji Hido, and Chihiro Loomis. CuPy: A NumPy-compatible library for NVIDIA GPU calculations. In *Proceedings of Workshop on Machine Learning Systems (LearningSys) at NeurIPS*, 2017.

[36] Pauli Virtanen, Ralf Gommers, Travis E. Oliphant, Matt Haberland, Tyler Reddy, David Cournapeau, Evgeni Burovski, Pearu Peterson, Warren Weckesser, Jonathan Bright, Stéfan J. van der Walt, Matthew Brett, Joshua Wilson, K. Jarrod Millman, Nikolay Mayorov, Andrew R. J. Nelson, Eric Jones, Robert Kern, Eric Larson, C. J. Carey, İlhan Polat, Yu Feng, Eric W. Moore, Jake VanderPlas, Denis Laxalde, Josef Perktold, Robert Cimrman, Ian Henriksen, E. A. Quintero, Charles R. Harris, Anne M. Archibald, Antônio H. Ribeiro, Fabian Pedregosa, and Paul van Mulbregt. SciPy 1.0: Fundamental algorithms for scientific computing in Python. *Nature Methods*, 17(3):261–272, 2020.

[37] Karol Pietrak and Tomasz S. Wiśniewski. A review of models for effective thermal conductivity of composite materials. *Journal of Power Technologies*, 95(1):14–24, 2015. Open Access.

[38] James Clerk Maxwell. Conduction through heterogeneous media. In *A Treatise on Electricity and Magnetism*. Clarendon Press, Oxford, 1873. Chapter IX; dilute spheres result. Modern reprint chapter DOI: 10.1017/CBO9780511709333.025.

[39] R. C. Progelhof, J. L. Throne, and R. R. Ruetsch. Methods for predicting the thermal conductivity of composite systems: A review. *Polymer Engineering & Science*, 16(9):615–625, 1976.

[40] Guo Chang, Shuang Zhang, Kaiyun Chen, Wei Zhang, Liang Li, Yongjian Zhang, Haoran Peng, Dongxiao Kan, Luhua Wang, Hailong Zhang, and Wangtu Huo. Achieving excellent thermal transport in diamond/cu composites by breaking bonding strength-heat transfer trade-off dilemma at the interface. *Composites Part B: Engineering*, 289:111925, 2025.

[41] Zahra Ebrahim Nataj, Youming Xu, Dylan Wright, Jonas O. Brown, Jivtesh Garg, Xi Chen, Fariborz Kargar, and Alexander A. Balandin. Cryogenic characteristics of graphene composites—evolution from thermal conductors to thermal insulators. *Nature Communications*, 14:3190, 2023.

[42] Bohayra Mortazavi, Hongliu Yang, Farzad Mohebbi, Gianaurelio Cuniberti, and Timon Rabczuk. Graphene or h-bn paraffin composite structures for the thermal management of li-ion batteries: A multiscale investigation. *Applied Energy*, 202:323–334, 2017.
